# Identifying Neural State Changes due to Gain versus Off-Manifold Displacement

**Published:** 2026-09-18

**Authors:** Sam McKenzie

**Affiliations:** 1. University of New Mexico Health Sciences Center, Department of Neuroscience, Albuquerque, NM 87131

## Abstract

Memory segmentation is thought to be caused by rapid decorrelation in neural activity, commonly quantified by Euclidean distance and cosine angle. While these metrics are useful for detecting that a transition has occurred, they cannot reveal the nature of that change relative to the repertoire of pre-existing states – a space known as the neural manifold. This distinction matters, since neuromodulators that are thought to drive state transitions also influence neuronal excitability, and there is ongoing controversy over whether learning involves repurposing old representations or creating new ones. Here, I introduce a decomposition that separates changes in neural activity into those that can be understood by gain modulation of a nearby manifold point versus genuine off-manifold displacement. The approach exploits the privileged role of the radial axis in neural population activity by partitioning the normal space of a local region of the neural manifold. A central challenge is identifiability: from a static reference manifold and a single test state, the state from which a perturbation began, its gain magnitude, and, in general, the mechanistic decomposition of the observed displacement cannot be uniquely recovered. I therefore formulate identifiability as a cascade of geometric gates that specify the conditions under which each component can be interpreted. These gates distinguish structural failures, such as the absence of a local manifold chart or incorrect intrinsic dimensionality, from estimation error and systematic bias arising from the manifold reference, gain-axis misalignment, and poor ratio conditioning. I derive exact expressions for how reference error propagates into the reported components and through simulations on idealized manifolds show that several factors—neighborhood size, curvature, sampling density, ambient dimension, and noise—affect the decomposition through a small set of geometric quantities. This framework therefore does not simply assign a neural state change to gain or novelty; it specifies when those assignments are identifiable, how they become biased, and which diagnostics can reveal the relevant failure regime. By quantifying the nature of the neural state change, not just its magnitude, this framework provides a more interpretable description of population dynamics and a principled basis for evaluating candidate mechanisms underlying neural state transitions.

## 1 Introduction

Though the external world arrives to our senses as a continuous stream, it is recorded in episodic memory in discretized chunks bounded by salient moments of change^1–4^. This segmentation is thought to be driven by rapid neuronal decorrelation facilitated by phasic release of neuromodulators, such as norepinephrine^5,6^. When neural states change in response to novelty, it is not always the case that a never-before-seen stimulus produces a never-before-seen brain state^7,8^. We understand the future through the lens of the past, and the brain maintains powerful neural attractors to categorize the new by the old^9,10^. Moreover, the same neuromodulators that catalyze state transitions increase neuronal excitability, and indeed such gain modulation is thought to be a driving force behind state switching^5^. Therefore, when neuronal activity shows rapid transitions, it is important to know if these changes reflect movements within the pre-existing state space, genuine creation of a novel representation, or mere amplification of a prior state.

Cosine and Euclidean distances are standard metrics used to quantify neuronal state similarity. While useful in detecting whether a state change occurred, these metrics cannot resolve how the displaced neural state relates to a reference manifold. Differential geometry provides a mathematical language for describing the local state space through its tangent and normal bundles. What is missing is the privileged role of gain amplification, and more precisely whether one should consider a familiar gain-modulated state to be on or off manifold. In recognition of the central role that multiplicative gain modulation plays in neuronal coding, the proposed decomposition further partitions the normal space into a radial gain direction and its orthogonal complement.

The goal then is to apportion a neural state change into that which is on the manifold of existing states, that which is attributable to multiplicative gain of a pre-existing state, and a residual that is off-manifold, in directions the sampled reference does not span. Such an analysis raises important questions of identifiability, since gain is defined only relative to a reference state, and that reference is not generally recoverable from a single observation. Here I provide a systematic account of how error in the reference sample, the intrinsic dimension, the tangent frame, the anchor and the gain axis propagates into the reported components, and of which of those conditions can be checked from the data in hand.

## 2 The decomposition

### 2.1 The tangent and normal bundles:

**Figure 1.**
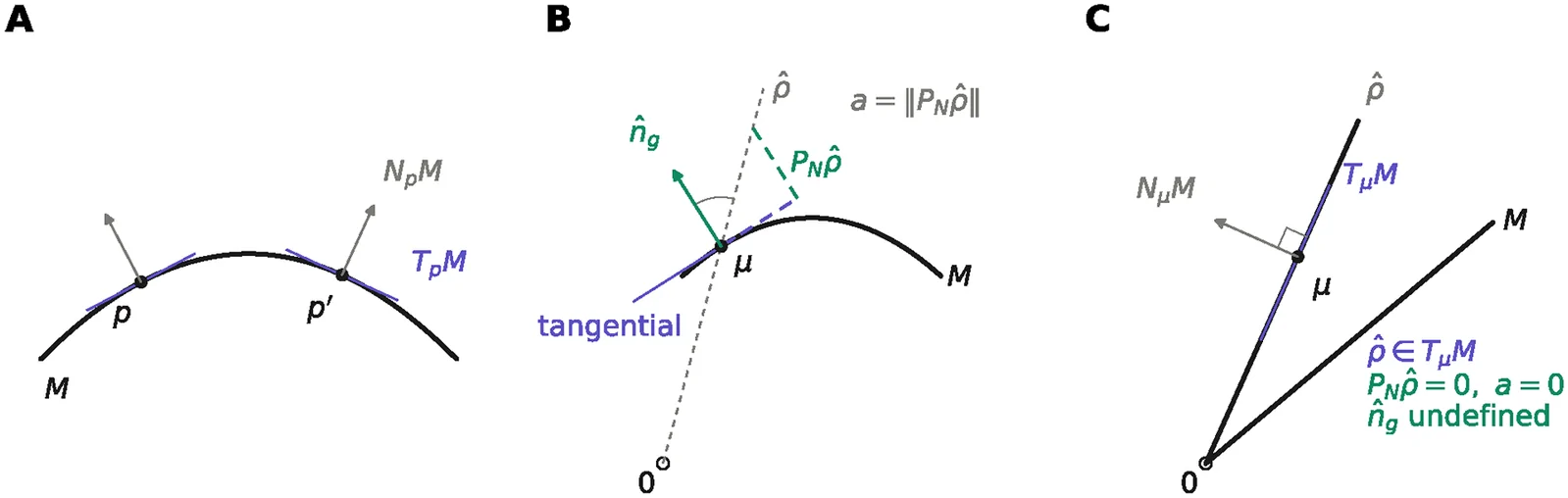
The gain axis and where it fails to exist. **(A)** Tangent and normal bundles at two points of a curved reference manifold. The partition into tangent and normal directions is defined pointwise and changes with position, which is what makes it a bundle rather than a fixed frame. **(B)** Construction of the gain axis. The radial direction $\overset{ˆ}{\rho }$ from the zero-activity origin is resolved at μ into a tangential part, which is discarded, and its projection into the normal space, whose normalization is the gain axis ${\hat{n}}_{g}$. The alignment $a=\left\|P_{N}\overset{ˆ}{\rho }\right\|$ is the cosine of the marked angle. Defining the axis with $\overset{ˆ}{\rho }$ alone would conflate radial change with tangential movement. **(C)** The degenerate case. On a cone through the origin, every displacement moves equivalently along the radial direction and the tangent direction and the gain axis is undefined.

Differential geometry describes an embedded manifold through its tangent and normal bundles (Figure 1A), which at each point p partition the ambient space into directions tangent and orthogonal to the manifold:

(1) $$R^{F}=T_{p}ℳ⊕N_{p}ℳ,$$

the tangent space $T_{p}ℳ$ contains the locally admissible tangent directions of the manifold at point p, and the normal space $N_{p}ℳ$ contains directions that are orthogonal to those tangent directions. Let ℳ be a reference manifold, sampled as a point set $Z_{A}\subset R^{F}$, and let $z_{B}$ be a test point. We wish to know whether $z_{B}$ is on-manifold, a gain-modulated pre-existing point, or truly off-manifold. From static geometry we cannot know which point was gain-modulated, and we are left with a choice of where to anchor the gain estimate. A natural choice is the nearest on-manifold point, and it is from this anchor that the manuscript explores what is knowable and where the biases lie. Let p∈M be a reference point on the manifold with p≠0. The projectors onto the tangent and normal spaces at p are

(2) $$P_{T}(p)=\text{proj onto}\;T_{p}M,\;P_{N}(p)=I-P_{T}(p)$$

Neuroscience accords privileged status to the radial axis, and to the origin as a meaningful zero, since multiplicative gain is not usually assumed to change what is being coded but rather the intensity of that code. The unit radial axis and gain within the normal bundle are defined as

(3) $${\hat{\rho }}_{p}=p/\|p\|,a(p)=\left\|P_{N}(p){\hat{\rho }}_{p}\right\|\in [0,1]$$

Accounting for this gain axis, the decomposition further partitions the normal space into a gain-modulated component and the off-manifold residual,

(4) $$N_{p}ℳ=\text{span}\left(P_{N}(p){\hat{\rho }}_{p}\right)⊕N_{\text{res}}(p)$$

The gain direction as it appears within the normal space is

(5) $${\hat{n}}_{g}(p)=\frac{P_{N}(p){\hat{\rho }}_{p}}{a(p)},\;\text{defined iff}\;a(p)>0$$

Defining the gain axis this way, rather than with $\hat{\rho }$ alone, matters since some of that energy may otherwise be conflated with tangential movement along the manifold (Figure 1B). Since ${\hat{n}}_{g}(p)\in N_{p}ℳ$ by construction, the three subspaces $T_{p}ℳ,\text{span}\left({\hat{n}}_{g}(p)\right)$, and $N_{\text{res}}(p)$ are mutually orthogonal and the decomposition does not depend on the order in which the projections are applied. Writing $r_{p}=z_{B}-p$ for the displacement from the manifold reference point, and $P_{\text{res}}(p)=P_{N}(p)-{\hat{n}}_{g}(p){\hat{n}}_{g}(p{)}^{⊤}$ for the projector onto $N_{\text{res}}(p)$, the decomposition can be written,

(6) $$r_{p}=\underset{\text{on-manifold pattern}}{\underbrace{P_{T}(p)r_{p}}}+\underset{\text{gain}}{\underbrace{\left({\hat{n}}_{g}(p{)}^{⊤}r_{p}\right){\hat{n}}_{g}(p)}}+\underset{\text{off-manifold}}{\underbrace{P_{res}(p)r_{p}}}$$

We report the three energy fractions

(7) $$T=\frac{{\left\|P_{T}(p)r_{p}\right\|}^{2}}{{\left\|r_{p}\right\|}^{2}},\;G=\frac{{\left({\hat{n}}_{g}(p{)}^{⊤}r_{p}\right)}^{2}}{{\left\|r_{p}\right\|}^{2}},\;O=\frac{{\left\|P_{res}(p)r_{p}\right\|}^{2}}{{\left\|r_{p}\right\|}^{2}},\;G+T+O\equiv 1$$

The novel contribution is the partition of the normal space into a radial direction and its complement, which is what makes gain separable from novelty. When the radial direction lies completely within the tangent space (as on a cone through the origin), it has no normal component and the split between gain and off-manifold displacement is undefined (Figure 1C).

Equation 6 is stated for a manifold point p with its true tangent space and radial direction. In an experimental setting, none of those is observed and estimators are needed. To identify the anchor, find the k-nearest neighbors of $z_{B}$ in $Z_{A}$, giving a local neighborhood $N_{A}$ with centroid μ and mean-centered matrix $X_{c}=N_{A}-\mu$. Let $V_{d}$ be the leading d right singular vectors of $X_{c}$, an estimate of the local tangent space. The tangential extent of the neighborhood is ${↕}^{2}=\Sigma_{j}{\left\|V_{d}^{⊤}\left(x_{j}-\mu \right)\right\|}^{2}/(k-1)$; normalizing by k instead biases ${↕}^{2}$ low by (k-1)/k. Therefore, the estimator substitutes: a reference sample $Z_{A}$ for ℳ, the k-nearest-neighbor centroid μ for p, the leading d right singular vectors $V_{d}$ for $T_{p}ℳ$, and ${\hat{\rho }}_{\mu }=\mu /\|\mu \|$ for the radial direction at p. Sections 3 and 5 are about the biases introduced by these substitutions: which of the four substitutions is consequential, in what order the errors propagate, and which of them can be checked from the data at hand. Reference sampling and the full estimator construction are detailed in Supplement Section 1.

## 3 What is identifiable from static manifold geometry

Static geometry provides information about the displacement from the observable local anchor μ, but not generally about the displacement from the state m at which the perturbation began. We therefore distinguish anchor-relative geometric components from perturbation-relative mechanistic components. Often one wants the total between two states, $z_{B}-m$, written here with $r=z_{B}-\mu$ the displacement from the anchor:

(8) $$z_{B}-m=\underset{\text{anchor drift}}{\underbrace{\left(\mu -m\right)}}+\underset{\text{residual tangent}}{\underbrace{r_{\text{tan}}}}+\underset{\text{residual gain}}{\underbrace{\left({\hat{n}}_{g}^{⊤}r\right){\hat{n}}_{g}}}+\underset{\text{residual novelty}}{\underbrace{r_{\text{novel}}}}$$

Since in the current construction m is unavailable, the present decomposition reports what may be learned about deviations from the anchor μ, and the relevance of that anchor in determining the validity of off-manifold energy is a modelling choice. We do not estimate a gain magnitude. The scalar λ for which $z_{B}\approx \lambda m$, and the manifold state m from which the change proceeded, are not identifiable from static geometry. A change may mix radial, tangential and off-manifold components, so m cannot be read off $z_{B}$; and even for a purely radial change, where the same ray meets the manifold twice (m and λm both on it), the state λm at unit gain and the state m amplified by λ produce the same observation (Figure 3B).

### 3.1 The anchor is selected by the test point, not by the base point

**Figure 2.**
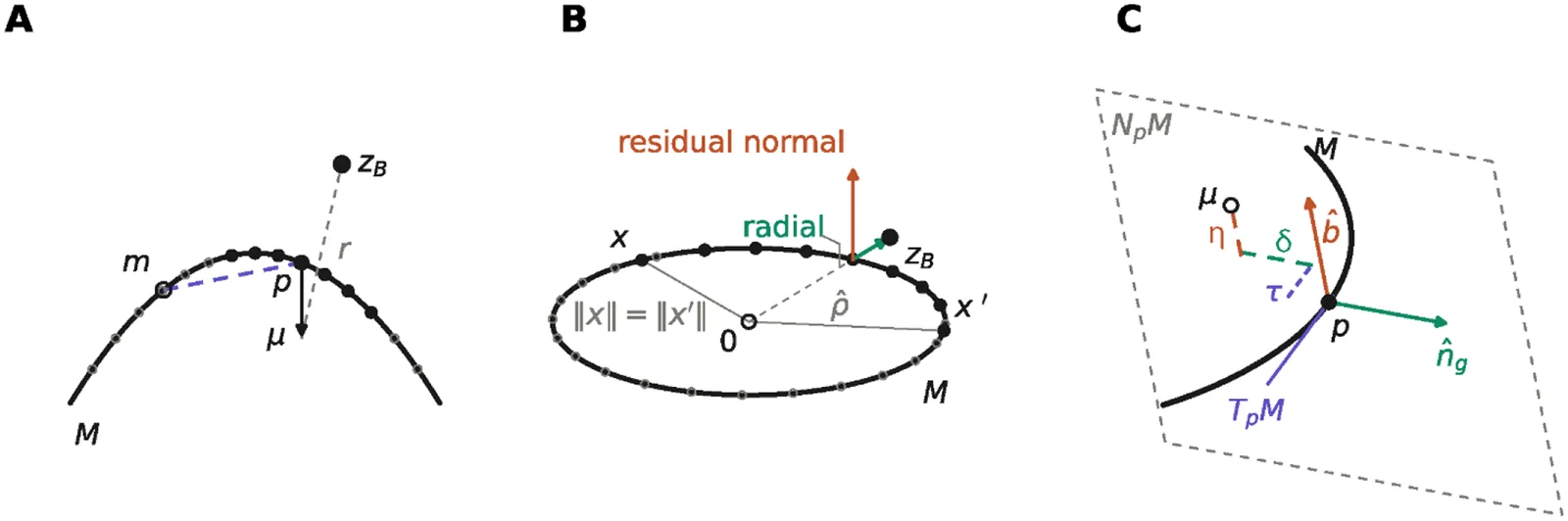
Anchor selection, the anchor frame, and when the anchor is exact. **(A)** The test point selects its own anchor. μ is the mean of the k reference points nearest $z_{B}$, so μ-m decomposes into a tangential leg p-m, which tracks movement along the manifold, and a normal leg μ-p, which is the sagitta. **(B)** The two cases in which the tangential leg vanishes exactly. On a circle centered on the origin, a displacement out of the plane of the reference set is orthogonal to every x, while a radial displacement also has no tangent component. **(C)** The anchor frame. The anchor displacement resolves into δ along the gain axis, η in the residual normal space, and τ in the tangent space. Drawn on a curve in three dimensions, where the normal space is a genuine plane and β is a real direction.

Equation (8) writes the anchor drift as μ-m, where the anchor μ is the centroid of the k nearest neighbors of $z_{B}$. Changing the imposed displacement can therefore change which reference points are selected, and the estimator is a nonlinear function of the very quantity it is being used to decompose (Figure 2A). Write $p=\pi_{ℳ}\left(z_{B}\right)$ for the projection of the test point onto the local manifold and split the drift accordingly:

(9) $$\mu -m=\underset{\text{selection-referred anchor bias}}{\underbrace{\left(\mu -p\right)}}+\underset{\text{change-dependent projection shift}}{\underbrace{\left(p-m\right)}}$$

The two terms have different characters. The first is a property of the neighborhood geometry around the point the probe actually sits above, and it is what Proposition 2 of Section 5.4 derives. The second is a property of the change itself: it is nonzero exactly when the imposed displacement has a component along the manifold, and it is therefore the same phenomenon as the anchor tracking described at the start of Section 5; in the present construction such tangential movement is absorbed through the selection of a closer anchor.

#### 3.1.1 When the second term vanishes.

Two cases make p=m exactly, each sufficient (Figure 2B). Case 1: If the displacement lies orthogonal to the span of the reference manifold. Case 2: If the displacement is radial and the reference set lies on an origin-centered sphere of radius R, every reference point has the same norm, so for $z_{B}=\lambda m$ with λ>0 the squared distance $\left(\lambda^{2}+1\right)R^{2}-2\lambda m^{⊤}x$ to a reference point x decreases monotonically in $m^{⊤}x$ and the ordering is unchanged. The radial and ambient validation probes of Section 5 use exactly these two cases, so their neighborhoods are invariant to both gain and novelty and p=m. Those results therefore isolate μ-p with the second term in (9) exactly zero. The on-manifold probes of Section 5 instead refer the anchor displacement to the probe’s own projection, which removes the second term by construction. Outside these constructions the projection shift is not characterized and requires additional information about the system.

### 3.2 How anchor misspecification contributes bias

Equation (8) identifies anchor drift but does not show how it changes the reported fractions. We can account for that effect by working in the frame used by the estimator: the unit gain direction within the normal space ${\hat{n}}_{g}$, the tangent basis $V_{d}$, and the off-manifold residual normal space orthogonal to both.

Let m be the true base manifold point and μ the estimated anchor. Decompose the anchor displacement as

(10) $$m-\mu =\delta \;{\hat{n}}_{g}+\vec{\eta }+P_{T}\left(m-\mu \right),\;\vec{\eta }=P_{\text{res}}\left(m-\mu \right),\;\eta =\left\|\vec{\eta }\right\|,\;\tau^{2}={\left\|P_{T}(m-\mu )\right\|}^{2}$$

Here $\delta ={\hat{n}}_{g}^{⊤}(m-\mu )$ is the signed anchor displacement along the normal-space gain direction, η is its residual-normal component, and $\tau^{2}$ is the squared magnitude of its tangential component (Figure 2C). Write the imposed change as $z_{B}-m=\gamma {\hat{n}}_{g}+\beta \hat{b}$, where γ is the imposed displacement along the normal-space gain direction, β the imposed residual-normal component, and $\hat{b}$ a unit vector in the residual normal space. Here ${\hat{n}}_{g}$ and $\hat{b}$ are taken in the anchor frame at μ. The resulting residual $r=z_{B}-\mu$ is then resolved exactly into gain, residual-normal and tangent components.

#### 3.2.1 Proposition 1 (exact decomposition of the fractions).


(11) $$\|r{\|}^{2}=(\gamma +\delta {)}^{2}+\beta^{2}+\eta^{2}+2\beta \eta \;\text{cos}\;\psi +\tau^{2},\;\text{cos}\;\psi =\hat{b}\cdot \hat{\eta }$$

(12) $$G=\frac{(\gamma +\delta {)}^{2}}{\|r{\|}^{2}},\;O=\frac{\beta^{2}+\eta^{2}+2\beta \eta \;\text{cos}\;\psi }{\|r{\|}^{2}},\;T=\frac{\tau^{2}}{\|r{\|}^{2}}$$

This identity follows only from the mutual orthogonality of the gain, residual-normal and tangent subspaces. It therefore provides an exact accounting of how anchor drift changes the reported fractions, independently of the approximations used below. The proof is given in Supplement Section 1, and Table S1 verifies the identity numerically at machine precision. Proposition 1 assumes that the imposed change has no component in the anchor frame’s tangent space; in general the tangential term is ${\left\|P_{T}\left(z_{B}-m\right)+P_{T}(m-\mu )\right\|}^{2}$ in place of $\tau^{2}$, and Table S1 checks this general form.

Several useful consequences follow; (ii)–(iv) are derived in the Supplement.

*(i) The ideal case*. Where the anchor lies on the correct manifold point, δ=η=τ=0 and the veridical off-manifold novelty is $O_{⋆}=\beta^{2}/\left(\gamma^{2}+\beta^{2}\right)$.

*(ii) First order*. Ordering terms in the neighborhood extent — δ and η are $O\left({↕}^{2}\right),\tau^{2}$ is $O\left({↕}^{2}/k\right)$, and $\delta^{2}$ and $\eta^{2}$ are $O\left({↕}^{4}\right)$ and dropped — the bias in the off-manifold estimate is,

(13) $$O-O_{⋆}≃\frac{2O_{⋆}\gamma }{\gamma^{2}+\beta^{2}}\left[\frac{\gamma \eta \;\text{cos}\;\psi }{\beta }-\delta \right]-\frac{O_{⋆}\tau^{2}}{\gamma^{2}+\beta^{2}}$$

When γδ>0, anchor drift along the gain direction increases the apparent gain and reduces O (Section 5.7 treats the reversal). Residual-normal anchor drift enters Eq. (13) only through cosψ, so its first-order sign is set by the angle between $\hat{b}$ and $\vec{\eta }$; its positive-definite contribution is the second-order $\eta^{2}$, seen directly at β=0 in Eq. (14). The final term is the contribution from tangential displacement.

*(iii) Zero imposed novelty*. At β=0 the first-order form (13) diverges, since the η term carries 1/β, but the exact form does not:

(14) $$O=\frac{\eta^{2}}{(\gamma +\delta {)}^{2}+\eta^{2}+\tau^{2}}$$

With no imposed novelty, any measured O arises from the residual-normal component of anchor drift.

*(iv) A random novel direction carries no bias*. For $\hat{b}$ uniformly oriented in the residual normal space, E[cosψ]=0. The cross term in (12) therefore contributes variance but no first-order mean bias; what η leaves on average is the $\eta^{2}$ term, which appears as a floor in sweeps that randomize the imposed novel direction.

## 4 The cascade

The preceding sections establish the construction and introduce the associated limits in identifiability. Here, I establish which quantities can be interpreted and the conditions in which each may be estimated. Table 2 gives this dependency order which is validated in Section 5. The ordering is a dependency of the computation, not an ordering of damage: for example, over-estimating intrinsic dimension can bypass the intermediate gates and directly corrupt the gain axis (Section 5.2).

The results also differ in evidentiary status. Some are exact identities, including G+T+O=1 and the accounting in Proposition 1. Others are local asymptotic results, such as Propositions 2 and 3, whose validity depends on the stated sampling regime and approximation order. A third class comprises model-specific scalings, whose coefficients depend on the manifold and sampling distribution. The remainder are empirical results from the synthetic sweeps.

## 5 The gates

Validation is constrained by the same anchor-drift problem that limits the decomposition itself. Because the anchor is defined from the test point’s own neighbors, it moves to absorb any tangential component of the imposed displacement, so a naïve test would confound estimator error with the anchor-drift term of Eq. (8). Every construction below therefore either leaves neighbor selection invariant to the imposed displacement, or refers the anchor to the probe’s own projection; the two families and the manifolds each is used on are detailed in Supplement Section 1.2.

### 5.1 Gate 0: does a local chart exist?

**Figure 3.**
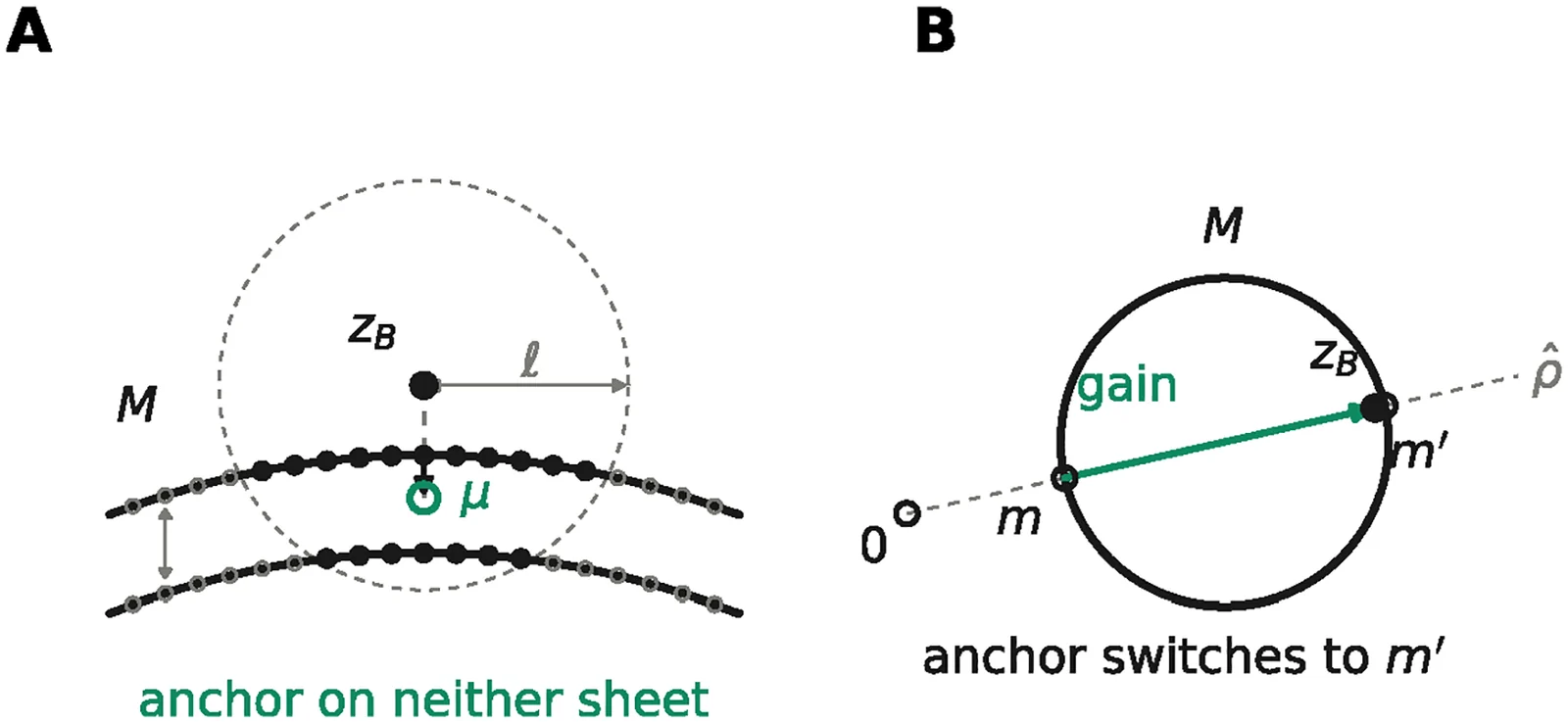
Gate 0: overlapping sheets and radial re-entry. **(A)** The anchor does not uniquely belong to one neighborhood when two sheets are within the radius ℓ. The anchor is the mean of points drawn from both and lands in the gap, on neither sheet. Damage is greatest when the separation is comparable to ℓ. **(B)** Radial re-entry. The ray from the origin meets M at m and again at m′. A large enough gain carries $z_{B}$ close enough to m′ that the nearest anchor switches, and the radial map is no longer injective.

When two sheets of a manifold lie within the neighborhood radius, the neighborhood mixes different local geometries, so the estimated tangent space belongs to neither sheet (Figure 3A). This can occur at a branch point or between nearby turns of a Swiss roll. The resulting artifact is largest when sheet separation is comparable to neighborhood extent, and it cannot be identified from G,T,O alone because it can mimic genuine gain or novelty (radial stacking reads as gain, ambient stacking as novelty; Table S2). Cross-sheet neighbor counts are likewise insufficient: the relevant quantity is the ratio of sheet separation to neighborhood extent (Table S2); under anisotropic noise the noise scale along the separation direction should also enter, which is not tested here. In practice, neighborhoods should therefore be chosen small enough that distinct sheets remain separated at the relevant noise scale.

Large gain can introduce a second failure: if radial scaling carries the test point into a region where the manifold intersects the same ray again, the gain representation is no longer injective and the nearest anchor can switch to another state (Figure 3B). Gain magnitude must therefore remain within a range over which the radial map is injective.

### 5.2 Gate 1: is d right?

**Figure 4.**
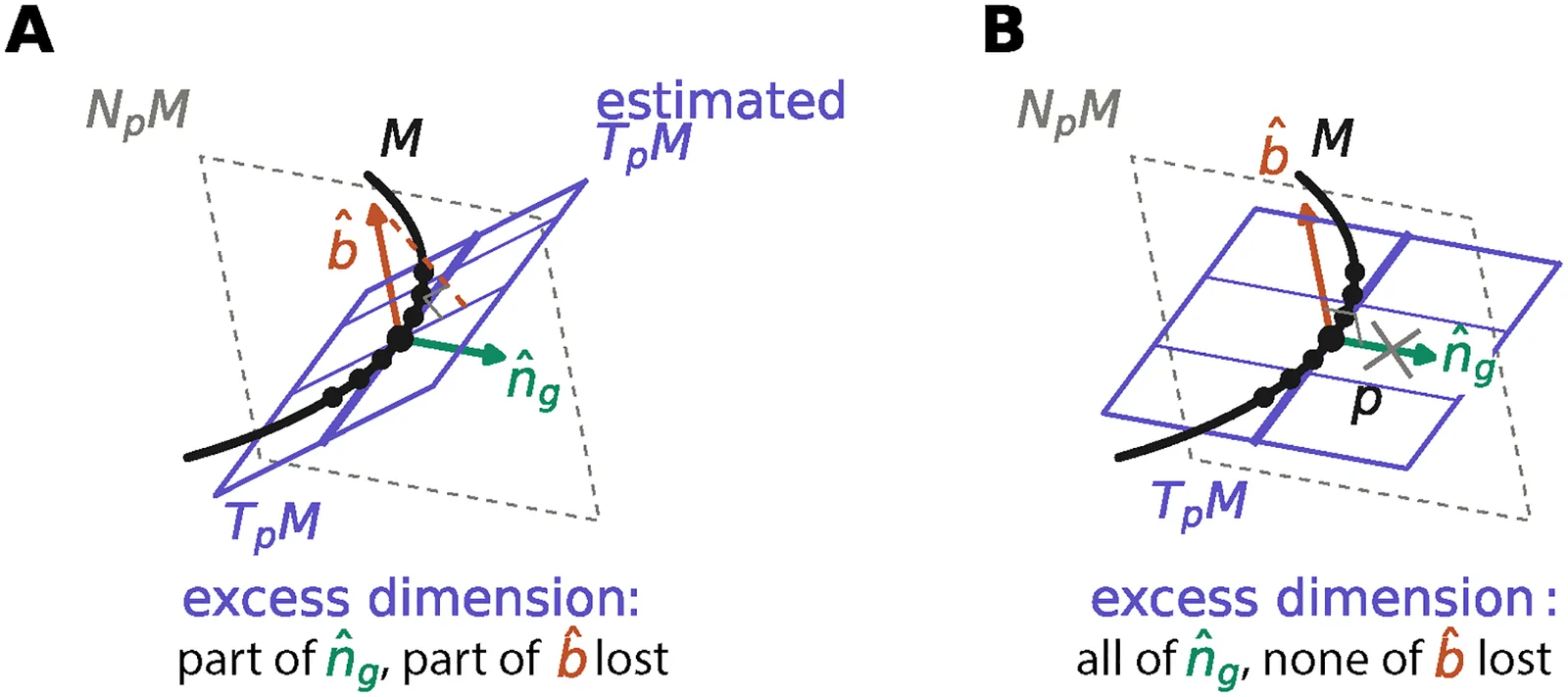
Gate 1: what an over-estimated dimension takes. **(A)** Over-estimating d makes the estimated tangent space a plane where the true tangent space is a line, taking one direction out of the normal space. A plane that does not perfectly align with ${\hat{n}}_{g}$ will absorb part of the gain and off-manifold space. **(B)** The same scene with the excess dimension rotated onto the gain axis. This scenario occurs when the variance around the sagitta exceeds the noise floor. When the tangent is perfectly aligned with ${\hat{n}}_{g},\overset{ˆ}{b}$ is perpendicular to the excess direction and nothing is lost, while the gain axis is absorbed into the tangent estimate and destroyed.

Assessing whether a vector lies off-manifold requires knowledge of the dimensionality of the manifold tangent space within an ambient space of dimension F. The off-manifold normal space is defined as the orthogonal complement of the assumed tangent space: over-estimating d removes directions from the space in which O and the gain axis are defined (Figure 4A). The expected survival law is obtained when the $q=d-d_{\text{true}}$ excess directions are uniformly oriented within the $F-d_{\text{true}}$-dimensional complement. Rotational invariance then gives a Beta distribution for the fraction of an off-manifold direction that survives the projection, with mean

(15) $$E[\text{survival}]=1-\frac{q}{F-d_{\text{true}}},$$

and its median is the appropriate comparator when survival is summarized by medians (Table S3; Supplement Section 1). We test this directly using a purely off-manifold displacement $\beta \hat{b}$, with $\hat{b}$ the unit residual-normal direction of Section 3.2. Define leakage as

(16) $$ℒ(\hat{b})={\left\|V_{d}^{⊤}\hat{b}\right\|}^{2},$$

the fraction of $\hat{b}$ incorrectly absorbed by the estimated tangent space. Leakage isolates the cost of dimension over-estimation.

The Beta prediction holds when the excess directions are effectively random (Table S3), but curvature creates an important exception. A curved manifold has a genuine normal direction associated with its local sagitta. If the variance that curvature contributes exceeds the noise floor, that specific direction has more variance than a generic ambient direction would, so it wins the $\left(d_{\text{true}}+1\right)$-th singular vector, and the excess dimension absorbs the normal direction and with it the gain axis (Figure 4B). At q=1 this produces two opposing effects: leakage is lower than the random-direction prediction because the discarded direction is not the imposed novelty direction, but the gain axis is destroyed, because that axis is constructed from the normal space. Consequently Gate 4 does not degrade smoothly with dimension over-estimation: its alignment can collapse at the first excess dimension and remain low thereafter.

When curvature is below the directional noise scale, the excess directions become effectively random and the Beta law is recovered, with alignment declining more gradually as the dimensional mismatch q increases. Table S3 locates the crossover by measuring the squared overlap between the local neighborhood’s $d_{\text{true}}+1$-th singular vector and the true normal direction — its *capture* of the excess dimension — which is nearly complete at low noise and falls to the chance level once the noise scale exceeds the curvature’s spread along the normal. On an origin-centered manifold the gain axis is the normal, so the alignment at q=1 satisfies $a^{2}\approx 1-\text{capture}$. Under anisotropic noise the relevant comparison is directional: whether the normal appears as the first excess direction depends on noise variance along the normal, not on overall noise magnitude. The expected loss of off-manifold energy is only part of the problem; depending on curvature and directional noise, the excess dimension can remove the normal direction itself and thereby invalidate the gain axis.

### 5.3 Gate 2: given a chart and the right d, is the local tangent estimate correct?

Next it is necessary to confirm whether the normal and tangent axes may be estimated. For each validation probe we decompose $r=z_{B}-\mu$ using both the estimated local geometry $\left(V_{d},{\hat{n}}_{g},\hat{b}\right)$ and the exact geometry of the generating manifold at the projection of μ onto it. Their difference isolates estimation error from anchor-driven effects.

**Figure 5.**
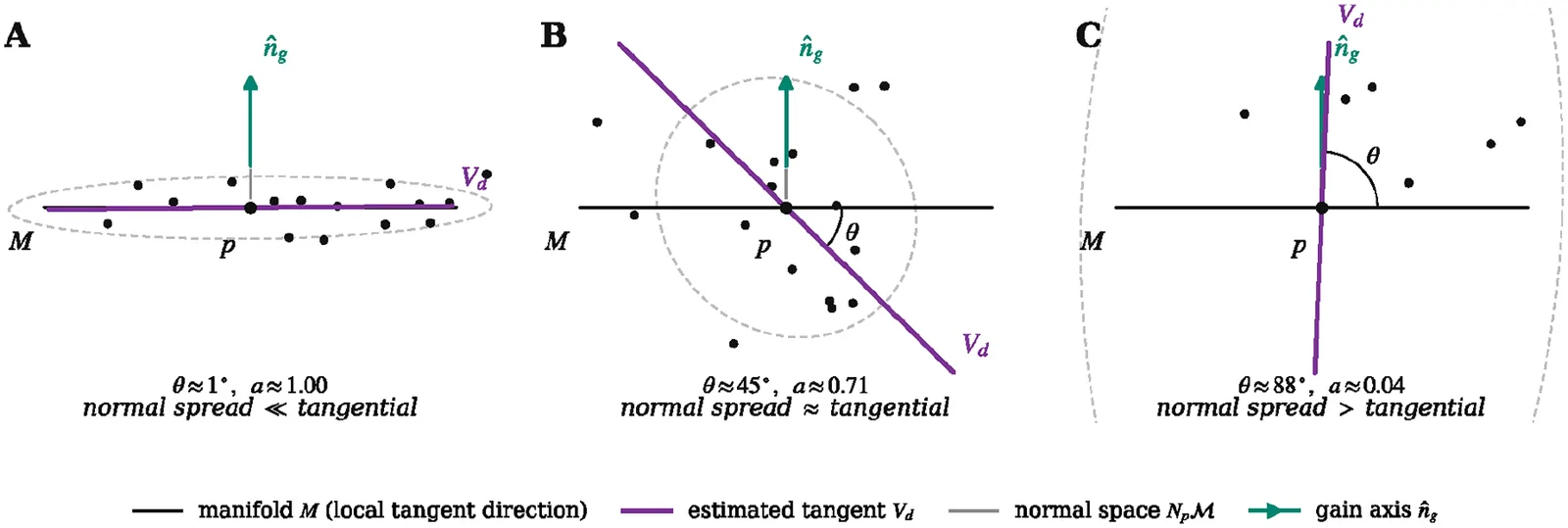
Gate 2, variance route: when normal-direction spread competes with tangential spread, the fitted plane rotates away from the true tangent and absorbs the gain axis. (A) Normal spread ≪ tangential spread: $V_{d}$ recovers the true tangent almost exactly (B) Normal spread ≈ tangential spread: the local covariance is close to isotropic, so the leading eigenvector is poorly determined; $V_{d}$ rotates by a large, effectively random angle, and roughly a third of the gain axis’s energy has migrated into the estimated tangent space (C) Normal spread > tangential spread: the leading eigenvector of the neighborhood is the normal direction. $V_{d}$ has rotated onto ${\hat{n}}_{g}$ and the gain axis is destroyed.

For the uniformly sampled 2-D sphere used here, the leading finite-sample error is a random tilt of the fitted plane (Figure 5). Under symmetric sampling the tilt has zero mean, but its variance is set by the sample covariance between tangential position and normal offset. For a two-dimensional neighborhood sampled uniformly, with normal curvature κ(κ=1/R on a sphere of radius R),

(17) $$E{\left\|V_{d}^{⊤}\hat{n}\right\|}^{2}=\frac{{↕}^{2}\kappa^{2}}{3k}[1+O(1/k)]$$

which on a uniformly sampled 2-sphere, where κ=1/R and ${↕}^{2}\approx 2kR^{2}/n_{\text{ref}}$, is $2/\left(3n_{\text{ref}}\right)[1+O(1/k)]$. Tables S4 and S5 confirm the prefactor, the $1/n_{\text{ref}}$ scaling and the independence from k and R, up to an O(1/k) excess that is largest at small k (derivation in the Supplement). The rotation is approximately exponentially distributed across probes, so its median lies below its mean.

#### 5.3.1 Two mechanisms by which the frame rotates

Splitting the local coordinates into a tangential part u and a normal part w,

(18) $$M=\left(\begin{array}{ll} E\left[uu^{⊤}\right] & E\left[uw^{⊤}\right]\\ E\left[wu^{⊤}\right] & E\left[ww^{⊤}\right] \end{array}\right),$$

the estimated tangent space can rotate through two mechanisms. Variance-driven rotation occurs when normal variance becomes large enough to compete with tangent variance, as with excess intrinsic dimension or anisotropic noise; this error does not vanish with additional reference data. Covariance-driven rotation occurs when $E\left[uw^{⊤}\right]\neq 0$, tilting the leading eigenspace even when normal variance is small.

Under symmetric sampling on a homogeneous manifold the covariance term vanishes in expectation, because the leading normal displacement is quadratic in tangent coordinates and therefore even in u. Finite sampling breaks this symmetry, producing the floor of Eq. (17). Systematic asymmetry, however, produces persistent covariance-driven rotation.

Locally, normal displacement is determined by curvature, and curvature gradients introduce higher-order terms that can break the symmetry between opposite tangent directions. Expanding the normal displacement along a single tangent direction gives a quadratic term set by the local normal curvature and a cubic term set by its derivative along that direction (derivation in the Supplemental proofs). The quadratic term is even in the tangent coordinate and so cannot covary with it; the cubic term is odd and can.

**Figure 6.**
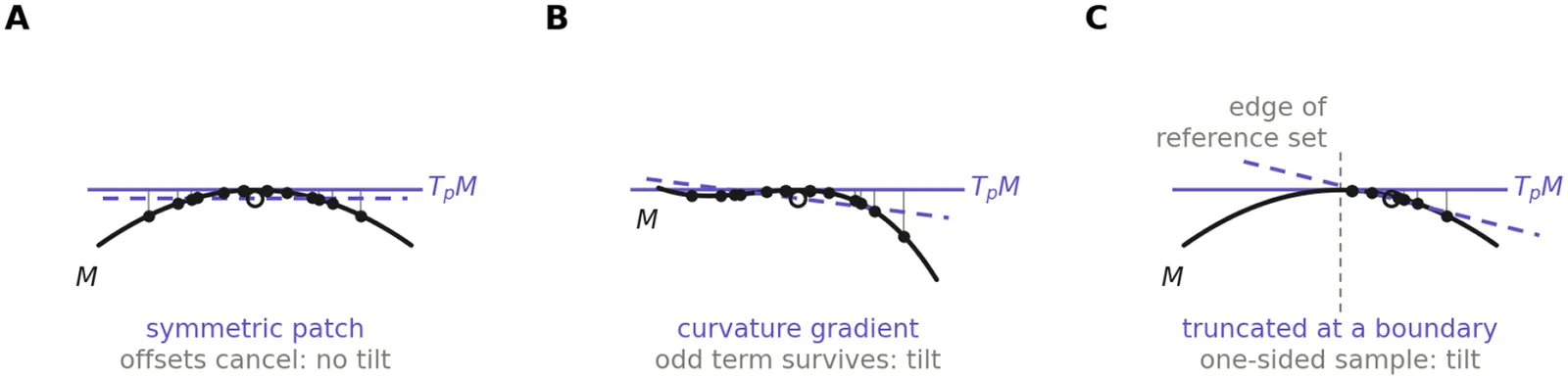
Gate 2, covariance route: what breaks the cancellation. **(A)** A symmetric patch. The quadratic sagitta is even in the tangential coordinate, so the normal offsets cancel in pairs and the fitted direction is parallel to the true tangent. Under symmetric sampling, curvature alone produces no tilt. **(B)** A curvature gradient adds an odd cubic term, the cancellation fails, and the fitted direction tilts. **(C)** The same symmetric curve as A, with the reference set truncated at its edge. The manifold is unchanged; only the sample is one-sided, and the fitted direction tilts again.

Beyond that floor, two forms of asymmetry break the symmetric case of Figure 6A and generate systematic covariance-driven rotation: a manifold whose curvature is not constant (Figure 6B), and a probe close to the edge of the sampled region (Figure 6C). In either case the symmetry argument that made the frame rotation k-independent no longer holds. Once the finite-sample floor is subtracted, the rotation grows as ${↕}^{4}\propto k^{2}$ in the presence of a curvature gradient, as predicted (Table S5). Near a boundary the rotation leaves the floor once the probe is within about one neighborhood extent of the edge, and grows close to ${↕}^{2}$ (Table S6). Therefore, large k influences bias through both anchor bias and, near an edge or a curvature gradient, through a frame tilt that grows faster than the anchor bias does. A density gradient produces a third route, although its scaling is not isolated here.

Neighborhood size therefore acts differently in symmetric and asymmetric regions. In symmetric interior regions, larger neighborhoods primarily increase anchor bias while leaving the frame stable. Near boundaries, or where sampling density changes, larger neighborhoods also increase frame rotation. Frame tilt and excess intrinsic dimension both rotate normal directions into $V_{d}$, and so both enter the leakage of Eq. (16); only an excess dimension also removes a direction from the complement, which is what the survival law of Eq. (15) counts.

### 5.4 Gate 3: where is the anchor?

Equation (8) isolates the anchor drift but leaves it unquantified. Because μ is a centroid of points sampled from a curved set, its displacement from the manifold has a leading term fixed by the local second-order geometry. Let I be the second fundamental form, the symmetric bilinear map $T_{p}ℳ\times T_{p}ℳ\to N_{p}ℳ$ whose value I(u,u) is twice the leading normal displacement of a point at tangent coordinate u; its component along a single normal direction is the local normal curvature used in Section 5.3.1. Let $\vec{H}=d^{-1}\sum_{i}I\left(e_{i},e_{i}\right)$ be the mean curvature vector, and let the k neighbors be drawn from a locally isotropic measure with ${↕}^{2}=E\|u{\|}^{2}$ the squared tangential extent of the neighborhood. Because the k-th neighbor lies on the boundary of the selection ball, ${↕}^{2}$ exceeds the areal value $\text{kArea}/\left(2\pi n_{\text{ref}}\right)$ by (k+1)/k; the centered estimator of Section 2.1 is unbiased for it.

#### Proposition 2 (anchor displacement).

With $p=\pi_{ℳ}\left(z_{B}\right)$ the manifold point the probe sits above, the expected bias in the anchor placement relative to that projected point can be described by,

(19) $$E[\mu ]-p=\frac{{↕}^{2}}{2}\vec{H}+O\left({↕}^{3}\right)$$

conditional on a neighborhood-selection distribution centered at p and locally isotropic in tangent coordinates.

#### Proposition 3 (anchor jitter).

The tangential fluctuation of the anchor has root-mean-square magnitude

(20) $${\left(E\|\overset{‾}{u}{\|}^{2}\right)}^{1/2}=↕/\sqrt{k}$$

**Figure 7.**
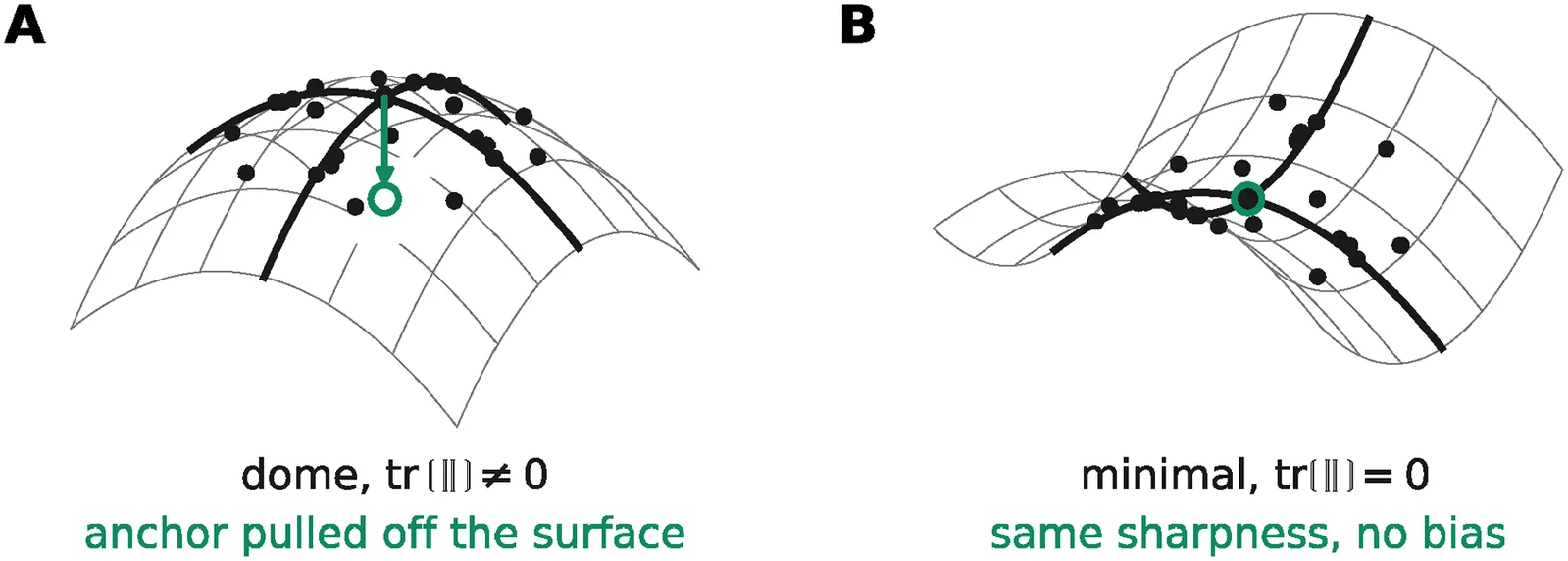
Gate 3: the trace, not the sharpness, biases the anchor. **(A)** A dome. Both principal curvatures share a sign, the trace of the second fundamental form I is non-zero, and the neighborhood mean is pulled off the surface along the normal. **(B)** A minimal surface with the same principal curvature magnitude and opposite signs. To leading order, the trace vanishes and the neighborhood mean remains on the surface. Sharp bending does not bias the anchor; the trace does.

Both are proved in the Supplement. The displacement is a normal-space vector, so at leading order the anchor is not biased along the manifold. It is governed by the *trace* of I rather than by curvature magnitude (Figure 7A), so at a point of zero mean curvature (for example a saddle with equal and opposite principal curvatures) the anchor is unbiased at leading order, however sharply the surface curves (Figure 7B). And its direction is that of $\vec{H}$, which need not be the gain axis — the topic of Gate 5. Enlarging the neighborhood increases the curvature bias, while its effect on anchor jitter depends on intrinsic dimension. Under locally uniform sampling $↕\propto k^{1/d}$, so the jitter scales as $↕/\sqrt{k}\propto k^{1/d-1/2}$. Thus, anchor jitter decreases in k only for d>2, k-invariant for d=2, and increasing for d=1.

For a sphere of radius $R,\vec{H}=-\hat{n}/R$ and the displacement is ${↕}^{2}/2R$ directed inward, independent of d and F; when the sphere is origin-centered this is collinear with the gain axis. With ↕ measured in the tangent plane the next order is known, $\delta =\left({↕}^{2}/2R\right)\left(1{+}^{2}/3R^{2}\right)$ (Supplement). Table S7 tests Propositions 2–3 on quadric patches, where the anisotropic, biaxial and zero-mean-curvature rows are the informative tests, and Table S8 repeats the test on a torus of revolution, where curvature varies over the surface and, for a sufficiently fat torus, the mean curvature changes sign — the measured displacement reverses with it, a directional test the sphere cannot supply.

#### 5.4.1 What the free parameters actually control

Proposition 2 expresses the anchor bias through neighborhood size ↕, which is not itself free but a consequence of several parameters that are. For a d-dimensional manifold of sampled volume A and $n_{\text{ref}}$ uniformly distributed reference points, k neighbors span a region of area $kA/n_{\text{ref}}$, so $↕\propto {\left(kA/n_{\text{ref}}\right)}^{1/d}$. For the 2-sphere of radius R this closes, to leading order in ↕/R and including the boundary factor (k+1)/k:

(21) $${↕}^{2}=\frac{2(k+1)R^{2}}{n_{\text{ref}}},\;\delta =\frac{{↕}^{2}}{2R}=\frac{(k+1)R}{n_{\text{ref}}},\;\frac{↕}{\sqrt{k}}=R\sqrt{\frac{2(k+1)}{k\;n_{\text{ref}}}}$$

On a closed sphere at fixed $n_{\text{ref}}$ and k the whole configuration scales with R, so δ∝R is dimensional: δ/R is fixed (Table S9). A flatter reference manifold is worse only relative to a fixed absolute displacement or noise scale; on a patch of fixed area, ↕ does not grow with R and δ falls. δ is reduced by raising $n_{\text{ref}}$ or lowering k. Bias and jitter respond differently to k and $n_{\text{ref}}$, though both scale with R: the bias grows as k while the jitter is k-invariant at d=2, and the bias falls as $n_{\text{ref}}^{-1}$ while the jitter falls only as $n_{\text{ref}}^{-1/2}$. At high sampling density the residual error is therefore dominated by fluctuation rather than by bias, although both vanish (Table S9).

Nearest-neighbor selection favors points whose noise displaced them toward the probe; under isotropic noise that preference has no net direction and cancels, but under anisotropic noise it does not, and Proposition 2 fails in both directions depending on where the anisotropy sits (Table S10). Noise along the radial direction erases the sagitta, since a radially thickened shell allows selection to pull the neighbors’ mean radius toward the probe’s own; noise concentrated tangentially inflates it, because tangentially displaced points from further around the manifold become selectable. Noise in the residual normal space is inert. Two further channels do not act here: a probe’s own displacement cannot move the anchor within the validation construction, by design (Section 3.1), so its absence is not evidence about other constructions; and under the isotropic reference-noise model tested here, reference noise enters as fluctuation and by inflating the *measured* ↕ rather than as a bias channel of its own (Table S9).

### 5.5 Gate 4: does the gain axis exist?

The alignment $a=\left\|P_{N}\hat{\rho }\right\|$ governs this gate and, unlike Gate 5, is computable from μ and $V_{d}$ alone. The validation manifolds are origin-centered spheres, where the radial direction is exactly the normal and this gate holds by construction; the complementary regime is a cone through the origin, where the alignment is zero in the continuum and small under finite sampling. A torus of revolution provides the intermediate case: its alignment has a closed form varying over the surface and passing through zero, so a single manifold contains well-conditioned and degenerate regions at once. The estimated alignment tracks the analytic value pointwise and degeneracy is flagged only near the zero crossing (Table S8).

#### 5.5.1 Anisotropic noise

Anisotropy in a tangential direction moves the fractions by at most a few hundredths even when the noise scale along it is about twice the neighborhood extent (Table S11): the anchor tracks tangential displacement, and the same tracking that makes T unidentifiable absorbs tangential noise. Anisotropy in the residual normal space is likewise almost inert, entering numerator and denominator of O together. Anisotropy along the gain axis is not absorbed. There the alignment degrades and energy moves out of G and into O, monotonically in the ratio of the noise scale along that direction to the neighborhood extent and with no safe threshold (Table S11); the mechanism is the variance route of Section 5.3.1, and the frame-rotation quantity of Eq. (17) rises by orders of magnitude in step with the alignment’s fall (Table S12).

The damage is done while the alignment is still far above any tolerance one would set: by the time a would trip a threshold chosen to flag a non-existent gain axis, most of G has been converted to O. The tolerance detects a gain axis that does not exist; it does not detect one that exists but has been contaminated. Report a alongside G and O and treat decreases in a as places where the G/O split becomes ambiguous.

### 5.6 Gate 5: is anchor displacement in the gain axis or residual normal space?

**Figure 8.**
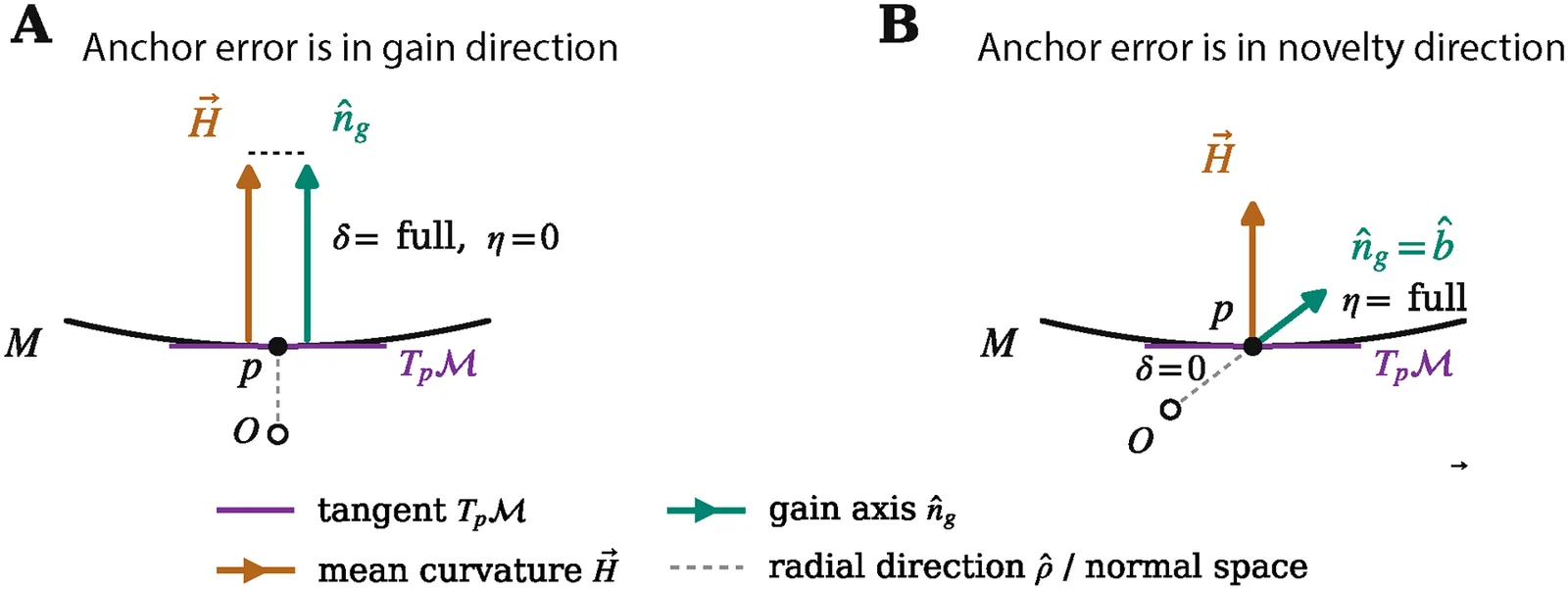
Gate 5: whether anchor-displacement bias is booked as gain or as novelty depends on where the origin sits relative to the curve’s osculating plane. (A) The origin O lies on the normal line through p, so the radial direction $\hat{\rho }=(p-O)/\|p-O\|$ has no tangential component and ${\hat{n}}_{g}=\hat{\rho }$ is parallel to $\vec{H}:|c|=1$, and the anchor’s displacement is absorbed entirely into δ, read as gain. (B) The origin leaves the plane the curve locally bends in (the osculating plane spanned by the tangent and $\vec{H}$), displaced instead along the binormal $\hat{b}$; then ${\hat{n}}_{g}=\hat{b}$ is orthogonal to $\vec{H}:c=0$, and the identical displacement is absorbed entirely into η, read as novelty.

Decomposing the anchor displacement of Proposition 2 into a component along the gain axis and a component in the residual normal space,

(22) $$\delta =-\frac{{↕}^{2}}{2}{\vec{H}}^{⊤}{\hat{n}}_{g},\;\eta =\frac{{↕}^{2}}{2}\left\|P_{\text{res}}\vec{H}\right\|,$$

shows that it enters the denominator of O as apparent gain through δ and the numerator as spurious novelty through η. Only the first is present when manifold curvature aligns with gain, $\vec{H}\|{\hat{n}}_{g}$. The governing quantity is therefore a second alignment,

(23) $$c={\hat{n}}_{g}^{⊤}\vec{H}/\|\vec{H}\|,$$

independent of the a of Gate 4: at |c|=1 the displacement is absorbed entirely into the gain leg and the numerator of O is unaffected (Figure 8A), at c=0 it appears entirely as spurious novelty (Figure 8B), and in general it is apportioned as $c^{2}$ and $1-c^{2}$. Unlike a,c requires an estimate of I and must be argued from the manifold geometry rather than read off the data.

Table S13 confirms the case |c|=1 using pure geodesic probes on spheres. The absolute off-manifold energy there is flat at the observation-noise floor across every radius and neighborhood size tested, while the fraction O varies from cell to cell through its denominator alone — the sagitta and the tangential jitter together — not through leakage into the numerator. This is the practical payoff of building the gain axis inside the normal space: the sagitta is routed into G and the numerator of O is left untouched, whenever |c|=1.

That case is not general, and there is a structural reason it is not merely a property of the sphere. For a d-manifold whose linear *span* is (d+1)-dimensional the normal space inside that span is one-dimensional, so $P_{N}\hat{\rho }$, the surface normal and $\vec{H}$ are forced parallel and |c|=1 identically — which covers the sphere, the spherical cap, every quadric patch in three-space, the torus of revolution and the ellipsoid. Reaching |c|<1 requires the manifold’s linear span to be at least d+2. The Clifford torus in four dimensions is the simplest such geometry with alignment exactly one, so it isolates this gate from Gate 4; Table S14 shows the absolute novel energy rising from the noise floor to track η as the torus is made asymmetric, and growing in proportion to ${↕}^{2}$ where on the sphere it was flat. Because that torus has a parallel second fundamental form (the rate of change of the curvature is zero across the surface), a circle × ellipse is used to repeat the test where the second fundamental form varies (Table S15); the rule survives, with both regimes appearing on a single manifold.

### 5.7 Gate 6: is the ratio well-conditioned?

O is defined as a ratio (energy along one direction over total energy), and any ratio estimate is only trustworthy when its denominator is not itself dominated by noise or fluctuation. On an origin-centered sphere η=0, and the first-order expansion (13) reduces to

(24) $$E\left[O\right]-O_{⋆}≃-2O_{⋆}\sqrt{1-O_{⋆}}\frac{\delta }{\sqrt{\gamma^{2}+\beta^{2}}}sgn\left(\gamma \right)-\frac{O_{⋆}\tau^{2}}{\gamma^{2}+\beta^{2}}.$$

The first term is *signed by the direction of gain*: for a manifold that curves toward the origin (${\vec{H}}^{⊤}{\hat{n}}_{g}<0$, as on an origin-centered sphere) an amplifying change has O under-reported and a suppressive change has it over-reported, so the off-manifold fraction is a conservative novelty statistic only for gain increases. The second term is set by the jitter rather than by curvature. At fixed k both terms fall together as $n_{\text{ref}}^{-1}$; they separate in k, where δ grows while $\tau^{2}$ is k-invariant at d=2, and the tangential term matters most where $\tau^{2}/\delta$ is largest (Table S16).

Because O is a ratio of quadratic forms rather than a ratio of expectations, equating the two requires the denominator’s fluctuation to be small relative to its mean. With $\tau^{2}\sim {↕}^{2}/k$ by Proposition 3, a sufficient leading-order condition is $(\gamma +\delta {)}^{2}+\beta^{2}+\eta^{2}\gg {↕}^{2}/k$. Its left side is not observable, but $\|r{\|}^{2}$ is: by Eq. (11) and Proposition 3, $E\|r{\|}^{2}=(\gamma +\delta {)}^{2}+\beta^{2}+\eta^{2}+{↕}^{2}/k$, so the condition is equivalent to

(25) $$C_{6}=\frac{k\|r{\|}^{2}}{{↕}^{2}},\;C_{6}-1\gg 1$$

**Figure 9.**
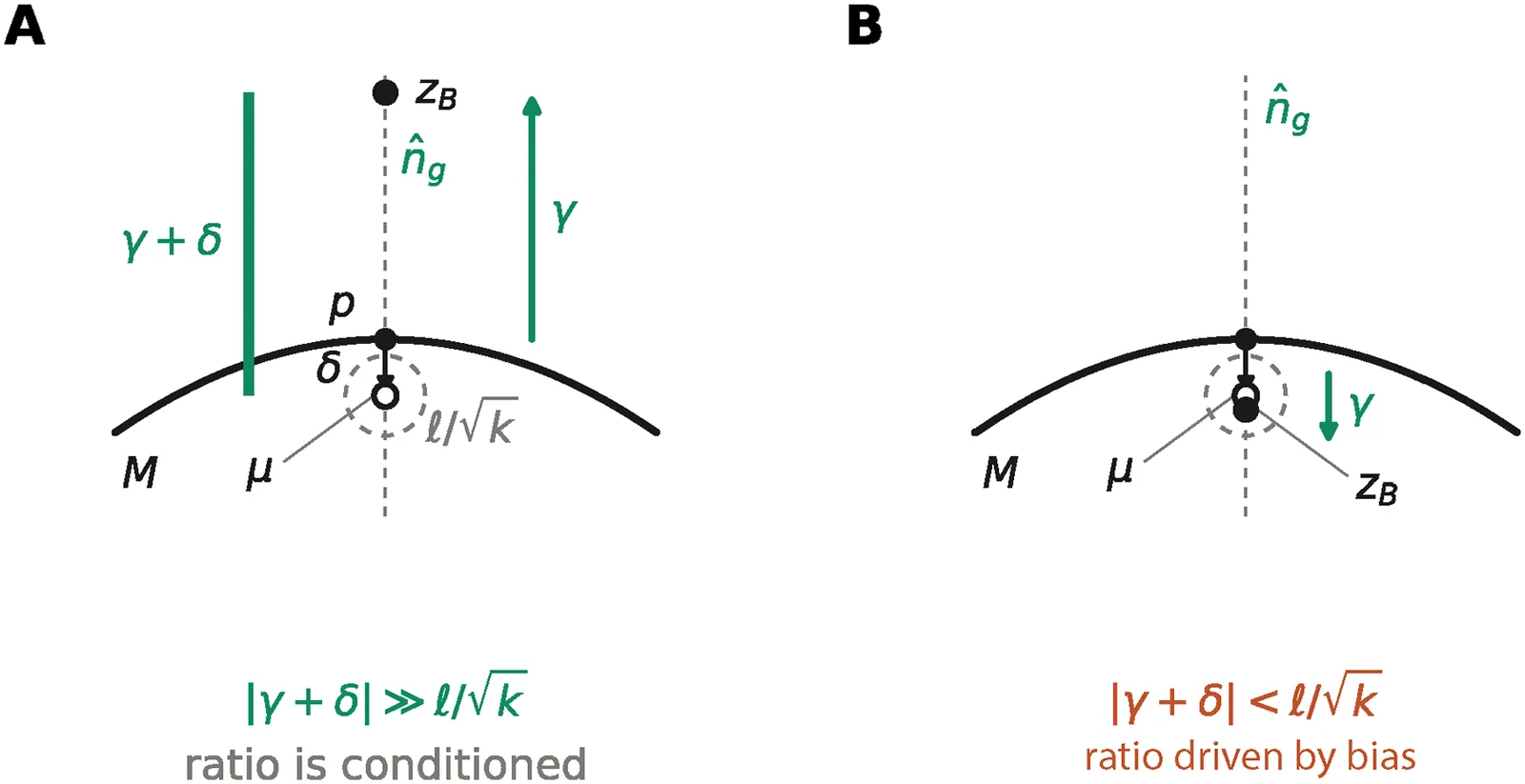
Gate 6: the recovered off-manifold fraction is only as reliable as the ratio $(\gamma +\delta {)}^{2}+\beta^{2}+\eta^{2}\gg {↕}^{2}/\text{k}$ is large, an observable condition captured by ${\text{C}}_{6}=\text{k}\|\text{r}{\|}^{2}/{↕}^{2}$. (A) An amplifying change: γ and δ add, so the resolved leg is long compared with the jitter disc and $C_{6}\gg 1$ — the ratio is conditioned. (B) A suppressive change on the same manifold, with the same anchor and the same δ:γ and δ nearly cancel, the resolved leg falls inside the jitter disc, and $C_{6}\approx 1$ — the leading term of the ratio is a fluctuation rather than a measurement.

This fails where a suppressive change is nearly cancelled by the anchor displacement, and there the leading denominator term is itself a fluctuation (Figure 9). Under anisotropic noise $\tau^{2}$ changes, but it tracks the ${↕}^{2}/k$ measured on the same neighborhoods, so the check remains computable provided ↕ is measured rather than assumed (Table S12). $C_{6}$ is computed for each probe from its own ‖r‖ and the ↕ of its neighborhood, so this is one of the two gates a reader can act on directly.

Table S17 gives the recovered off-manifold fraction against the imposed value across neighborhood size, imposed magnitude and the sign of gain. The predicted sign reversal is observed, and the error of the closed form grows as $C_{6}$ falls (Table S17 bins it by $C_{6}$). Neighborhood size has no favorable direction here: the jitter that would improve with larger k is invariant at d=2, while the bias grows regardless.

## 6 What the validation supports

On a manifold where the gain axis is defined and the mean curvature vector is aligned with it, the decomposition recovers the off-manifold share of a change closely wherever the conditioning of Gate 6 holds. Biases in this off-manifold estimate can be understood by the geometry of the local neighborhood: signed by the direction of gain, linear in the anchor displacement at first order, and computable up to the second fundamental form.

The cascade defines the scope of the decomposition, and the seven gates are not equally available to the analyst. Gates 4 and 6 are computable from the decomposition directly, through a and $C_{6}$. Gate 2’s bound is conditional on Gate 1 and on isotropic noise. The covariance route of Section 5.3.1 means boundary proximity and curvature gradients remain a per-manifold check. Gates 3 and 5 depend on the mean curvature vector and its alignment with the gain axis; neither is estimated here, so both must be argued from manifold geometry. Gate 5’s booking rule is validated over a range of c, but the rule tells a reader how the displacement is apportioned given c, not what c is for their manifold. Gates 0 and 1 are upstream of everything and produce artifacts shaped like real results.

### 6.1 Practical interpretation and diagnostics

Taken together, the results support a cautious interpretation of gain, reconfiguration, and novelty. The decomposition is most informative when the reference manifold has a locally valid chart, the intrinsic dimension is supported by the data, the tangent estimate is stable across reasonable neighborhood scales, the test state lies within a plausible projection region, and the radial axis is sufficiently aligned with the hypothesized gain direction. Ratio-based summaries require an additional conditioning check because small total or reference-aligned components can make relative attribution unstable even when the underlying vector decomposition is well behaved. In general, there is a trade-off between enforcing large reference manifold sample size and reducing neighborhood size. For noise along the gain axis the damage is governed by the noise scale along $\hat{\rho }$ relative to the neighborhood extent, $\sigma_{w}/↕$ (Table S11, where $\sigma_{w}$ is varied at fixed ↕). A larger neighborhood should therefore reduce it, the opposite of Gates 2 and 3, where larger neighborhoods increase anchor bias and, near boundaries or curvature gradients, frame rotation. Where common-mode fluctuation dominates, that trade-off should be made deliberately rather than by defaulting to small k.

Several tools are available for estimating intrinsic dimensionality^11–13^, though neural data provide several reasons to treat these estimates cautiously. In simulations and recordings, linear methods can overestimate the dimension of nonlinear manifolds, noise can create spurious dimensions, and finite sample size can lead to underestimation when the intrinsic dimension is large^14^. Mathematical analyses reach the same conclusion from a different direction: tangent and dimension estimates are reliable only when neighborhood scale, sampling density, reach, and noise are jointly compatible^15,16^. An apparently stable estimate of dimension is therefore not sufficient evidence that the resulting normal space is correctly specified. Over-estimation biases the off-manifold fraction and, on a manifold whose curvature exceeds its noise, collapses the alignment of Gate 4 outright. Underestimation, not simulated here, leaves off-manifold directions in the complement but places a true tangent direction there too, so tangential displacement and jitter are booked as O and the gain axis acquires a tangential component. The alignment should therefore be examined as d varies: a sharp drop between d and d+1 indicates that the normal direction is being absorbed; on an origin-centered manifold the drop reads the absorbed share directly, since $a^{2}\approx 1-\text{capture}$ (Table S3).

Noise geometry matters as well. Isotropic noise may mainly increase the variance of the reported components, whereas anisotropic or state-dependent noise can rotate the estimated tangent space, distort the radial direction, and create systematic normal displacement. The local-PCA literature explicitly treats non-uniform distributions and varying noise as sources of perturbation rather than as harmless residuals^16^. The decomposition is robust to anisotropy in a tangential direction because the anchor tracks it (Table S11); this matters because correlated variability in population recordings lies largely within the signal manifold. Anisotropy along the gain axis is not absorbed, however. In real-world population recordings anisotropic noise is often driven by shared multiplicative fluctuation. This is a radial displacement, and on an origin-centered spherical manifold the radial direction is the gain axis, so shared multiplicative fluctuation is exactly noise concentrated on ${\hat{n}}_{g}$ (Section 5.5.1). Whitening is not a neutral remedy. Centering, as in z-scoring, moves the origin, which the construction requires to be zero population activity. A purely linear rescaling $\Sigma^{-1/2}$ keeps the origin and maps rays to rays, so multiplicative gain stays radial, but it replaces the inner product: $P_{N},a,c$ and the split between G and O are then defined in a different metric, and the decomposition answers a different question. In practical applications, noise should be assessed relative to the local signal geometry, not only by its total magnitude.

In an ideal case, the mean curvature is small on the scale of the neighborhood, or the radial direction is close to the mean curvature direction, or the analysis is confined to a regime where the displacement is large against ${↕}^{2}\|\vec{H}\|/2$. An analysis declaring none of these reports O with a bias of known form but unknown size: its δ part flips sign with the gain, and where |c|<1 its $\eta^{2}$ part raises O regardless of the sign of the gain.

A reported decomposition should therefore be accompanied by diagnostics for:

- local neighborhood stability and estimated tangent-space rotation;
- sensitivity to intrinsic-dimension choice;
- distance to the presumed tubular region and proximity to boundaries or bottlenecks;
- radial alignment and radial injectivity over the relevant reference neighborhood;
- estimated anchor drift under resampling and under the hypothesized transformation;
- anisotropy and scale of measurement noise; and
- conditioning of the ratios ($C_{6}$, Eq. 25).

Analyses that compare sets of test points against a constant manifold hold the biases of the reference geometry fixed, such that a difference in G, T, or O across probe sets is interpretable so long as the sets draw anchors from a comparable distribution over the manifold and have comparable gain sign and magnitude: by Eq. (24) the bias depends on both, so two sets with the same anchors but opposite gain acquire opposite biases (Table S17).

## 7 Discussion

By decomposing a neural state into tangential reconfiguration, radial gain, and residual off-manifold displacement, I provide a geometric framework for asking whether a change is consistent with amplification of an existing representation or departure from the sampled repertoire. The cascade of identifiability gates developed here shows that errors in the reference manifold, intrinsic dimensionality, tangent estimation, gain-axis alignment, and ratio conditioning can systematically transform one type of change into another. Thus, the principal contribution is not simply a new measure of neural novelty or gain, but a framework for determining when such mechanistic attributions are interpretable and when the geometry of the measurement itself makes neural state changes inherently ambiguous.

### 7.1 Neural distance compared to what?

The use of a local anchor places the method within the broader theory of geometric inference from sampled manifolds. For a continuous manifold, positive reach defines a tubular neighborhood in which the nearest-point projection is unique. Reach also relates to how rapidly tangent spaces can vary and captures both high curvature and near self-approaches that create projection ambiguity^17^. These results provide a natural geometric condition for treating a test state as a perturbation of a particular local reference point.

The empirical anchor used here is not, however, the exact projection onto the continuous manifold. It is a local statistic computed from a finite and potentially non-uniform sample. Even when the underlying manifold has positive reach, a local centroid can be displaced by curvature, boundaries, density gradients, and sampling variability. Local tangent estimation faces the same trade-off: small neighborhoods reduce curvature bias but provide little averaging, whereas large neighborhoods improve sampling stability while mixing regions with different tangent spaces^15,18^. Bounds for local PCA likewise depend on reach, density, neighborhood radius, and the geometry of the noise distribution^16^. Thus, increasing the number of reference samples does not by itself remove all anchor error. It can reduce sampling variance while leaving systematic displacement caused by the chosen neighborhood, with systematic distortions due to curvature, for example.

The most important limitation is the difference between estimating a decomposition relative to a selected anchor and recovering the transformation that generated the test state. Given an estimated reference manifold and a test point, the method can compute tangential, gain-axis, and residual components relative to that geometry. What it cannot generally recover from those observations alone is the unobserved starting state, the amount of anchor drift, or the causal sequence of transformations that produced the test point. A state displaced along the gain axis could reflect true gain, a change in the reference state, a deformation of the manifold, or a combination of these effects. The distinction parallels a broader warning in neural-manifold research: manifold geometry is a useful descriptive summary, but it does not by itself identify the circuit mechanism that generated that geometry or its dynamics^19^.

Correspondence across conditions or sessions can aid in identifying which state should serve as the reference, making it possible to distinguish global scale, rotation, and residual deformation. Low-dimensional alignment methods use stable neural channels or shared structure to resolve coordinate ambiguity between sessions^20,21^. Behavioral variables can serve a similar role by anchoring latent states to common task conditions and improving consistency across recordings^22^. Alignment of latent dynamics extends this idea across recording sessions by using the rules governing state evolution as a reference, rather than matching static point clouds alone^23–25^.

Even when correspondence may be motivated through estimation of the initial point of displacement, many of the biases presented here are still relevant. A starting location resolves the anchor-location ambiguity of Gate 3 and removes the associated anchor-drift contribution to Gate 5, but it does not by itself resolve Gate 0 (local chart validity), Gate 1 (intrinsic dimension), Gate 2 (tangent estimation), Gate 4 (radial-axis alignment), or Gate 6 (ratio conditioning).

### 7.2 The radial axis is a coding assumption

The further partition of the normal space into a radial gain direction and a residual novelty direction introduces a modeling assumption: a radial axis is appropriate when multiplicative modulation acts on the population response vector relative to a meaningful origin. Shared cortical variability has been modeled using multiplicative gain together with additive offsets, and the two components have different effects on population covariance and stimulus discriminability^26^. Related work has shown that multiplicative modulation can account for systematic response variability and can be distinguished from private spike-generation noise^27^, while different neurons can express different mixtures of multiplicative and additive modulation^26,28,29^. The radial construction should therefore be presented as a coding hypothesis: it tests whether a privileged direction in the observed population coordinates behaves like gain.

Radial injectivity is consequently an identifiability condition for this particular parameterization. If distinct reference states lie on the same ray from the origin, radial position cannot uniquely identify the underlying state. In that setting, a radial displacement may be compatible with multiple combinations of state change and gain. The condition is best viewed as a prerequisite for interpretation rather than as a generic property of neural manifolds. Chung, Lee, and Sompolinsky (2018) showed that neural manifolds possess meaningful radial geometry, including characteristic manifold radii and directions associated with their extent in activity space^30^. Their framework therefore provides an important geometric foundation for treating radial structure as a property of neural representations. Here, however, I identify a different consequence of radial geometry: radial scaling can alter the empirical reference anchor itself. When the radial direction has a tangential component relative to the manifold, multiplying a state by a gain factor moves the state through the ambient space and creates ambiguity as to which is the state that is being gain-modulated. In this sense, the present analysis extends the geometric perspective of Chung et al. from describing the structure of neural manifolds to asking whether that structure permits a uniquely and stably identifiable decomposition of transformed states.

**Figure 10.**
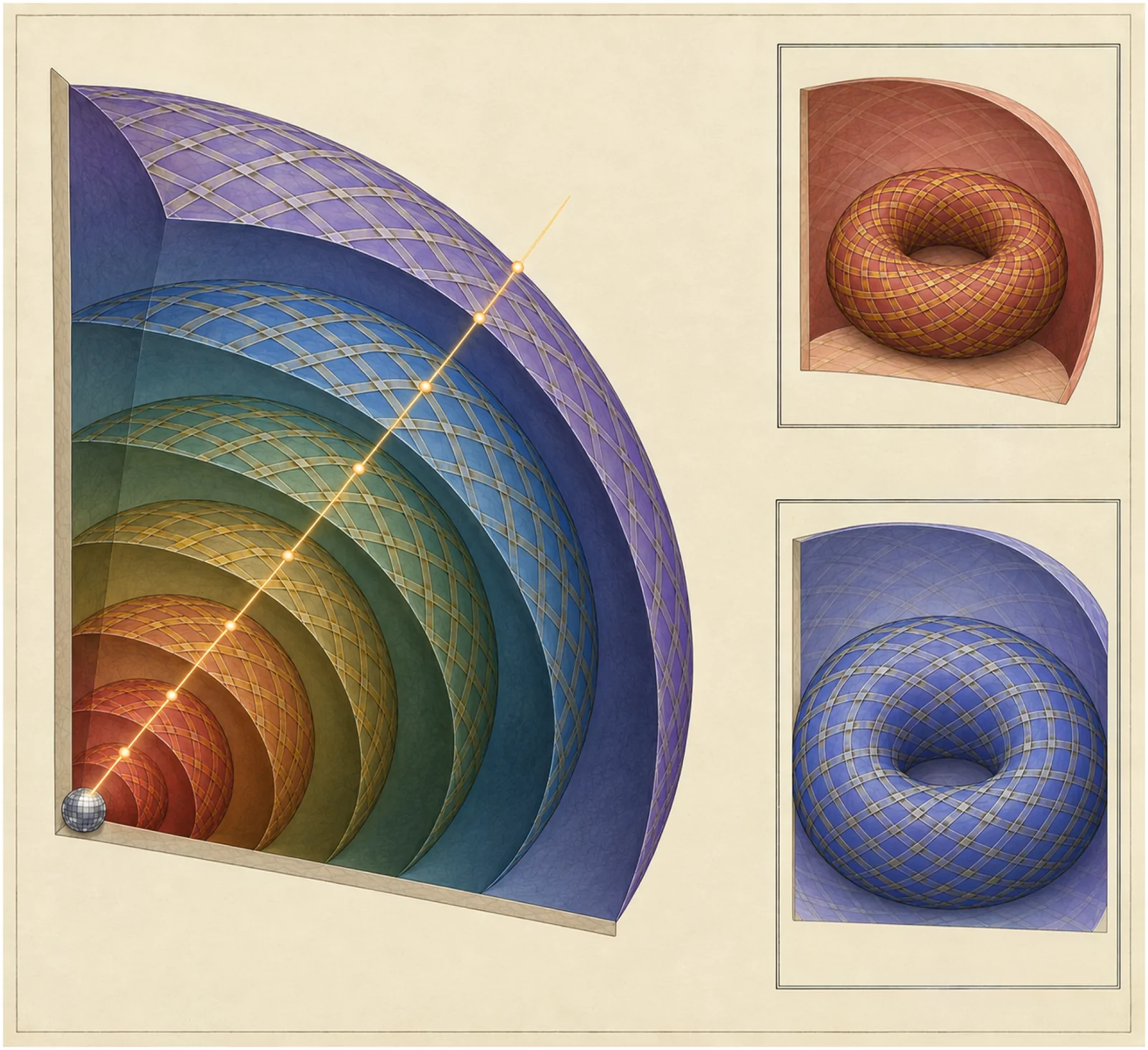
Embedding of a 2-D toroidal manifold on a hypersphere at multiple gain intensities. Radial gain moves the coded state between concentric tori embedded at constant radius from the origin in ambient state space (main panel; ray marks a trajectory of increasing gain), as in a Clifford torus embedded in a higher-dimensional sphere. The insets isolate the smallest (red) and largest (blue) tori from this family, shown in the torus’s conventional $R^{3}$ embedding for visualization: the intrinsic angular coordinates $\theta_{1},\theta_{2}$ are unchanged, while the embedding radii $R_{1},R_{2}$ scale with gain, so a fixed angular separation corresponds to a larger physical distance at higher gain.

This decomposition also offers a normative argument that situations that require robust state disambiguation in the face of intense gain fluctuations should be mapped onto a spherical manifold in ambient state space (more precisely, the positive orthant, since rates cannot be negative), while situations in which gain defines the manifold coordinate should tend towards a cone. Spheres in ambient state space are compatible with familiar low-D neuronal manifolds. For example, the coding manifold for a grid cell module is modeled as a two-dimensional torus^31^. For the gain axis to be both radial from a true zero-activity origin and normal to the toroidal surface constrains its embedding such that the torus lies at constant radius from the origin (exactly so if a=1 everywhere), as in a Clifford torus embedded in a higher-dimensional sphere. Radial gain then moves the representation between concentric tori (Figure 10). Although the toroidal coordinates are preserved, this scaling changes the metric of the embedded torus: distances between states, and so the discriminability of nearby positions for a readout with fixed noise, scale with gain. Grid fields expand in novel environments^32^, though the relationship between gain and grid-field spacing, and more generally how representational geometry is constrained by the need to disambiguate tangential from gain-related displacements, remain empirical questions.

### 7.3 Novel from whose point of view?

The decomposition is defined with reference to a sampled manifold and the Euclidean geometry of the recording space, but neither is necessarily what a downstream neural observer sees^33^. A linear readout of ensemble activity will impose its own projection, with a null space in which some displacements are invisible regardless of their magnitude in neuronal space. This change in reference frame alters the interpretation of the decomposition in two important ways. First, for multiplicative gain, the relevant zero is not necessarily a single point at the origin, but the set of activity patterns that produce no output in the readout. Second, the reader-centric view separates two forms of activity that the present geometric construction treats alike as off-manifold: activity that falls within the readout’s null space has no downstream consequence, whereas activity that is visible to the readout but lies outside the manifold represents a state the population does not normally produce^34^. The former is therefore not novelty; the latter may be. Both appear as off-manifold activity in the present framework, placing a fundamental limit on what O can mean. More generally, this highlights that novelty is not an intrinsic property of a neural representation alone, but a property of the relationship between a representation and the system that reads it.

## Supplementary Material

1

## Figures and Tables

**Table 1.** Symbols used throughout.

Symbol	Meaning	First used
**Ambient space and manifold**		
F	dimension of the ambient space, $R^{\wedge }\text{F}$	§2.1
ℳ	reference manifold	§2.1
$Z_{A}$	reference sample, a point set in $R^{\wedge }\text{F}$ drawn from ℳ	§2.1
p	a point on the reference manifold	§2.1
$z_{B}$	test point	§2.1
d	assumed tangent dimension used by the estimator	§2.1
$d_{\text{true}}$	true intrinsic dimension of the manifold	§5.2
**Local geometry at a point**		
$T_{p}ℳ$	tangent space at p	Eq. (1)
$N_{p}ℳ$	normal space at p	Eq. (1)
$P_{T}(\text{p})$	projector onto ${\text{T}}_{p}ℳ$	Eq. (2)
$P_{N}(\text{p})$	projector onto ${\text{N}}_{p}ℳ,\text{I}-{\text{P}}_{t}(\text{p})$	Eq. (2)
I	second fundamental form, the symmetric bilinear map ${\text{T}}_{p}ℳ\times {\text{T}}_{p}ℳ\to {\text{N}}_{p}ℳ$	§5.4
$\vec{H}$	mean curvature vector	§5.4
**Radial gain axis**		
$\overset{ˆ}{\rho }(\text{p})$	unit radial direction at p, p/∥p∥	Eq. (3)
a(p)	alignment, $\left\|{\text{P}}_{N}(\text{p})\overset{ˆ}{\rho }(\text{p})\right\|\in [0,1]$	Eq. (3)
${\overset{ˆ}{n}}_{g}(\text{p})$	gain axis: the normalized radial direction with its tangential part removed	Eq. (5)
$N_{\text{res}}(\text{p})$	residual normal space: the orthogonal complement of the gain axis within ${\text{N}}_{p}ℳ$	Eq. (4)
$P_{\text{res}}(\text{p})$	projector onto ${\text{N}}_{\text{res}}(\text{p})$	Eq. (6)
**Displacement and energy fractions**		
$r_{p}$	displacement from the reference point, ${\text{z}}_{B}-\text{p}$	§2.1
G,T,O	gain, on-manifold (tangential), and off-manifold energy fractions; G + T + O ≡ 1	Eq. (7)
$r_{\text{tan}}$	on-manifold (tangential) component of ${\text{r}}_{p}$	Eq. (6)
$r_{\text{novel}}$	off-manifold component of ${\text{r}}_{p}$	Eq. (6)
**Estimator (data-based substitutes)**		
$N_{A}$	local neighborhood: the k nearest reference points to ${\text{z}}_{B}$	§2.1
k	number of nearest neighbors defining the local neighborhood	§2.1
$n_{\text{ref}}$	number of reference points	§5.4.2
μ	anchor: centroid of N_A	§2.1
$X_{c}$	mean-centered neighbor matrix, N_A -μ	§2.1
$V_{d}$	estimated tangent basis: leading d right singular vectors of X_c	§2.1
${\overset{ˆ}{\rho }}_{\mu }$	estimated radial direction, μ/‖μ‖	§2.1
ℓ	tangential extent of the local neighborhood	§5.4
r	displacement from the anchor, ${\text{z}}_{B}-\mu$	§3
**Identifiability and anchor drift**		
m	true manifold state from which the change proceeded	§3
λ	gain magnitude, ${\text{z}}_{B}\approx \lambda \text{m}$	§3
**Anchor-frame decomposition (Proposition 1)**		
δ	signed anchor displacement along the gain direction, ${\overset{ˆ}{n}}_{\text{g}}⊤(\text{m}-\mu )$	Eq. (10)
$\vec{\eta },\eta$	residual-normal component of the anchor displacement, and its magnitude	Eq. (10)
$\tau^{2}$	squared tangential component of the anchor displacement	Eq. (10)
γ	imposed displacement along the gain direction	§3.2
β	imposed residual-normal displacement	§3.2
$\overset{ˆ}{b}$	unit vector in the residual normal space (direction of the imposed novelty)	§3.2
ψ	angle between $\hat{b}$ and $\hat{\eta }$	Eq. (11)
$O_{*}$	ideal (veridical) off-manifold novelty, $\beta^{2}/\left(\gamma^{2}+\beta^{2}\right)$	§3.2(i)
**Gate 1** — **dimension excess**		
q	dimension excess, $\text{d}-{\text{d}}_{\text{true}}$	§5.2
$L(\overset{ˆ}{b})$	leakage, ${\left\|{{\text{V}}_{\text{d}}}^{⊤}\overset{ˆ}{b}\right\|}^{2}$	Eq. (16)
		
**Gate 5 — where the displacement is booked**		
c	second alignment, ${\hat{n}}_{g}^{⊤}/\left\|\vec{H}\right\|$	Eq. (23)
**Gate 6** — **ratio conditioning**		
$C_{6}$	observable conditioning index, $\text{k}\|\text{r}{\|}^{2}/\ell^{2}$	Eq. (25)
**Other**		
R	radius of the validation sphere	§5
$\sigma ,\sigma_{w}$	noise scale, and noise scale along a specified direction	§5.5.1

**Table 2.** The cascade. Each gate is conditional on those above it. “Directly evaluable” means that the relevant quantity can be estimated from the observed reference/test data without an external manifold model or synthetic calibration. Partial indicates that only part of the gate is directly measurable. The decomposition is therefore conditional: Gates 0–2 establish whether a local geometric estimate is trustworthy; Gate 3 quantifies anchor bias; Gate 4 tests whether the gain axis is defined and sufficiently aligned; Gate 5 determines how anchor bias is apportioned between G and O; and Gate 6 determines whether the resulting ratio is numerically informative.

Gate	Identifiability question	Diagnostic quantity	Directly evaluable?	Cost of failure / interpretation
0	Does a local chart exist?	Local sheet separation relative to neighborhood extent and noise	No	$\hat{\mu }$ and ${\hat{V}}_{d}$ become unreliable; mixed sheets create artifacts that mimic gain or novelty.
1	Is d right?	$q=d-d_{\text{true}}$ stability of the estimated tangent space as d varies	No	Over-estimating d removes directions from the normal complement, corrupting O and potentially destroying the gain axis.
2	Given a valid chart and d, is the estimate good?	$\left\|{\hat{V}}_{d}-V_{d}\right\|$ and gain-axis-direction error	No	Frame-estimation error rotates the tangent/normal decomposition. In the tested regime it is bounded by simulation and is small at $d=d_{\text{true}}$.
3	Where is the anchor?	Anchor bias: $\left(\ell^{2}/2\right)\vec{H}$; anchor jitter: $\ell /\sqrt{\text{k}}$	*Partial*	ℓ and k are measurable, but $\vec{H}$ is not estimated here. Curvature-induced anchor bias is the dominant systematic error.
4	Does the gain axis exist and remain usable?	$a=\left\|P_{N}\hat{\rho }\right\|$	**Yes**	a=0: gain axis is undefined. 0<a≪1: defined but ill-conditioned. Decreasing a can convert G into O.
5	Where is the anchor displacement booked?	$c={\hat{n}}_{g}^{⊤}\vec{H}/\|\vec{H}\|$	No	Requires the mean-curvature direction $\vec{H}.|\text{c}|=1$ routes anchor displacement to G; c = 0 routes it to spurious O.
6	Is the G/O ratio well conditioned?	${\text{C}}_{6}=\text{k}\|\text{r}{\|}^{2}/\ell^{2}$, computed per probe; C_6_ – 1 ≫ 1	Yes	When the leading denominator is comparable to anchor jitter, the reported O becomes unstable or uninformative. Table S17 gives the recovery error by range of C_6_.

## Data Availability

The MATLAB code that generates every supplement table is available at https://github.com/McKenzieNeuro/McKenzieLab/tree/main/GTO.
