# Supplementary material for: Identifying Neural State Changes due to Gain versus Off-Manifold Displacement
Source: ArXiv. 2026 Sep 18:arXiv:2609.21272v1. Preprint. [Version 1] (PMC13596614)
Supplement: Supplementary file 1 [file NIHPP2609.21272V1-supplement-1.pdf]

# Supplement: proofs and simulation tables

Identifying Neural State Changes due to Gain versus Off-Manifold Displacement

Section S1 gives the methods common to every table below: reference sampling, probe construction, the neighborhood extent $\ell$, noise, degeneracy, and aggregation. Section S2 collects the proofs. Everything from Section S4 onward is simulation: what the sweeps measured, on the manifolds they were measured on. Only Table S1 checks an exact identity; every other prediction is a local asymptotic expansion or a model-specific scaling, with scope as stated in the main text.

**Typographic convention.** Where a table tests a closed-form prediction, the rightmost column or columns are headed **ratio** and give measured divided by predicted, using the columns named in the caption; a value of 1 means the prediction is recovered, and departures are the quantity of interest. Tables with no prediction, or whose prediction can be zero, carry no ratio column.

# S1 Methods

This section collects the details common to every table: how the reference set and probes are drawn, how the neighborhood extent is estimated, where noise enters, when a point is flagged degenerate, and how many trials and seeds each table uses. Sections S2 onward give each table's own parameters in its caption; this section is the reference for what those parameters mean and is cited from there rather than repeated.

## S1.1 Reference sampling

Reference sets are drawn per manifold, aiming for uniform area density in every case; the method differs because the manifolds differ.

- **Full sphere.** $v \sim \mathcal{N}(0, I_3)$, normalized to $\|v\| = 1$, then scaled by $R$. Used in Tables S2, S3 (noise-dominated and curvature-dominated blocks), S13, S17, and the $k$ and $R$ blocks of Table S4.
- **Spherical cap, exact.** Uniform in $\cos\theta$ ($\cos\theta = \cos(\text{cap}) + (1 - \cos(\text{cap}))\, u$, $u \sim \text{Unif}(0, 1)$), which is uniform in area on the cap exactly, at any cap angle. Used in the cap block of Table S9 and throughout Table S10.
- **Spherical cap, small-angle.** Uniform in the polar angle itself via $\theta = \text{cap} \cdot \sqrt{u}$ (the construction that gives uniform area on a *flat* disc), rather than in $\cos\theta$. This is only approximately uniform in area on a sphere, with relative error $O(\text{cap}^2)$ from $\sin\theta \approx \theta$; at the cap angles used here (0.25–0.35 rad) the error is a percent or two. Used in Tables S11, S12 and S6, where the cap is small enough for the approximation to be immaterial next to the effects under study.
- **Flat disc.** Uniform via $r = a\sqrt{u}$, $\phi \sim \text{Unif}(0, 2\pi)$, exact for a flat patch. Used for the quadric patches of Table S7 and the curvature-gradient patch of Table S5.
- **Torus of revolution.** Rejection sampling in $\theta$ against density $\propto R_c + r\cos\theta$, uniform in $\phi$. Used in Table S8.
- **Clifford torus.** Uniform in $(u, v)$ directly: the flat product metric makes this exact. Used in Table S14.
- **Circle × ellipse.** Uniform in the circle coordinate; rejection sampling in the ellipse coordinate against density $\propto \sqrt{A^2 \sin^2 v + B^2 \cos^2 v}$. Used in Table S15.

## S1.2 Probe construction

Every probe construction falls into one of two families, chosen so that the quantity a table reports is not confounded with the anchor-tracking behavior of Section S2.

**Radial and ambient legs on an origin-centered sphere.** A probe is

$$z_B = m + \nu\big(\text{sgn}\sqrt{1 - O_\star}\; \hat{\rho} + \sqrt{O_\star}\; \hat{o}\big), \tag{S1}$$

with $\hat{\rho} = m/\|m\|$ the radial direction and $\hat{o}$ a direction orthogonal to the sphere's ambient span. Because every reference point has the same norm $R$, and $\hat{o}$ is orthogonal to the span both $m$ and every reference point occupy, the squared distance from $z_B$ to any reference point $x$ changes by the same constant under this construction; $k$-NN selection is therefore exactly invariant to $\nu$, $O_\star$ and

sgn (the main text's Case 2, Section 3.1.1.1). This isolates whatever the table is testing from the anchor's own tendency to track the imposed displacement. Used in Tables S9, S10, S11, S16, S17, and the rotation and boundary tests of Tables S4, S5 and S6 (pure off-manifold probes, $O_\star = 1$).

**On-manifold steps, referred to the probe's own projection.** A probe is placed by an exact step along the manifold (a geodesic on the sphere or Clifford torus, a coordinate step on the torus of revolution or the circle × ellipse), and the anchor displacement is measured against the probe's own base point rather than a point fixed before the step. This is necessary whenever the manifold is inhomogeneous or curved, where referring to a pre-step point would contaminate the measurement with the probe's own curvature-driven offset (Table S8 documents this confound directly). Used in Tables S2, S8, S13, S14 and S15.

### S1.3 Neighborhood extent

Given the $k$ reference points nearest a test point, with centroid $\mu$ and tangent basis $V_d$ (the leading $d$ right singular vectors of the mean-centered neighbor matrix), two extents are computed:

$$\ell^2 = \frac{1}{k-1}\sum_{j=1}^{k} \|V_d^\top (x_j - \mu)\|^2 \qquad \text{tangential, unbiased} \tag{S2}$$

$$\rho^2 = \frac{1}{k-1}\sum_{j=1}^{k} \|x_j - \mu\|^2 \qquad \text{ambient, unbiased}$$

$\ell$ is the neighborhood extent of Propositions 2–3 and is used in every closed-form prediction in this supplement (`D.ell` in the code); $\rho$ additionally includes normal-direction spread and any noise not confined to the tangent space, and is not used in predictions (`D.rnbr`). Both divide by $k-1$ rather than $k$: since $\mu$ is the sample mean of the same $k$ points, dividing by $k$ underestimates the population second moment by a factor $(k-1)/k$, which appears as a systematic $k/(k-1)$ excess in any ratio built from the biased form.

A third value, the areal estimate $k \cdot \text{Area}/(2\pi n_{\text{ref}})$, appears in Table S8 and the $k$ sweep of Table S14, where $\ell$ cannot be measured from a fixed base point beforehand. It must be multiplied by the $k$-NN boundary factor $(k+1)/k$ to match $\ell^2$: the $k$-th nearest neighbor sits on the boundary of the occupied disc, so the sampled region is larger by that factor than the area estimate assumes (Section S2).

On a sphere of radius $R$ with $n_{\text{ref}}$ points uniform over the full sphere, $\ell^2 \approx 2(k+1)R^2/n_{\text{ref}}$ to leading order (Eq. 21 of the main text); the sagitta $\delta = \ell^2/2R$ carries a further next-order correction, $\delta = (\ell^2/2R)(1+\ell^2/3R^2)$, from the exact spherical geometry (Eq. (S3)), tested directly by the *ratio2* columns of Tables S9, S10 and S13.

### S1.4 Noise

Where a table has a noise parameter $\sigma$, it is added as $\mathcal{N}(0, \sigma^2 I_F)$ independently to every one of the $F$ ambient coordinates, applied to the reference set, the probes, or both as stated in that table's caption; Table S9's noise block and Table S3 add it to both. Anisotropic noise (Tables S10–S12) instead uses $\Sigma = \sigma^2(I + \kappa\, ww^\top)$ for a unit vector $w$ placed along a stated direction (the gain axis, a tangent direction, or an ambient direction), so that $\sigma_w = \sigma\sqrt{1+\kappa}$ is the noise scale along $w$. Tables S7, S5 and S6 are noiseless by construction, since they isolate a purely geometric effect (curvature, or a sampling boundary) from any noise contribution. Table S14 uses a small isotropic $\sigma = 0.001$ so that the noise floor $\sqrt{F-3}\,\sigma$ is available as a reference scale without swamping the geometric signal; Table S15 and the main block of Table S8 are noiseless.

### S1.5 Degeneracy

A point is flagged degenerate when the estimated alignment $a = \|P_N \hat{\rho}\|$ falls below `alignTol`, 0.1 by default; $G$ and $O$ are then undefined (`NaN`) and excluded from every summary in this supplement, while $T$, which does not use the gain axis, remains defined and included. The threshold is scientific rather than numerical: on a cone, where the gain axis is undefined in the continuum, the measured alignment is about 0.06, not machine epsilon, because finite sampling, noise and curvature keep $\|P_N \hat{\rho}\|$ off zero even in the fully degenerate case (`gto2_checks`, check C2). Reported degeneracy fractions (columns headed %degen. or fracDegen) are the share of probes excluded on this basis in that cell.

### S1.6 Aggregation and replication

Summaries are medians over probes unless a table states otherwise; several tables in Section S5 and Section S11 report both medians and means explicitly, because the quantities involved (leakage, the recovered off-manifold fraction) are asymmetrically distributed and a median-only or mean-only comparison can appear to disagree with a closed-form prediction that is exact only for one of the two (Table S3's survival law, checked against both, is the clearest case: the earlier apparent median/mean discrepancy was the missing $q = 0$ leakage floor, not a choice between summaries). Where a table reports a standard error, it is a Monte Carlo SE over the stated number of seeds, each seed redrawing the full reference set and probe set independently; single-seed tables report no SE. Seed counts and trials per cell are given in each table's caption.

### S1.7 Software and code availability

Simulations were run in MATLAB R2026a. The code that generates every table in this supplement is available at `https://github.com/McKenzieNeuro/McKenzieLab/tree/main/GTO`, run via a single entry point (`gto2_run_all`) that regenerates all tables in the dependency order of Section S12 and logs each step.

## S2 Proofs and derivations

The main text states Propositions 1–3 and the corollaries drawn from them; the derivations are collected here.

### Definition 1 (orthogonality of the three subspaces)

$T_p\mathcal{M}$ and $N_p\mathcal{M}$ are orthogonal complements by definition, and $\hat{n}_g \in N_p\mathcal{M}$, so $G_p \perp T_p\mathcal{M}$. $R_p$ is defined as $N_p\mathcal{M} \cap G_p^{\perp}$, so $R_p \perp G_p$ and $R_p \subseteq N_p\mathcal{M}$ gives $R_p \perp T_p\mathcal{M}$. Within $N_p\mathcal{M}$, $G_p$ is one-dimensional and $R_p$ is its orthogonal complement, so $G_p \oplus R_p = N_p\mathcal{M}$ and hence $T_p\mathcal{M} \oplus G_p \oplus R_p = \mathbb{R}^F$. Orthogonal direct sums give unique resolutions and Pythagorean norms, which is the content of the definition. The construction requires only $P_N\hat{\rho} \neq 0$, so that $\hat{n}_g$ is defined; where that fails, $G_p$ and $R_p$ are not defined and neither is the decomposition. □

Note that no fourth component exists. Since $R_p$ is the whole orthogonal complement of $G_p$ inside $N_p\mathcal{M}$, the three subspaces already exhaust $\mathbb{R}^F$, and any further residual is identically zero. Distinguishing residual-normal displacement that the reference geometry can account for from displacement it cannot would require a further privileged direction inside $R_p$; the present construction supplies none, and treats all of $R_p$ as novelty.

### Proposition 1 (exact decomposition of the fractions)

*Proof.* By construction $\hat{n}_g \perp V_d$, so $\mathrm{span}(V_d)$, $\mathrm{span}(\hat{n}_g)$ and the residual normal space are mutually orthogonal, and $r = (z_B - m) + (m - \mu)$ resolves uniquely across them. The gain-axis component is $\gamma + \delta$; the residual-normal component is $\beta\hat{b} + \vec{\eta}$, of squared norm $\beta^2 + \eta^2 + 2\beta\eta\cos\psi$; the tangential component has squared norm $\tau^2$. Pythagoras gives the stated $\|r\|^2$ and the fractions follow by definition. No approximation is made, so a numerical departure is a coding error rather than a tolerance question. □

The statement assumes that the imposed change $z_B - m$ has no component in the tangent space *of the anchor frame*. In general the tangential term is $\|P_T(z_B - m) + P_T(m - \mu)\|^2$ rather than $\tau^2$; Table S1 checks that general form and reports the share of $\|r\|^2$ the imposed change actually places there.

### Consequences of Proposition 1

*(ii) First order.* Writing $\nu^2 = \gamma^2 + \beta^2$ and $O_\star = \beta^2/\nu^2$, and ordering terms in the neighborhood extent — $\delta, \eta = O(\ell^2)$, $\tau^2 = O(\ell^2/k)$, while $\delta^2$ and $\eta^2$ are $O(\ell^4)$ and dropped —

$$O - O_\star = \frac{2\gamma^2\beta\eta\cos\psi}{\nu^4} - \frac{2\beta^2\gamma\delta}{\nu^4} - \frac{\beta^2\tau^2}{\nu^4},$$

which is the form quoted in the main text after collecting $O_\star$.

*(iii) Zero imposed novelty.* Setting $\beta = 0$ in the exact expression leaves $O = \eta^2/[(\gamma+\delta)^2+\eta^2+\tau^2]$ directly. The first-order form carries $1/\beta$ in its $\eta$ term and is not valid in this limit.

*(iv) Random novel direction.* For $\hat{b}$ uniform on the unit sphere of a residual normal space of dimension at least two, $\mathbb{E}[\hat{b} \cdot \hat{\eta}] = 0$ by symmetry, so the cross term has zero mean.

### Proposition 2 (anchor displacement)

Let $e_1, \ldots, e_d$ be an orthonormal basis of $T_p\mathcal{M}$ and $\mathrm{I\!I}$ the second fundamental form, so that a neighbouring manifold point with chart coordinate $u$ is $x(u) = p + \sum_i u_i e_i + \frac{1}{2}\mathrm{I\!I}(u,u) + O(\|u\|^3)$.

*Proof.* Averaging over the $k$ neighbours about $p$ gives $\mu - p = \bar{u} + \frac{1}{2}\mathrm{II}(\overline{uu^\top}) + O(\ell^3)$ with $\bar{u} = k^{-1}\sum_j u_j$. Taking expectations, $\mathbb{E}[\bar{u}] = 0$ and $\mathbb{E}[\overline{uu^\top}] = (\ell^2/d)I_d$ for a locally isotropic measure, so by bilinearity $\frac{1}{2}\mathrm{II}(\mathbb{E}[\overline{uu^\top}]) = (\ell^2/2d)\sum_i \mathrm{II}(e_i, e_i) = (\ell^2/2)\vec{H}$. □

The neglected term is $O(\ell^3)$ in general and $O(\ell^4)$ when the local sampling measure is symmetric, since the cubic contribution contracts $\nabla\mathrm{II}$ against the third moment of $u$.

Here $\ell^2 = \mathbb{E}\|u\|^2$ over the $k$ neighbours of the probe. The $k$-th neighbour lies on the boundary of the selection ball and the other $k-1$ are uniform inside it, so for locally uniform sampling $\ell^2$ is $(k+1)/k$ times the areal value $k\,\mathrm{Area}/(2\pi n_{\mathrm{ref}})$. When $\mathbb{E}[u] = 0$ the centered estimator $\sum_j \|u_j - \bar{u}\|^2/(k-1)$ is unbiased for $\ell^2$.

### The spherical case

For a sphere of radius $R$ with outward unit normal $\hat{n}$, $\mathrm{II}(e_i, e_i) = -\hat{n}/R$ for every $i$, so $\vec{H} = -\hat{n}/R$ and $\|\mathbb{E}[\mu] - p\| = \ell^2/2R$ directed inward, independent of $d$ and of $F$. When the sphere is centered at the origin the normal is the radial direction, so this displacement is collinear with the gain axis.

With $\ell$ measured in the tangent plane the exact sphere supplies the next order. A neighbour at tangential radius $\rho$ lies $R - \sqrt{R^2 - \rho^2} = \rho^2/2R + \rho^4/8R^3 + O(\rho^6/R^5)$ below the tangent plane, and a uniformly filled disc has $\mathbb{E}\rho^4 = \frac{4}{3}(\mathbb{E}\rho^2)^2$, so the inward displacement is

$$\delta = \frac{\ell^2}{2R}\Big(1 + \frac{\ell^2}{3R^2}\Big) + O(\ell^6/R^5). \tag{S3}$$

The columns headed ratio2 in Tables S9, S10 and S13 test this form.

### Proposition 3 (anchor jitter)

$\mathbb{E}\|\bar{u}\|^2 = k^{-1}\,\mathrm{tr}\,\mathbb{E}[uu^\top] = \ell^2/k$ for independent zero-mean samples, so the root-mean-square tangential fluctuation is $\ell/\sqrt{k}$. Under locally uniform sampling $\ell \propto k^{1/d}$, hence $\ell/\sqrt{k} \propto k^{1/d-1/2}$: decreasing in $k$ for $d > 2$, exactly $k$-invariant for $d = 2$, increasing for $d = 1$, while the bias of Proposition 2 grows as $k^{2/d}$ for every $d$.

### The covariance tilt

The second fundamental form $\mathrm{II}$ is a symmetric bilinear map $T_p\mathcal{M} \times T_p\mathcal{M} \to N_p\mathcal{M}$, and its derivative $\nabla\mathrm{II}$ the corresponding symmetric trilinear map. Restricting to a single tangent direction $e_1$ and a single normal direction $\hat{n}$ collapses both to scalars: bilinearity gives $\mathrm{II}(u_1e_1, u_1e_1) = u_1^2\,\mathrm{II}(e_1, e_1)$, and projecting onto $\hat{n}$ leaves $\kappa = \hat{n}^\top\mathrm{II}(e_1, e_1)$, the ordinary normal curvature in direction $e_1$ seen along $\hat{n}$; the same restrictions give $g = \hat{n}^\top\nabla\mathrm{II}(e_1, e_1, e_1)$, to leading order the rate of change of $\kappa$ along $e_1$. The local model is then

$$w(u_1) = \tfrac{1}{2}\kappa\,u_1^2 + \tfrac{1}{6}g\,u_1^3 + O(u_1^4). \tag{S4}$$

For this patch the tilt of the fitted plane is the regression slope $\mathbb{E}[u_1w]/\mathbb{E}[u_1^2]$, valid to leading order while $\mathbb{E}[ww^\top] \ll \mathbb{E}[uu^\top]$. The quadratic part contributes $\frac{\kappa}{2}\mathbb{E}[u_1^3] = 0$ by parity and the cubic part survives, giving

$$\theta \simeq \frac{g}{6}\cdot\frac{\mathbb{E}[u_1^4]}{\mathbb{E}[u_1^2]}.$$

The fourth-moment ratio is a property of the sampling distribution, not of the geometry: for a uniformly filled disc of radius $a$ it equals $a^2/2 = \ell^2$, giving $\theta \simeq g\ell^2/6$, but the coefficient would differ for a Gaussian neighborhood. Since the rotation is $\|V_d^\top\hat{n}\|^2 = \sin^2\theta \approx \theta^2$, this gives

$$\|V_d^\top\hat{n}\|^2 \;\propto\; \ell^4 \;\propto\; k^2, \tag{S5}$$

using $\ell^2 \propto k$ for this sampling regime. The $\ell^4$ scaling is the robust part; the prefactor is not.

The same calculation shows why a curvature gradient does not disturb Proposition 2: the *mean* of $w$ retains the quadratic term and loses the cubic to parity, while the *covariance* loses the quadratic and retains the cubic. Curvature and its gradient are read by two different statistics of the same neighborhood and do not leak into one another under symmetric sampling.

## The finite-sample tilt floor

Under symmetric sampling the covariance vanishes in expectation but not in a finite sample, so the fitted plane tilts at random. Take the normal displacement $w = \|u\|^2/2R$ (a sphere, $\kappa = 1/R$). The least-squares slope along tangent direction $i$ is $\widehat{\mathrm{Cov}}(u_i, w)/\widehat{\mathrm{Var}}(u_i)$. For $k$ points uniform in a disc of radius $a$, $\mathbb{E}u_i^2 = a^2/4$, $\mathbb{E}[u_i^2\|u\|^2] = a^4/6$ and $\mathbb{E}[u_i^2\|u\|^4] = a^6/8$, so

$$k\,\mathrm{Var}\,\widehat{\mathrm{Cov}}(u_i, \|u\|^2) = \mathbb{E}[u_i^2\|u\|^4] + (\mathbb{E}\|u\|^2)^2\,\mathbb{E}u_i^2 - 2\,\mathbb{E}\|u\|^2\,\mathbb{E}[u_i^2\|u\|^2] = \frac{a^6}{48},$$

and $\mathrm{Var}(\mathrm{slope}_i) = a^2/(12kR^2)$ at leading order in $1/k$. Summing the two tangent directions and using $a^2 = 2\ell^2$,

$$\mathbb{E}\,\|V_d^\top \hat{n}\|^2 = \frac{\ell^2\kappa^2}{3k}\left[1 + O(1/k)\right]. \tag{S6}$$

A one-direction patch $w = \frac{1}{2}\kappa u_1^2$ gives the same total ($\kappa^2 a^2/8k$ along $u_1$ plus $\kappa^2 a^2/24k$ along $u_2$). For a uniformly sampled 2-sphere, $\ell^2 = 2kR^2/n_{\mathrm{ref}}$ and the floor is $2/(3n_{\mathrm{ref}})$, independent of $k$ and $R$. The heuristic that the fitted plane is the tangent plane at the displaced centroid gives $2/n_{\mathrm{ref}}$; the least-squares plane tilts one third as much. With two approximately Gaussian slopes the rotation is approximately exponential, so its median is near $\ln 2$ times its mean. The $O(1/k)$ factor is measured directly in Table S5.

## The survival law

The survival law of the main text is a standard fact about random subspace projections rather than a result derived here. By rotational invariance the fixed direction may be held and the subspace randomised, so the squared projection of a unit vector onto a uniformly random (Haar) $q$-dimensional subspace of $\mathbb{R}^m$ is $\sum_{i=1}^q g_i^2 \big/ \sum_{i=1}^m g_i^2$ for i.i.d. standard Gaussians $g_i$: a ratio of chi-squared variates, hence $\mathrm{Beta}(q/2,\,(m-q)/2)$, with mean $q/m$. Survival is the complement, giving $1 - q/(F - d_{\mathrm{true}})$ with $m = F - d_{\mathrm{true}}$.

The Beta median has no closed form but is easily evaluated numerically; at $F = 30$, $d_{\mathrm{true}} = 2$ the median survival exceeds the mean by 0.017–0.020 for $q =$ 1–4, because the leakage distribution is right-skewed. A comparison must therefore be like for like: median survival against the median law, mean against the mean.

A second correction matters at the same scale. At $q = 0$ the estimated tangent space already absorbs a small leakage $L_0$ of $\hat{b}$, since noise tilts it, and the excess directions act on what remains. Survival is therefore modelled as $(1 - L_0)(1 - B)$, with $B \sim \mathrm{Beta}(q/2, (m-q)/2)$ and $L_0$ drawn from the measured per-probe $q = 0$ leakage, assuming independence. With both corrections the noise-dominated survivals of Table S3 agree with the law to within 0.007, for medians and means alike; without the floor the median form misses by up to 0.012 and the mean form by up to 0.009. Where the excess directions are structured rather than noise-driven the law fails outright, which is the curvature-dominated block of the same table.

### The curvature alignment on a Clifford torus

For $p(u,v) = (R_1 \cos u, R_1 \sin u, R_2 \cos v, R_2 \sin v)$ the normal space is spanned by $n_1 = (\cos u, \sin u, 0, 0)$ and $n_2 = (0, 0, \cos v, \sin v)$, with $\mathrm{I\!I}(e_u, e_u) = -n_1/R_1$ and $\mathrm{I\!I}(e_v, e_v) = -n_2/R_2$. Hence $\vec{H} = -\frac{1}{2}(n_1/R_1 + n_2/R_2)$ and $\|\vec{H}\| = \frac{1}{2}\sqrt{R_1^{-2} + R_2^{-2}}$. The position vector $p = R_1 n_1 + R_2 n_2$ lies wholly in the normal space, so $\hat{n}_g = \hat{\rho}$ and $\hat{n}_g^\top \vec{H} = -\frac{1}{2}(1+1)/\rho = -1/\rho$ with $\rho = \sqrt{R_1^2 + R_2^2}$. Then

$$c = -\frac{1}{\rho\|\vec{H}\|} = -\frac{2}{\sqrt{R_1^2 + R_2^2}\,\sqrt{R_1^{-2} + R_2^{-2}}},$$

which is $-1$ at $R_1 = R_2$ and falls in magnitude as the radii are separated.

## S3 The exact identity

Proposition 1 is an algebraic identity in the anchor frame, so it admits a check of a different class from everything else here: a departure is a coding error, not a tolerance question. Every quantity is computed from the *realized* $\mu$, $V_d$ and $\hat{n}_g$: $\gamma = \hat{n}_g^\top(z_B - m)$, $\delta = \hat{n}_g^\top(m - \mu)$, $\beta\hat{b}$ and $\vec{\eta}$ the residual-normal parts of $z_B - m$ and $m - \mu$, and $t = P_T(z_B - m) + P_T(m - \mu)$, so that $\|r\|^2 = (\gamma + \delta)^2 + \|\beta\hat{b} + \vec{\eta}\|^2 + \|t\|^2$. Imposed changes have random composition ($O_\star$ uniform on $[0, 1]$), magnitude ($\nu$ uniform on $[0.3, 1]$) and gain sign. The last column is the median share of $\|r\|^2$ that the imposed change places in the anchor tangent space, which Proposition 1 as stated takes to be zero.

Table S1: Exact identity, maximum absolute departure over 300 probes per seed. Sphere, $R = 5$, $F = 30$, $d = 2$, $n_{\text{ref}} = 4000$, $k = 40$, $\sigma = 0.01$.

| seed | $\big\lvert\, \|r\|^2_{\text{pred}} - \|r\|^2 \,\big\rvert$ | $\lvert\Delta G\rvert$ | $\lvert\Delta T\rvert$ | $\lvert\Delta O\rvert$ | $\lvert G{+}T{+}O - 1\rvert$ | imposed $T$ share |
|---|---|---|---|---|---|---|
| 0 | $6.7 \times 10^{-16}$ | $2.4 \times 10^{-15}$ | $4.7 \times 10^{-16}$ | $1.9 \times 10^{-15}$ | $3.0 \times 10^{-15}$ | $4.02 \times 10^{-4}$ |
| 1 | $4.4 \times 10^{-16}$ | $2.4 \times 10^{-15}$ | $4.2 \times 10^{-16}$ | $2.4 \times 10^{-15}$ | $3.1 \times 10^{-15}$ | $3.50 \times 10^{-4}$ |
| 2 | $5.6 \times 10^{-16}$ | $2.2 \times 10^{-15}$ | $5.6 \times 10^{-16}$ | $2.9 \times 10^{-15}$ | $3.2 \times 10^{-15}$ | $4.36 \times 10^{-4}$ |

## S4 Gate 0 — sheet proximity

Two concentric spherical shells separated by $h$, both origin-centered, with probes lying exactly on the inner shell. The imposed change is purely on-manifold, so all reported energy is artefact. The second block repeats the test with the sheets offset in an ambient direction instead of radially.

Table S2: Sheet proximity. $R = 5$, $F = 30$, $d = 2$, $n_{\text{ref}} = 6000$ split evenly between sheets, $k = 40$, 300 probes, $\sigma = 0.01$; medians over probes, means over 5 seeds. "wrong" is the fraction of neighbours drawn from the other sheet. Control is a single sheet. The noise floor for $\|r_{\text{novel}}\|$ is $\sqrt{F-3}\,\sigma = 0.0520$.

| stacking | $h$ | $h/\ell$ | wrong | $\|r_{\text{rad}}\|$ | $\|r_{\text{novel}}\|$ | $\|r\|$ |
|---|---|---|---|---|---|---|
| radial | 4.00 | 4.89 | 0.0% | 0.06600 | 0.05214 | 0.13920 |
| | 1.00 | 1.36 | 11.4% | 0.06195 | 0.05219 | 0.14020 |
| | 0.50 | 0.82 | 38.8% | **0.15747** | 0.05231 | 0.19021 |
| | 0.25 | 0.42 | 46.7% | 0.08236 | 0.05238 | 0.12934 |
| | 0.10 | 0.17 | 49.3% | 0.01608 | 0.05239 | 0.09687 |
| | 0.05 | 0.09 | 49.8% | 0.01051 | 0.05240 | 0.09432 |
| | control | — | 0.0% | 0.06600 | 0.05214 | 0.13920 |
| ambient | 4.00 | 4.89 | 0.0% | 0.06600 | 0.05214 | 0.13920 |
| | 1.00 | 1.38 | 12.6% | 0.04803 | 0.13661 | 0.18495 |
| | 0.50 | 0.85 | 40.6% | 0.02600 | **0.20959** | 0.23105 |
| | 0.25 | 0.43 | 47.8% | 0.03036 | 0.13058 | 0.15932 |
| | 0.10 | 0.17 | 49.8% | 0.03305 | 0.07246 | 0.11215 |
| | 0.05 | 0.09 | 50.0% | 0.03347 | 0.05807 | 0.10310 |
| | control | — | 0.0% | 0.06600 | 0.05214 | 0.13920 |

Under radial stacking $\|r_{\text{novel}}\|$ stays at the noise floor across every separation, 0.0521–0.0524 against 0.0520, while $\|r_{\text{rad}}\|$ peaks at 2.39× control at $h/\ell = 0.82$. Under ambient stacking the pattern inverts: $\|r_{\text{novel}}\|$ peaks at 4.0× the floor at $h/\ell = 0.85$, while $\|r_{\text{rad}}\|$ stays at or below control. The control's $\|r_{\text{rad}}\|$ is the sagitta itself ($\ell^2/2R = 0.067$). At $h \ll \ell$ the two sheets act as one denser, thicker sheet and the artefact subsides. The wrong-sheet fraction is largest (50%) exactly where the artefact is smallest, so it is not a diagnostic.

## S5 Gate 1 — dimension excess

Leakage $\|V_d^\top \hat{b}\|^2$ for an imposed purely ambient $\hat{b}$, swept over $d$. The upper table compares survival with the survival law at a noise-dominated and a curvature-dominated noise level; the lower table sweeps the noise through the transition between them at $q = 1$. "Capture" is the median squared overlap of the third singular vector of the neighborhood with the true normal at the base point: near 1 when curvature wins that slot, near the chance value 0.017 (median of Beta($\frac{1}{2}, \frac{27}{2}$)) when noise does.

Table S3: Dimension excess on a sphere. $R = 5$, $F = 30$, $d_{\text{true}} = 2$, $n_{\text{ref}} = 4000$, $k = 40$, 2500 probes × 3 seeds. Survival is $1 - \|V_d^\top \hat{b}\|^2$, summarized per seed by median or mean and averaged over seeds (sd across seeds in parentheses). Haar med. and Haar mean are the median and mean of $1 - \text{Beta}(q/2, (F-d)/2)$; ×floor folds in the per-probe $q = 0$ leakage, $(1 - L_0)(1 - B)$, by Monte Carlo ($10^5$ draws). Ratio columns are measured over the floor-corrected prediction of the same summary. $a$ is the median alignment.

| | $d$ | med. (sd) | mean | Haar med. | ×floor | Haar mean | ×floor | $a$ | ratio (med.) | ratio (mean) |
|---|---|---|---|---|---|---|---|---|---|---|
| $\sigma = 0.30$ | 2 | 0.9925 (0.0002) | 0.9888 | 1.0000 | 0.9925 | 1.0000 | 0.9887 | 0.9959 | 1.000 | 1.000 |
| | 3 | 0.9708 (0.0007) | 0.9557 | 0.9830 | 0.9698 | 0.9643 | 0.9534 | 0.9827 | 1.001 | 1.002 |
| | 4 | 0.9390 (0.0007) | 0.9211 | 0.9481 | 0.9365 | 0.9286 | 0.9181 | 0.9629 | 1.003 | 1.003 |
| | 5 | 0.9049 (0.0005) | 0.8874 | 0.9114 | 0.9006 | 0.8929 | 0.8829 | 0.9422 | 1.005 | 1.005 |
| | 6 | 0.8695 (0.0010) | 0.8542 | 0.8742 | 0.8638 | 0.8571 | 0.8475 | 0.9212 | 1.007 | 1.008 |
| $\sigma = 0.01$ | 2 | 1.0000 (0.0000) | 1.0000 | 1.0000 | 1.0000 | 1.0000 | 1.0000 | 0.9999 | 1.000 | 1.000 |
| | 3 | 0.9985 (0.0001) | 0.9963 | 0.9830 | 0.9830 | 0.9643 | 0.9643 | 0.2939 | 1.016 | 1.033 |
| | 4 | 0.9782 (0.0006) | 0.9598 | 0.9481 | 0.9481 | 0.9286 | 0.9286 | 0.2869 | 1.032 | 1.034 |
| | 5 | 0.9422 (0.0012) | 0.9225 | 0.9114 | 0.9114 | 0.8929 | 0.8930 | 0.2797 | 1.034 | 1.033 |
| | 6 | 0.9047 (0.0013) | 0.8855 | 0.8742 | 0.8741 | 0.8571 | 0.8572 | 0.2722 | 1.035 | 1.033 |

| $\sigma$ | survival ($q$=1) | median $O$ | % degen. | capture | $a$ | ratio $a/\sqrt{1-\text{capture}}$ |
|---|---|---|---|---|---|---|
| 0.005 | 0.9996 | 0.9229 | 5.0 | 0.979 | 0.1452 | 1.002 |
| 0.010 | 0.9985 | 0.9225 | 0.0 | 0.913 | 0.2939 | 0.996 |
| 0.020 | 0.9936 | 0.9232 | 0.0 | 0.634 | 0.6046 | 0.999 |
| 0.030 | 0.9872 | 0.9234 | 0.0 | 0.241 | 0.8707 | 0.999 |
| 0.050 | 0.9828 | 0.9235 | 0.0 | 0.050 | 0.9741 | 0.999 |
| 0.080 | 0.9816 | 0.9271 | 0.0 | 0.027 | 0.9853 | 0.999 |
| 0.120 | 0.9801 | 0.9333 | 0.0 | 0.022 | 0.9874 | 0.998 |
| 0.200 | 0.9758 | 0.9438 | 0.0 | 0.020 | 0.9855 | 0.996 |
| 0.300 | 0.9708 | 0.9456 | 0.0 | 0.020 | 0.9827 | 0.993 |

**The survival law.** Compared like for like, and with the $q = 0$ floor folded in, the noise-dominated survivals exceed the law by 0.001–0.006 in medians and 0.002–0.007 in means for $q = 1$–4, against seed-to-seed scatter of at most 0.001. The residual is systematic and grows with $q$, so the excess directions of a local PCA are close to Haar-distributed but not exactly. Without the floor, the median form misses by up to 0.012 and the mean form by up to 0.009 (Section S2).

**The curvature-dominated case.** At $\sigma = 0.01$ the median leakage at $q = 1$ is 0.0015 against a Haar median of 0.017 (mean 0.0037 against 0.036): the absorbed direction is the normal, not a novelty-carrying one. The alignment collapses from 1.000 to 0.294 in that single step and stays near 0.28 as $d$ grows. Dimension over-estimation damages gate 4, not gate 1's own quantity.

**The transition.** Capture falls from 0.98 at $\sigma = 0.005$ to the chance level by $\sigma = 0.08$, crossing 0.5 near $\sigma \approx 0.023$. That is about half the sagitta $\ell^2/2R = 0.050$, and 0.8 times its spread along the normal for a uniformly filled disc, $\ell^2/(2\sqrt{3}R) = 0.029$. The alignment at $q = 1$ follows $\sqrt{1 - \text{capture}}$ to within 0.7% (capture is printed to three decimals), because on this sphere the gain axis is the normal and $a^2 = 1 - \|V_d^\top \hat{n}\|^2$ up to the gate-2 rotation floor. The drop in $a$ between $d$ and $d+1$ is therefore a direct, observable reading of how much of the normal the extra dimension has captured.

## S6 Gate 2 — tangent rotation

Table S4 measures the squared component, inside the estimated tangent basis, of a direction that is truly normal at the base point, on a noiseless uniformly sampled sphere. The prediction is the finite-sample tilt floor of Eq. (S6): mean $2/(3n_{\text{ref}})$ and median near $2\ln 2/(3n_{\text{ref}})$, independent of $k$ and $R$ at leading order in $1/k$. Each cell reuses the same draws, so in the $R$ block the construction differs only by a uniform rescaling.

Table S4: Tangent rotation, $\|V_d^\top \hat{n}\|^2 \times n_{\text{ref}}$, on a sphere with pure off-manifold probes. $F = 30$, $d = 2$, 1000 probes per cell, 5 seeds (sd across seeds in parentheses), noiseless reference. Each block varies one parameter, holding the others at their default ($n_{\text{ref}} = 4000$, $k = 40$, $R = 5$). Ratio columns divide by $2\ln 2/3 = 0.462$ (median) and $2/3$ (mean).

| block | value | median (sd) | mean (sd) | ratio (median) | ratio (mean) |
|---|---|---|---|---|---|
| $n_{\text{ref}}$ | 1000 | 0.572 (0.025) | 0.862 (0.043) | 1.238 | 1.293 |
| | 2000 | 0.515 (0.018) | 0.810 (0.043) | 1.114 | 1.215 |
| | 4000 | 0.499 (0.018) | 0.762 (0.029) | 1.080 | 1.143 |
| | 8000 | 0.509 (0.033) | 0.786 (0.045) | 1.101 | 1.179 |
| | 16000 | 0.505 (0.033) | 0.783 (0.010) | 1.093 | 1.175 |
| $k$ | 20 | 0.541 (0.011) | 0.911 (0.019) | 1.171 | 1.367 |
| | 40 | 0.499 (0.018) | 0.762 (0.029) | 1.080 | 1.143 |
| | 80 | 0.483 (0.040) | 0.715 (0.050) | 1.045 | 1.073 |
| | 160 | 0.502 (0.032) | 0.730 (0.024) | 1.086 | 1.095 |
| $R$ | 3 | 0.499 (0.018) | 0.762 (0.029) | 1.080 | 1.143 |
| | 5 | 0.499 (0.018) | 0.762 (0.029) | 1.080 | 1.143 |
| | 12 | 0.499 (0.018) | 0.762 (0.029) | 1.080 | 1.143 |
| | 20 | 0.499 (0.018) | 0.762 (0.029) | 1.080 | 1.143 |

The $R$ block returns identical rows at every radius: with the same draws, a uniformly rescaled configuration selects the same neighbours and the same singular vectors, so the rotation is exactly scale-invariant, as the prediction requires. Across $n_{\text{ref}}$ the median sits at 0.50–0.57 and the mean at 0.76–0.86, both close to flat for $n_{\text{ref}} \geq 4000$ and higher at $n_{\text{ref}} = 1000$, where $\ell/R = 0.28$. Across $k$ the mean falls from 1.37 times the prediction at $k = 20$ to 1.07–1.10 at $k = 80$–160. That is the $O(1/k)$ correction of Eq. (S6), measured directly on the covariance-route patch in Table S5. The $\ell^2 = 2kR^2/n_{\text{ref}}$ used here is the areal value, and contributes a further $(k+1)/k$. The median is 0.59–0.69 of the mean, at or below the exponential value $\ln 2 = 0.69$: the tail is at least exponential, and heaviest at small $k$.

## S7 Gate 2 — the covariance route

A symmetric neighborhood on a homogeneous manifold has $\mathbb{E}[uw^\top] = 0$ by parity, so curvature displaces the anchor without tilting the frame. These two sweeps break that parity deliberately.

Table S5: Curvature gradient. Patch $w = \frac{1}{2}\kappa u_1^2 + \frac{1}{6} g u_1^3$ with $\kappa = 0.2$, uniform disc of radius 1, $F = 30$, $n_{\mathrm{ref}} = 6000$, 2000 trials per cell, noiseless; means. Every cell uses the same draws, so the $g = 0$ rotation at the same $k$ is a paired measurement of the finite-sample floor. Ratio columns: measured floor over $\ell^2\kappa^2/3k$ (Eq. S6); raw rotation over $(g\ell^2/6)^2$; rotation minus the measured floor over $(g\ell^2/6)^2$ (subtr.).

| *g sweep at* $k = 40$ | | | | | | | |
|---|---|---|---|---|---|---|---|
| $g$ | $\ell$ | $\Vert V_d^\top \hat{n}\Vert^2$ | $(g\ell^2/6)^2$ | floor | ratio (floor) | ratio (raw) | ratio (subtr.) |
| 0.00 | 0.0585 | $1.273 \times 10^{-6}$ | 0 | $1.273 \times 10^{-6}$ | 1.115 | — | — |
| 0.50 | 0.0585 | $1.349 \times 10^{-6}$ | $8.142 \times 10^{-8}$ | $1.273 \times 10^{-6}$ | 1.115 | 16.572 | 0.938 |
| 1.00 | 0.0585 | $1.591 \times 10^{-6}$ | $3.257 \times 10^{-7}$ | $1.273 \times 10^{-6}$ | 1.115 | 4.885 | 0.977 |
| 2.00 | 0.0585 | $2.571 \times 10^{-6}$ | $1.303 \times 10^{-6}$ | $1.273 \times 10^{-6}$ | 1.115 | 1.974 | 0.997 |
| 4.00 | 0.0585 | $6.518 \times 10^{-6}$ | $5.211 \times 10^{-6}$ | $1.273 \times 10^{-6}$ | 1.115 | 1.251 | 1.007 |
| *k sweep at* $g = 2$ | | | | | | | |
| $k$ | $\ell$ | $\Vert V_d^\top \hat{n}\Vert^2$ | $(g\ell^2/6)^2$ | floor | ratio (floor) | ratio (raw) | ratio (subtr.) |
| 20 | 0.0419 | $1.757 \times 10^{-6}$ | $3.439 \times 10^{-7}$ | $1.395 \times 10^{-6}$ | 1.190 | 5.108 | 1.050 |
| 40 | 0.0585 | $2.571 \times 10^{-6}$ | $1.303 \times 10^{-6}$ | $1.273 \times 10^{-6}$ | 1.115 | 1.974 | 0.997 |
| 80 | 0.0821 | $6.359 \times 10^{-6}$ | $5.045 \times 10^{-6}$ | $1.208 \times 10^{-6}$ | 1.076 | 1.261 | 1.021 |
| 160 | 0.1157 | $2.098 \times 10^{-5}$ | $1.991 \times 10^{-5}$ | $1.164 \times 10^{-6}$ | 1.043 | 1.054 | 0.995 |
| 320 | 0.1634 | $8.062 \times 10^{-5}$ | $7.916 \times 10^{-5}$ | $1.162 \times 10^{-6}$ | 1.045 | 1.018 | 1.004 |

Three things are read from the three ratio columns. First, the finite-sample floor: at $g = 0$ the rotation exceeds the leading-order $\ell^2\kappa^2/3k$ by 1.19, 1.12, 1.08, 1.04 and 1.05 at $k = 20$ to 320, an $O(1/k)$ excess that is within Monte Carlo error of unity at the largest $k$. Second, the raw ratio overstates the covariance term wherever the signal is comparable to that floor (16.6 at $g = 0.5$). Third, once the paired floor is subtracted the prediction $(g\ell^2/6)^2$ holds in every cell: 0.94–1.01 across the $g$ sweep, the lowest where the signal is only 6% of the floor, and 0.995–1.050 across a sixteenfold range of $k$.

The rotation rises 46-fold from $k = 20$ to $k = 320$. It is the only frame term in this supplement that grows with the neighborhood, and it grows as $\ell^4$.

Away from the edge the mean rotation matches the interior floor: 0.97–1.22 at $k \geq 40$ and 1.26–1.47 at $k = 20$, the same finite-$k$ excess as Table S5. (A probe at the pole, ang/cap = 0, has the same neighborhood at every azimuth, so that row carries only 3 effective samples and is omitted.) The rotation leaves the floor between edge/$\ell \approx 1.4$ and 1.0: it is 3.8 times the floor at edge/$\ell = 0.98$ ($k = 40$) and 9.7 times at 0.99 ($k = 160$), and 16–166 times at the ring nearest the edge. At edge/$\ell \approx 1$ the excess over the floor rises 3.1-fold from $k = 40$ to $k = 160$ for a 3.9-fold increase in $\ell^2$, so the growth with neighborhood size is close to, but slightly slower than, $\ell^2$.

Table S6: Boundary. Spherical cap of half-angle 0.35, $R = 5$, $F = 30$, $n_{\text{ref}} = 8000$, noiseless, $d = d_{\text{true}}$. Each cell places 200 probes at a fixed fraction of the cap half-angle from the pole and random azimuth, over 3 reference draws; "edge dist" is arc length to the cap edge. SE is that of the median. "interior" is the Eq. (S6) floor $\ell^2/(3kR^2)$ at the measured $\ell$; the ratio is the mean rotation over it.

| $k$ | ang/cap | edge dist | edge/$\ell$ | median | mean (SE of median) | interior | ratio (mean) |
|---|---|---|---|---|---|---|---|
| 20 | 0.30 | 1.2250 | 20.25 | $2.288 \times 10^{-6}$ | $3.582 \times 10^{-6}$ $(2.1 \times 10^{-7})$ | $2.440 \times 10^{-6}$ | 1.47 |
| | 0.60 | 0.7000 | 11.03 | $1.956 \times 10^{-6}$ | $3.370 \times 10^{-6}$ $(2.1 \times 10^{-7})$ | $2.684 \times 10^{-6}$ | 1.26 |
| | 0.80 | 0.3500 | 5.56 | $2.047 \times 10^{-6}$ | $3.617 \times 10^{-6}$ $(2.5 \times 10^{-7})$ | $2.646 \times 10^{-6}$ | 1.37 |
| | 0.90 | 0.1750 | 2.79 | $2.200 \times 10^{-6}$ | $3.476 \times 10^{-6}$ $(2.0 \times 10^{-7})$ | $2.622 \times 10^{-6}$ | 1.33 |
| | 0.95 | 0.0875 | 1.38 | $2.295 \times 10^{-6}$ | $3.778 \times 10^{-6}$ $(2.2 \times 10^{-7})$ | $2.690 \times 10^{-6}$ | 1.40 |
| | 0.99 | 0.0175 | 0.25 | $4.306 \times 10^{-5}$ | $4.945 \times 10^{-5}$ $(1.7 \times 10^{-6})$ | $3.169 \times 10^{-6}$ | 15.60 |
| 40 | 0.30 | 1.2250 | 14.17 | $1.628 \times 10^{-6}$ | $2.865 \times 10^{-6}$ $(1.8 \times 10^{-7})$ | $2.490 \times 10^{-6}$ | 1.15 |
| | 0.60 | 0.7000 | 7.81 | $1.726 \times 10^{-6}$ | $2.660 \times 10^{-6}$ $(1.4 \times 10^{-7})$ | $2.679 \times 10^{-6}$ | 0.99 |
| | 0.80 | 0.3500 | 4.00 | $1.854 \times 10^{-6}$ | $2.919 \times 10^{-6}$ $(1.6 \times 10^{-7})$ | $2.553 \times 10^{-6}$ | 1.14 |
| | 0.90 | 0.1750 | 1.98 | $1.842 \times 10^{-6}$ | $3.039 \times 10^{-6}$ $(1.8 \times 10^{-7})$ | $2.595 \times 10^{-6}$ | 1.17 |
| | 0.95 | 0.0875 | 0.98 | $7.701 \times 10^{-6}$ | $1.012 \times 10^{-5}$ $(4.7 \times 10^{-7})$ | $2.655 \times 10^{-6}$ | 3.81 |
| | 0.99 | 0.0175 | 0.18 | $9.415 \times 10^{-5}$ | $1.032 \times 10^{-4}$ $(2.4 \times 10^{-6})$ | $3.153 \times 10^{-6}$ | 32.73 |
| 80 | 0.30 | 1.2250 | 9.99 | $1.844 \times 10^{-6}$ | $2.847 \times 10^{-6}$ $(1.5 \times 10^{-7})$ | $2.505 \times 10^{-6}$ | 1.14 |
| | 0.60 | 0.7000 | 5.59 | $1.765 \times 10^{-6}$ | $2.661 \times 10^{-6}$ $(1.4 \times 10^{-7})$ | $2.617 \times 10^{-6}$ | 1.02 |
| | 0.80 | 0.3500 | 2.82 | $1.680 \times 10^{-6}$ | $2.596 \times 10^{-6}$ $(1.4 \times 10^{-7})$ | $2.564 \times 10^{-6}$ | 1.01 |
| | 0.90 | 0.1750 | 1.41 | $2.126 \times 10^{-6}$ | $3.142 \times 10^{-6}$ $(1.7 \times 10^{-7})$ | $2.583 \times 10^{-6}$ | 1.22 |
| | 0.95 | 0.0875 | 0.68 | $4.645 \times 10^{-5}$ | $4.936 \times 10^{-5}$ $(1.1 \times 10^{-6})$ | $2.782 \times 10^{-6}$ | 17.74 |
| | 0.99 | 0.0175 | 0.13 | $2.195 \times 10^{-4}$ | $2.249 \times 10^{-4}$ $(3.5 \times 10^{-6})$ | $3.128 \times 10^{-6}$ | 71.90 |
| 160 | 0.30 | 1.2250 | 7.02 | $2.024 \times 10^{-6}$ | $2.855 \times 10^{-6}$ $(1.5 \times 10^{-7})$ | $2.539 \times 10^{-6}$ | 1.12 |
| | 0.60 | 0.7000 | 4.02 | $1.830 \times 10^{-6}$ | $2.574 \times 10^{-6}$ $(1.3 \times 10^{-7})$ | $2.521 \times 10^{-6}$ | 1.02 |
| | 0.80 | 0.3500 | 2.01 | $1.700 \times 10^{-6}$ | $2.468 \times 10^{-6}$ $(1.2 \times 10^{-7})$ | $2.534 \times 10^{-6}$ | 0.97 |
| | 0.90 | 0.1750 | 0.99 | $2.363 \times 10^{-5}$ | $2.538 \times 10^{-5}$ $(7.0 \times 10^{-7})$ | $2.621 \times 10^{-6}$ | 9.68 |
| | 0.95 | 0.0875 | 0.47 | $1.931 \times 10^{-4}$ | $1.956 \times 10^{-4}$ $(2.6 \times 10^{-6})$ | $2.877 \times 10^{-6}$ | 67.99 |
| | 0.99 | 0.0175 | 0.09 | $5.115 \times 10^{-4}$ | $5.183 \times 10^{-4}$ $(5.1 \times 10^{-6})$ | $3.130 \times 10^{-6}$ | 165.59 |

# S8 Gate 3 — the anchor displacement law

## S8.1 Quadric patches: the sagitta and jitter laws

Table S7 is the direct test of Propositions 2–3 on quadric patches: the anchor displacement measured against the mean-curvature prediction, and the tangential jitter against $\ell/\sqrt{k}$.

Table S7: Proposition 2 (anchor displacement) and Proposition 3 (tangential jitter) on quadric patches, $F = 30$, $n_{\text{ref}} = 4000$, chart radius 1.00. Upper block: curvature sweep at $k = 40$, 400 trials/cell. Lower block: $k$ sweep at fixed isotropic curvature $\kappa_1 = \kappa_2 = 0.20$. Noiseless; $\ell^2$ is the mean of $\|u\|^2$ about the base point in the true chart. Ratio columns are measured/predicted, for $n_3$ in the upper block; — marks a prediction of zero, where a ratio is not a measurement.

| surface | $\ell^2$ | $\langle\mu, n_3\rangle$ | pred. | $\langle\mu, n_4\rangle$ | pred. | ratio |
|---|---|---|---|---|---|---|
| isotropic $\kappa_1$=$\kappa_2$=0.2, one normal | 0.00517 | 0.000517 | 0.000517 | −0.000000 | 0.000000 | 1.000 |
| minimal $\kappa_1$=$-\kappa_2$=0.2, one normal ($\vec{H} = 0$) | 0.00515 | 0.000002 | 0.000000 | 0.000000 | 0.000000 | — |
| anisotropic $\kappa_1$=0.4, $\kappa_2$=0, one normal | 0.00517 | 0.000519 | 0.000517 | −0.000000 | 0.000000 | 1.004 |
| isotropic $\kappa_1$=$\kappa_2$=0.5, one normal | 0.00507 | 0.001267 | 0.001267 | 0.000000 | 0.000000 | 1.000 |
| biaxial $\kappa_1$=0.2 on $n_3$, $\kappa_2$=−0.2 on $n_4$ | 0.00523 | 0.000261 | 0.000262 | −0.000262 | −0.000262 | 0.998 |
| biaxial $\kappa_1$=$\kappa_2$=0.2, two normals | 0.00500 | 0.000249 | 0.000250 | 0.000251 | 0.000250 | 0.995 |

| $k$ | $\ell^2$ | $\langle\mu, n_3\rangle$ | pred. | rms jitter | $\ell/\sqrt{k}$ | ratio (sagitta) | ratio (jitter) |
|---|---|---|---|---|---|---|---|
| 20 | 0.00265 | 0.000265 | 0.000265 | 0.01127 | 0.01150 | 1.000 | 0.979 |
| 40 | 0.00510 | 0.000510 | 0.000510 | 0.01155 | 0.01130 | 1.000 | 1.022 |
| 80 | 0.01008 | 0.001008 | 0.001008 | 0.01107 | 0.01122 | 1.000 | 0.986 |
| 160 | 0.02006 | 0.002006 | 0.002006 | 0.01132 | 0.01120 | 1.000 | 1.011 |
| 320 | 0.04016 | 0.004016 | 0.004016 | 0.01132 | 0.01120 | 1.000 | 1.010 |

Read the ratio columns with the construction in mind. On a one-normal patch with $\kappa_1 = \kappa_2$ the displacement along $n_3$ is $\frac{\kappa}{2}$ times the mean of $\|u\|^2$ over the same neighbours that define $\ell^2$, so the isotropic rows and the whole sagitta column of the lower block are 1 by construction and test only the arithmetic. The informative rows are the others. The anisotropic row (1.004) and the two biaxial rows (0.998, 0.995) require the neighborhood's second moment to be isotropic, $\mathbb{E}[u_1^2] = \mathbb{E}[u_2^2] = \ell^2/2$, and it is. The lower block's jitter column tests Proposition 3 directly, and sits at 0.979–1.022 at every $k$ from 20 to 320.

The upper block's standout row is *minimal*, meaning zero mean curvature at the vertex. There $\kappa_1 = -\kappa_2 = 0.2$ — the same curvature magnitude as the isotropic row directly above it — yet the predicted displacement is exactly zero and the measured one, $2 \times 10^{-6}$, is the finite-sample difference between $\mathbb{E}[u_1^2]$ and $\mathbb{E}[u_2^2]$. This is the trace-of-$\mathrm{I\!I}$ argument from Gate 3 of the main text made concrete: the two principal curvatures cancel in the sum that governs $\vec{H}$, so a surface that is locally just as curved as the isotropic case produces no anchor bias at all. The two biaxial rows make the complementary point: with curvature split across two normal directions $n_3, n_4$, each direction's displacement matches *its own* curvature component independently — $\langle\mu, n_3\rangle$ against $\ell^2\kappa_1/4$ and $\langle\mu, n_4\rangle$ against $\ell^2\kappa_2/4$ — which is the mechanism Gate 5's booking rule depends on.

In the lower block $\langle\mu, n_3\rangle$ grows sixteenfold with $k$, tracking $\ell^2$ (Proposition 2), while the rms jitter stays at 0.0111–0.0116, matching the $k$-invariant $\ell/\sqrt{k}$ of Proposition 3. With $\ell \propto k^{1/d}$ the jitter scales as $k^{1/d-1/2}$, an exponent that is negative for $d > 2$, positive at $d = 1$ and exactly zero at $d = 2$; these are two-dimensional patches, and the flat column is that exponent vanishing.

## S8.2 An inhomogeneous manifold: torus of revolution

On the sphere and the Clifford torus $\nabla \mathrm{II} = 0$ everywhere, and a quadric patch has $\nabla \mathrm{II} = 0$ at its vertex, so the term Proposition 2 neglects is structurally zero in all three. A torus of revolution removes that. With $R_c < 2r$ the mean curvature changes sign on the inner region, so the anchor displacement should *reverse* there. The same table carries gate 4: the alignment $a = |R_c \cos\theta + r|/\|p\|$ varies over the surface and passes through zero.

Table S8: Torus of revolution, $R_c = 3$, $r = 2$, $F = 30$, $k = 40$, 4000 probes, noiseless. Probes sit *at* their base points. The prediction is $-(\ell^2/4)(\kappa_1 + \kappa_2)$ along the outward normal, with $\ell^2$ the measured mean of the tangential extent over probes; this is 1.010, 1.016, 1.019 and 1.024 times the areal value at $n_{\mathrm{ref}} = 5000$, 10000, 20000, 40000, against the $k$-NN boundary factor $(k+1)/k = 1.025$. The ratio is the least-squares slope of per-probe displacement on per-probe prediction within the bin; its SE treats probes as independent and is a lower bound, since probes share reference points. Upper table: $n_{\mathrm{ref}} = 40000$, bin medians. Lower table: the slope at each $n_{\mathrm{ref}}$.

| $\theta$ bin | $\kappa_1{+}\kappa_2$ | disp. meas. | pred. | $a$ meas. | $a$ true | max $\lvert\Delta a\rvert$ | % degen. | ratio (SE) |
|---|---|---|---|---|---|---|---|---|
| 0.00–0.79 | 0.6919 | −0.00649 | −0.00668 | 0.976 | 0.976 | 0.009 | 0.0 | 0.988 (0.007) |
| 0.79–1.57 | 0.6064 | −0.00569 | −0.00585 | 0.761 | 0.761 | 0.016 | 0.0 | 0.996 (0.008) |
| 1.57–2.36 | 0.3470 | −0.00339 | −0.00335 | 0.321 | 0.319 | 0.035 | 13.7 | 0.999 (0.017) |
| 2.36–3.14 | −0.3129 | +0.00251 | +0.00302 | 0.559 | 0.577 | 0.067 | 4.7 | 0.974 (0.036) |
| 3.14–3.93 | −0.2394 | +0.00210 | +0.00231 | 0.459 | 0.455 | 0.052 | 7.7 | 0.973 (0.040) |
| 3.93–4.71 | 0.3436 | −0.00310 | −0.00332 | 0.317 | 0.314 | 0.024 | 15.4 | 0.975 (0.016) |
| 4.71–5.50 | 0.6075 | −0.00580 | −0.00587 | 0.763 | 0.763 | 0.015 | 0.0 | 1.014 (0.008) |
| 5.50–6.28 | 0.6916 | −0.00678 | −0.00668 | 0.975 | 0.975 | 0.006 | 0.0 | 1.022 (0.007) |

| $\theta$ bin | ratio, $n_{\mathrm{ref}} = 5000$ | 10000 | 20000 | 40000 |
|---|---|---|---|---|
| 0.00–0.79 | 0.994 (0.006) | 1.006 (0.007) | 1.006 (0.007) | 0.988 (0.007) |
| 0.79–1.57 | 1.065 (0.009) | 1.021 (0.007) | 1.037 (0.008) | 0.996 (0.008) |
| 1.57–2.36 | 1.064 (0.014) | 1.003 (0.016) | 1.006 (0.016) | 0.999 (0.017) |
| 2.36–3.14 | 0.909 (0.041) | 1.099 (0.041) | 0.995 (0.046) | 0.974 (0.036) |
| 3.14–3.93 | 1.095 (0.036) | 1.068 (0.038) | 0.966 (0.036) | 0.973 (0.040) |
| 3.93–4.71 | 1.015 (0.014) | 0.998 (0.015) | 0.965 (0.014) | 0.975 (0.016) |
| 4.71–5.50 | 1.024 (0.008) | 1.018 (0.008) | 1.005 (0.007) | 1.014 (0.008) |
| 5.50–6.28 | 1.002 (0.006) | 0.995 (0.007) | 1.006 (0.007) | 1.022 (0.007) |

The sign of the displacement reverses with $\kappa_1{+}\kappa_2$ in the inner bins at every $n_{\mathrm{ref}}$, as Proposition 2 requires. At $n_{\mathrm{ref}} = 40000$ the slopes are 0.973–1.022; the four outer bins average 1.005, and the two inner bins, where $\ell|\kappa|$ is largest, sit within one SE of 1. Bins at $\theta$ and $2\pi - \theta$ are geometrically equivalent, yet the 0–0.79 and 5.50–6.28 pair differs by 0.034 at $n_{\mathrm{ref}} = 40000$, several printed SE; the SE is a lower bound, and pooling mirror bins is the better summary (1.005, 1.005, 0.986, 0.974 from outer to inner, inverse-variance weighted). The largest departures at $n_{\mathrm{ref}} = 5000$ (1.065 and 1.064 in the bins from 0.79 to 2.36) have fallen to 1.021 and 1.003 by 10000: a neglected higher-order term shrinking with $\ell$, as it should. With the areal $\ell^2$ in place of the measured one the outer bins sit 2.5–3% high at every $n_{\mathrm{ref}}$ instead — an $\ell$-independent offset, which is the boundary factor $(k+1)/k$, and which the measured-to-areal ratio in the caption approaches as $\ell$ falls and the tangent-projection shrinkage vanishes.

The median measured alignment matches the analytic value to within 0.003 in every bin outside the inner region and within 0.02 inside it; per-probe errors reach 0.07 in the inner bins. Degeneracy is flagged in the bins containing the zeros of $a$ ($\cos\theta = -2/3$), at 14–15% of probes, and at 5–8%

in the inner bins beside them.

**A measurement confound, recorded because it is easy to reproduce.** If the probes are stepped an arc length $s$ along the manifold and the anchor displacement is referred to the *pre-step* base point rather than to the probe's own position, the probe's own normal offset $\kappa_n s^2/2$ contaminates the measurement. At $\theta = \pi$ and $s = 0.10$ that offset is about $-0.005$, against a predicted inner-bin sagitta of $+0.002$ to $+0.003$ at $n_{\mathrm{ref}} = 40000$. The contaminant does not shrink with $\ell$, so the apparent ratio *diverges* as $n_{\mathrm{ref}}$ rises — the signature of an $\ell$-independent contaminant rather than of a neglected higher-order term. Referring the displacement to the probe's own position, as here, removes it.

### S8.3 Free-parameter dependence

Table S9 tests the closed-form scalings of the main text on a sphere, and tests two further channels — the magnitude of a probe's own displacement, and reference noise — that might be expected to drive the anchor displacement but do not.

Table S9: Anchor bias against the free parameters. Defaults $F = 30$, $R = 5$, $n_{\mathrm{ref}} = 4000$, $k = 40$, full sphere, noiseless; 250 trials per cell, the same draws in every cell. $\ell$ is measured tangentially about the neighborhood centroid with the $(k-1)$ normalization. ratio $= \delta/(\ell^2/2R)$; ratio2 $= \delta/[(\ell^2/2R)(1 + \ell^2/3R^2)]$ (Eq. S3), with its Monte Carlo SE.

| sweep | value | $\ell$ | $\delta$ | $\ell^2/2R$ | jitter | $\ell/\sqrt{k}$ | ratio | ratio2 (SE) |
|---|---|---|---|---|---|---|---|---|
| $n_{\mathrm{ref}}$ | 500 | 1.9446 | 0.39997 | 0.37816 | 0.30657 | 0.30747 | 1.058 | 1.007 (0.011) |
| | 1000 | 1.4250 | 0.20852 | 0.20305 | 0.21440 | 0.22531 | 1.027 | 1.000 (0.012) |
| | 2000 | 1.0065 | 0.10276 | 0.10130 | 0.15948 | 0.15914 | 1.014 | 1.001 (0.011) |
| | 4000 | 0.7116 | 0.05088 | 0.05064 | 0.10712 | 0.11252 | 1.005 | 0.998 (0.011) |
| | 8000 | 0.4996 | 0.02507 | 0.02496 | 0.08038 | 0.07899 | 1.004 | 1.001 (0.011) |
| | 16000 | 0.3590 | 0.01292 | 0.01289 | 0.05742 | 0.05676 | 1.002 | 1.001 (0.012) |
| $R$ | 3.0 | 0.4270 | 0.03053 | 0.03038 | 0.06427 | 0.06751 | 1.005 | 0.998 (0.011) |
| | 5.0 | 0.7116 | 0.05088 | 0.05064 | 0.10712 | 0.11252 | 1.005 | 0.998 (0.011) |
| | 8.0 | 1.1386 | 0.08140 | 0.08102 | 0.17140 | 0.18003 | 1.005 | 0.998 (0.011) |
| | 12.0 | 1.7079 | 0.12210 | 0.12154 | 0.25709 | 0.27004 | 1.005 | 0.998 (0.011) |
| | 20.0 | 2.8465 | 0.20350 | 0.20256 | 0.42849 | 0.45006 | 1.005 | 0.998 (0.011) |
| cap angle | 0.30 | 0.1065 | 0.00113 | 0.00113 | 0.01691 | 0.01683 | 1.000 | 1.000 (0.011) |
| | 0.60 | 0.2105 | 0.00443 | 0.00443 | 0.03343 | 0.03328 | 1.001 | 1.000 (0.011) |
| | 1.00 | 0.3413 | 0.01167 | 0.01165 | 0.05421 | 0.05396 | 1.002 | 1.000 (0.011) |
| | 1.50 | 0.4848 | 0.02359 | 0.02350 | 0.07701 | 0.07666 | 1.003 | 1.000 (0.011) |
| | $\pi$ | 0.7116 | 0.05088 | 0.05064 | 0.10712 | 0.11252 | 1.005 | 0.998 (0.011) |
| gain offset | $-1.0$ to $+2.0$ (8) | 0.7116 | 0.05088 | 0.05064 | 0.10712 | 0.11252 | 1.005 | 0.998 (0.011) |
| noise $\sigma$ | 0.00 | 0.7116 | 0.05088 | 0.05064 | 0.10712 | 0.11252 | 1.005 | 0.998 (0.011) |
| | 0.01 | 0.7032 | 0.04975 | 0.04945 | 0.11072 | 0.11118 | 1.006 | 1.000 (0.011) |
| | 0.05 | 0.7041 | 0.04969 | 0.04957 | 0.11059 | 0.11132 | 1.002 | 0.996 (0.015) |
| | 0.10 | 0.7066 | 0.04978 | 0.04992 | 0.11346 | 0.11172 | 0.997 | 0.990 (0.023) |
| | 0.20 | 0.7368 | 0.05414 | 0.05428 | 0.11854 | 0.11649 | 0.997 | 0.990 (0.037) |

With the tangential term of Eq. (S3) included, ratio2 is 0.998–1.007 in every noiseless cell, within one SE. The uncorrected ratio runs from 1.058 at $n_{\mathrm{ref}} = 500$ to 1.002 at 16000, shrinking with $\ell/R$, so the excess is that next-order term and not a bias. The $n_{\mathrm{ref}}$ block gives $\delta \propto n_{\mathrm{ref}}^{-1}$ (a 31-fold fall over a 32-fold range) and jitter $\propto n_{\mathrm{ref}}^{-1/2}$, within 5% of $\ell/\sqrt{k}$ in every row.

The $R$ block repeats one row at every radius up to scale: $\delta/R = 0.0102$ and both ratios are identical. On a closed sphere at fixed $n_{\mathrm{ref}}$ and $k$ the whole configuration scales with $R$, so $\delta \propto R$ is dimensional. A flatter reference manifold is worse only against a fixed absolute probe magnitude or noise; on a patch of fixed area $\ell$ would not grow with $R$ and $\delta$ would fall. Cap angle acts through sampled area: ratio2 is 1.000 across a sevenfold range in $\ell$. All eight gain offsets give the default cell to every printed digit, because radial probes on an origin-centered sphere leave the $k$-NN ordering exactly unchanged. Reference noise up to $\sigma = 0.2$ inflates the measured $\ell$ but leaves ratio2 at 0.990–1.000, within its SE: noise is a fluctuation, not a bias channel.

## S8.4 Anisotropy and the anchor

Proposition 2 assumes isotropic noise. This sweep tests it directly.

Table S10: Proposition 2 under anisotropy, $\Sigma = \sigma^2(I + \kappa\, ww^\top)$. Anchor displacement measured directly at a single base point on a sphere, $R = 5$, $F = 30$, $n_{\mathrm{ref}} = 4000$, $k = 40$, $\sigma = 0.05$, 2000 trials per cell, the same draws for every role and $\kappa$. Ratio columns as in Table S9.

| $w$ role | $\kappa$ | $\sigma_w$ | $\ell$ | $\delta$ | $\ell^2/2R$ | ratio | ratio2 (SE) |
|---|---|---|---|---|---|---|---|
| isotropic | 0 | 0.050 | 0.7130 | 0.05118 | 0.05084 | 1.007 | 1.000 (0.005) |
| radial | 25 | 0.255 | 0.7164 | 0.03871 | 0.05132 | 0.754 | 0.749 (0.017) |
| | 100 | 0.502 | 0.7442 | 0.02399 | 0.05538 | 0.433 | 0.430 (0.024) |
| | 400 | 1.001 | 0.8437 | 0.01720 | 0.07118 | 0.242 | 0.239 (0.026) |
| tangent | 25 | 0.255 | 0.7119 | 0.05746 | 0.05068 | 1.134 | 1.126 (0.006) |
| | 100 | 0.502 | 0.7097 | 0.07508 | 0.05037 | 1.491 | 1.481 (0.007) |
| | 400 | 1.001 | 0.7132 | 0.13234 | 0.05086 | 2.602 | 2.585 (0.011) |
| ambient | 25 | 0.255 | 0.7159 | 0.05160 | 0.05125 | 1.007 | 1.000 (0.005) |
| | 100 | 0.502 | 0.7439 | 0.05584 | 0.05534 | 1.009 | 1.002 (0.005) |
| | 400 | 1.001 | 0.8417 | 0.07162 | 0.07085 | 1.011 | 1.002 (0.004) |

Isotropic noise leaves Proposition 2 exact to its next order (ratio2 $1.000 \pm 0.005$). Radial anisotropy erases the sagitta: $\delta$ falls threefold, from 0.0512 to 0.0172, *while $\ell$ rises*, so the effect is in the displacement itself and not in the convention for measuring $\ell$; ratio2 falls to 0.749, 0.430 and 0.239. Tangential anisotropy inflates it 2.6-fold with $\ell$ flat: tangentially displaced points from further around the sphere become selectable, so the selected set spans more arc than the measured $\ell$ reports. Ambient anisotropy is inert (ratio2 1.000–1.002) even as it inflates $\ell$ by 18%. Proposition 2's insensitivity to the noise scale, established under isotropy, does not extend to noise concentrated along the radial or tangential directions.

## S9 Gate 4 — anisotropic noise

Anisotropy imposed as $\Sigma = \sigma^2(I + \kappa\, ww^\top)$ on an origin-centered spherical cap small enough that the local frame is near-constant, with $w$ placed in each of three geometric roles. The imposed change is $(G, T, O) = (0.5, 0, 0.5)$ throughout, so departures from those values are the effect of the noise geometry alone.

Table S11: Anisotropic noise by direction. Cap half-angle 0.30, $R = 5$, $F = 30$, $d = 2$, $n_{\text{ref}} = 1500$, $k = 100$, 300 probes, $\sigma = 0.01$, imposed $\nu = 1$, $O_\star = 0.5$; medians, the same draws in every cell. $\sigma_w = \sigma\sqrt{1+\kappa}$ is the noise scale along $w$ and $\ell$ the tangential neighborhood extent.

| $w$ role | $\kappa$ | $\sigma_w/\ell$ | $G$ | $T$ | $O$ | alignment | % degen. |
|---|---|---|---|---|---|---|---|
| gain axis | 0 | 0.035 | 0.4987 | 0.0014 | 0.4925 | 1.0000 | 0.0 |
| | 100 | 0.302 | 0.4732 | 0.0017 | 0.5246 | 0.9960 | 0.0 |
| | 400 | 0.529 | 0.4095 | 0.0038 | 0.5849 | 0.9748 | 0.0 |
| | 900 | 0.729 | 0.3355 | 0.0086 | 0.6519 | 0.9183 | 0.0 |
| | 1600 | 0.924 | 0.2681 | 0.0156 | 0.7125 | 0.8052 | 0.3 |
| | 6400 | 1.557 | **0.1876** | 0.0168 | **0.7992** | **0.4782** | 3.7 |
| tangent | 0 | 0.035 | 0.4987 | 0.0014 | 0.4925 | 1.0000 | 0.0 |
| | 100 | 0.351 | 0.4966 | 0.0017 | 0.4935 | 0.9999 | 0.0 |
| | 400 | 0.685 | 0.4919 | 0.0018 | 0.4953 | 0.9993 | 0.0 |
| | 900 | 0.994 | 0.4900 | 0.0022 | 0.4942 | 0.9980 | 0.0 |
| | 1600 | 1.293 | 0.4864 | 0.0025 | 0.4952 | 0.9969 | 0.0 |
| | 6400 | 2.272 | 0.4760 | 0.0046 | 0.4924 | 0.9908 | 0.0 |
| ambient | 0 | 0.035 | 0.4987 | 0.0014 | 0.4925 | 1.0000 | 0.0 |
| | 100 | 0.347 | 0.4968 | 0.0014 | 0.4946 | 1.0000 | 0.0 |
| | 400 | 0.649 | 0.4980 | 0.0014 | 0.4982 | 1.0000 | 0.0 |
| | 900 | 0.890 | 0.4998 | 0.0023 | 0.4963 | 1.0000 | 0.0 |
| | 1600 | 1.080 | 0.5005 | 0.0036 | 0.4924 | 0.9998 | 0.0 |
| | 6400 | 1.679 | 0.5011 | 0.0067 | 0.4856 | 0.9972 | 0.0 |

Tangential anisotropy leaves $O$ within 0.003 and $G$ within 0.023 of their isotropic values out to $\sigma_w/\ell = 2.272$, with $T \leq 0.005$; ambient anisotropy leaves both within 0.007 out to $\sigma_w/\ell = 1.679$. Along the gain axis the same ratios produce a monotone transfer of energy from $G$ to $O$ with no threshold. At $\sigma_w/\ell = 0.529$ the recovered $G$ has already fallen by 18%; at 0.924 it is 0.268 with $O = 0.713$; at 1.557 it retains 38% of its isotropic value, $O$ has risen to 0.799, the alignment is 0.478, and 3.7% of probes are flagged degenerate.

**Why the directional quadratic forms are not the correction.** Substituting $\hat{n}_g^\top \Sigma \hat{n}_g$, $\operatorname{tr}(P_{\text{res}} \Sigma P_{\text{res}})$ and $\operatorname{tr}(V_d^\top \Sigma V_d)$ into the noise-augmented form of Proposition 1 predicts, at $\kappa = 6400$, $O \to 0.30$ for tangential anisotropy and $O \to 0.68$ for ambient. Neither occurs: the measured values are 0.492 and 0.486, against the isotropic 0.492. The substitution treats the noise as added to the probe against a fixed anchor and frame, whereas both are estimated from the same noisy cloud and absorb the shared component.

**The alignment tolerance does not protect.** At $\kappa = 1600$ the alignment is still 0.81 — far above any threshold one would set to flag a non-existent gain axis — while $G$ has already lost 46% of its isotropic value. Alignment must be read as a graded quantity for this failure mode to be visible at all.

### S9.1 Anisotropy and the frame (gates 2 and 6)

Table S12: Gates 2 and 6 under anisotropy. Spherical cap of half-angle 0.25, $R = 5$, $F = 30$, $n_{\mathrm{ref}} = 4000$, $k = 40$, $\sigma = 0.02$, 200 probes; medians, the same draws in every cell. $\tau^2$ is the tangential energy of $r$; the ratio is $\tau^2$ over $\ell^2/k$ measured on the same neighborhoods.

| $w$ role | $\kappa$ | $\sigma_w$ | $\|V_d^\top \hat{n}\|^2$ | vs. isotropic | $\tau^2$ | $\ell^2/k$ | ratio |
|---|---|---|---|---|---|---|---|
| isotropic | 0 | 0.020 | $2.175 \times 10^{-3}$ | 1.00 | 0.00031 | 0.00038 | 0.817 |
| radial | 25 | 0.102 | $9.850 \times 10^{-2}$ | 45.3 | 0.00041 | 0.00045 | 0.891 |
| | 100 | 0.201 | $5.381 \times 10^{-1}$ | 247.4 | 0.00066 | 0.00059 | 1.127 |
| | 400 | 0.400 | $7.225 \times 10^{-1}$ | 332.2 | 0.00086 | 0.00085 | 1.020 |
| tangent | 25 | 0.102 | $3.587 \times 10^{-3}$ | 1.65 | 0.00038 | 0.00039 | 0.961 |
| | 100 | 0.201 | $5.992 \times 10^{-3}$ | 2.75 | 0.00038 | 0.00041 | 0.918 |
| | 400 | 0.400 | $1.222 \times 10^{-2}$ | 5.62 | 0.00048 | 0.00045 | 1.083 |
| ambient | 25 | 0.102 | $1.933 \times 10^{-3}$ | 0.89 | 0.00047 | 0.00047 | 1.009 |
| | 100 | 0.201 | $1.340 \times 10^{-3}$ | 0.62 | 0.00085 | 0.00061 | 1.395 |
| | 400 | 0.400 | $9.675 \times 10^{-4}$ | 0.44 | 0.00102 | 0.00084 | 1.216 |

The gate-2 rotation term is strongly direction-sensitive. Radial anisotropy raises it 332-fold, to $7.225 \times 10^{-1}$, so the normal direction lies mostly *inside* the estimated tangent space. Tangential anisotropy raises it 5.6-fold. Ambient anisotropy lowers it to 0.44 of the isotropic value, since a captured ambient direction occupies a basis slot that would otherwise tilt toward the normal. The gate-6 term $\tau^2$ also moves with the anisotropy — 2.8-fold radially, 1.5-fold tangentially, 3.3-fold in the ambient role — but it moves with the $\ell^2/k$ of the same neighborhoods: the ratio is 0.82–1.13 in the radial and tangential roles, and reaches 1.40 only where ambient noise competes for a tangent slot. The observable conditioning check of gate 6 therefore survives anisotropy, provided $\ell$ is measured on the data rather than assumed.

# S10 Gate 5 — where the anchor displacement is booked

Table S13 is the pure on-manifold (geodesic) sweep on spheres of five radii and four neighborhood sizes. It isolates the absolute novel energy from the $O$ fraction, and compares the radial displacement with the sagitta.

Table S13: Pure on-manifold (geodesic) sweep on spheres of five radii and four neighborhood sizes, $F = 30$, $n_{\text{ref}} = 6000$, 300 probes/cell, arc length 0.30, ambient noise $\sigma = 0.01$ (noise floor $\sqrt{F-3}\,\sigma = 0.0520$). Every probe is a geodesic step, so the imposed $(G, T, O)$ is $(0, 1, 0)$ exactly and any radial or off-manifold energy in $r$ is the anchor's, not the probe's. Ratio columns, left to right: $\|r_{\text{rad}}\|$ over $\ell^2/2R$; the same over $(\ell^2/2R)(1 + \ell^2/3R^2)$, with the SE of the median; $\|r_{\text{novel}}\|$ over the noise floor.

| $R$ | $k$ | $\ell$ | $\|r\|$ | $\|r_{\text{rad}}\|$ | $\ell^2/2R$ | $\|r_{\text{novel}}\|$ | ratio | ratio2 (SE) | ratio (novel) |
|---|---|---|---|---|---|---|---|---|---|
| 3.0 | 20 | 0.247 | 0.0732 | 0.0107 | 0.0102 | 0.0525 | 1.053 | 1.050 (0.060) | 1.011 |
| 3.0 | 40 | 0.346 | 0.0735 | 0.0195 | 0.0200 | 0.0523 | 0.973 | 0.969 (0.040) | 1.007 |
| 3.0 | 80 | 0.492 | 0.0820 | 0.0405 | 0.0403 | 0.0520 | 1.006 | 0.997 (0.020) | 1.001 |
| 3.0 | 160 | 0.690 | 0.1090 | 0.0799 | 0.0793 | 0.0511 | 1.007 | 0.990 (0.011) | 0.983 |
| 5.0 | 20 | 0.413 | 0.0973 | 0.0159 | 0.0171 | 0.0525 | 0.929 | 0.927 (0.041) | 1.010 |
| 5.0 | 40 | 0.576 | 0.0954 | 0.0323 | 0.0332 | 0.0519 | 0.972 | 0.968 (0.025) | 0.999 |
| 5.0 | 80 | 0.811 | 0.1128 | 0.0657 | 0.0657 | 0.0519 | 1.000 | 0.991 (0.014) | 0.998 |
| 5.0 | 160 | 1.149 | 0.1641 | 0.1341 | 0.1321 | 0.0516 | 1.015 | 0.997 (0.008) | 0.992 |
| 8.0 | 20 | 0.662 | 0.1411 | 0.0268 | 0.0274 | 0.0523 | 0.981 | 0.979 (0.032) | 1.007 |
| 8.0 | 40 | 0.927 | 0.1414 | 0.0522 | 0.0538 | 0.0521 | 0.972 | 0.967 (0.020) | 1.002 |
| 8.0 | 80 | 1.301 | 0.1682 | 0.1070 | 0.1057 | 0.0511 | 1.012 | 1.003 (0.012) | 0.983 |
| 8.0 | 160 | 1.834 | 0.2576 | 0.2150 | 0.2102 | 0.0508 | 1.023 | 1.005 (0.007) | 0.978 |
| 12.0 | 20 | 0.975 | 0.1954 | 0.0393 | 0.0396 | 0.0528 | 0.991 | 0.989 (0.023) | 1.015 |
| 12.0 | 40 | 1.393 | 0.2089 | 0.0815 | 0.0808 | 0.0514 | 1.008 | 1.003 (0.016) | 0.989 |
| 12.0 | 80 | 1.967 | 0.2455 | 0.1605 | 0.1613 | 0.0515 | 0.995 | 0.987 (0.010) | 0.992 |
| 12.0 | 160 | 2.739 | 0.3708 | 0.3172 | 0.3126 | 0.0505 | 1.015 | 0.997 (0.006) | 0.972 |
| 20.0 | 20 | 1.646 | 0.2862 | 0.0662 | 0.0677 | 0.0533 | 0.977 | 0.975 (0.023) | 1.026 |
| 20.0 | 40 | 2.322 | 0.3471 | 0.1349 | 0.1348 | 0.0517 | 1.001 | 0.996 (0.013) | 0.994 |
| 20.0 | 80 | 3.256 | 0.4233 | 0.2650 | 0.2650 | 0.0515 | 1.000 | 0.991 (0.010) | 0.992 |
| 20.0 | 160 | 4.605 | 0.6241 | 0.5376 | 0.5301 | 0.0521 | 1.014 | 0.996 (0.007) | 1.002 |

Against Eq. (S3) the radial displacement is recovered in every cell: ratio2 averages 0.984, 0.981, 0.994 and 0.997 at $k$ = 20, 40, 80, 160, and no cell departs from 1 by more than 1.8 SE. The scatter at small $k$ is sampling error, since the bias is smallest there and Proposition 3's jitter is largest relative to it. At $k = 160$, where $\ell/R \approx 0.23$ at every radius, the uncorrected ratio runs 1.007–1.023; that excess is the tangent-projection term $\ell^2/3R^2 \approx 1.8\%$.

The result the table exists to establish is $\|r_{\text{novel}}\|$: 0.0505–0.0533 against a noise floor of 0.0520, with no trend in either $R$ or $k$. Curvature on a sphere produces real, radial anchor displacement but no off-manifold energy at all; the entire sagitta is absorbed by the gain axis, exactly as gate 5's booking rule requires when $|c| = 1$. The fraction $O$ nonetheless varies from cell to cell, entirely through its denominator: $\|r\|^2$ is the sagitta, the noise, and the tangential jitter $\tau^2$, which carries roughly 25–90% of $\|r\|^2$ (from the medians of the components). This is the noise floor the Clifford torus below is measured against.

## S10.1 The $|c| < 1$ case: Clifford torus

For a $d$-manifold whose linear span is $(d + 1)$-dimensional the normal space inside the span is one-dimensional, forcing $|c| = 1$; this covers the sphere, all quadrics in three-space, the torus of

revolution and the ellipsoid. Reaching $|c| < 1$ requires a linear span of dimension at least $d + 2$. The Clifford torus $p(u, v) = (R_1 \cos u, R_1 \sin u, R_2 \cos v, R_2 \sin v)$ is the simplest such geometry with alignment exactly 1 (so gate 4 is clean). It is intrinsically flat (so probes can be stepped exactly along it), and gives $c = -(1/\rho)\|\vec{H}\|^{-1}$, $\rho = \sqrt{R_1^2 + R_2^2}$.

Table S14: Clifford torus, $R_1 = 1$, $F = 30$, $n_{\text{ref}} = 8000$, 300 probes, $\sigma = 0.001$ (noise floor $\sqrt{F-3}\,\sigma = 0.0052$), arc 0.15; medians. Upper block: curvature alignment at $k = 40$, $R_2 = 1, 2, 3, 5$. Lower block: $k$ sweep at $R_2 = 3$. $\eta$ and $\delta$ use the areal $\ell^2$. Ratio columns: $\|r_{\text{novel}}\|$ over $\eta$, and over $\sqrt{\eta^2 + \text{floor}^2}$, since the noise adds in quadrature.

| $R_1$:$R_2$ | $c$ | $a$ | $\|r_{\text{rad}}\|$ | $\delta$ pred. | $\|r_{\text{novel}}\|$ | $\eta$ pred. | ratio | ratio (quad.) |
|---|---|---|---|---|---|---|---|---|
| 1:1 | −1.000 | 1.000 | 0.01097 | 0.01111 | 0.00530 | 0 (exactly) | — | 1.019 |
| 1:2 | −0.800 | 1.000 | 0.01379 | 0.01405 | 0.01197 | 0.01054 | 1.136 | 1.019 |
| 1:3 | −0.600 | 1.000 | 0.01463 | 0.01490 | 0.02028 | 0.01987 | 1.021 | 0.987 |
| 1:5 | −0.385 | 1.000 | 0.01501 | 0.01540 | 0.03758 | 0.03697 | 1.016 | 1.007 |
| *$k$ sweep at $R_1$:$R_2 = 1$:3; $\ell^2$ areal. On the sphere this quantity was flat.* | | | | | | | | |
| $k$ | $\ell^2$ | | | | $\|r_{\text{novel}}\|$ | $\eta$ pred. | ratio | ratio (quad.) |
| 20 | 0.04712 | | | | 0.01113 | 0.00993 | 1.121 | 0.993 |
| 40 | 0.09425 | | | | 0.02028 | 0.01987 | 1.021 | 0.987 |
| 80 | 0.18850 | | | | 0.04080 | 0.03974 | 1.027 | 1.018 |
| 160 | 0.37699 | | | | 0.08118 | 0.07948 | 1.021 | 1.019 |

At $|c| = 1$ the novel energy sits at the noise floor (1.019), reproducing Table S13. As the torus is made asymmetric it rises to track $\eta$, and in the $k$ sweep it doubles with each doubling of $k$, as $\eta \propto \ell^2 \propto k$ requires — against the sphere, where the same quantity was flat in $k$ at the noise floor. With the floor added in quadrature the ratio is 0.987–1.019 in every row. The radial component tracks $\delta$ to within 3%. The $k$ sweep should be read with the areal $\ell^2$ in mind: it is low by the $k$-NN boundary factor $(k + 1)/k$ (Table S8) and, because $R_1 = 1$, at large $k$ it also exceeds the tangentially projected extent. The two effects partly offset across the sweep, so the $c$ sweep at fixed $k = 40$ is the cleaner test of the booking rule.

### S10.2 Varying second fundamental form: circle × ellipse

The Clifford torus is intrinsically flat, so $\nabla \mathrm{II} = 0$ and Proposition 2's neglected term is structurally zero there. Replacing the second circle by an ellipse, $p(u, v) = (R_1 \cos u,\ R_1 \sin u,\ A \cos v,\ B \sin v)$, keeps the linear span at $d + 2$ while making the normal curvature $\kappa_e = AB/q^3$, $q = \sqrt{A^2 \sin^2 v + B^2 \cos^2 v}$, vary around the ellipse — and with it $\|\vec{H}\|$, the alignment, and $c$.

The ratio is 0.979–1.005 across an eightfold range in $\ell^2$, with no systematic drift, so the booking rule is not an artefact of the Clifford torus's flatness. At the latitudes where $|c| \to 1$ (excluded above) the prediction $\eta \to 0$.

**A binning artefact worth avoiding.** Because $\eta$ varies strongly with $v$, evaluating the prediction at a bin's median coordinate rather than per probe biases the ratio by tens of percent — an artefact of aggregating before predicting, not a property of the rule. Predict per probe and aggregate afterwards.

Table S15: Circle × ellipse, $R_1 = 1$, $A = 1$, $B = 3$, $F = 30$, $d = 2$, $k = 60$, 1500 probes, noiseless; probes with $|c| < 0.95$. $\eta$ is predicted *per probe* from that probe's own $v$, with the areal $\ell^2$. The middle columns are medians over probes; the ratio is the median of the per-probe ratio, not the ratio of those medians.

| $n_{\text{ref}}$ | $\ell^2$ | $\|r_{\text{novel}}\|$ | $\eta$ pred. | ratio |
|---|---|---|---|---|
| 5000 | 0.16038 | 0.02545 | 0.02540 | 1.005 |
| 10000 | 0.08019 | 0.01254 | 0.01270 | 1.004 |
| 20000 | 0.04009 | 0.00616 | 0.00635 | 0.979 |
| 40000 | 0.02005 | 0.00310 | 0.00318 | 0.987 |

## S11 Gate 6 — recovery of the off-manifold fraction

### S11.1 The first-order form and its tangential term

On a noiseless origin-centered sphere $\eta = 0$ identically, so the first-order form of the main text reduces to a $\delta$ term plus a $\tau^2$ term. Table S16 compares the measured error with that form, with and without its tangential term, with the exact Proposition 1 form at the predicted $\delta$ and $\tau^2$, and with an anchor model: for each probe the anchor is placed at $(R - \delta)\hat{e} + \bar{u}$, with $\bar{u} \sim N(0, \frac{\tau^2}{2} I_2)$ in the tangent plane (Propositions 2–3 and nothing else), and $O$ is computed with the exact sphere frame there.

Table S16: Sphere, $R = 5$, $F = 30$, $d = 2$, noiseless, $\nu = 1$, $O_\star = 0.5$, 400 probes × 5 seeds. $\delta = \ell^2/2R$ and $\tau^2 = \ell^2/k$ from the measured $\ell$; the exact form and the model use $\delta(1 + \ell^2/3R^2)$. $O - O_\star$ is the median and the mean over probes, averaged over seeds. The first four ratio columns divide the *mean* error by: the first-order $\delta$ term alone; the first-order form with its tangential term; the exact form; the model's mean (sd across seeds). The last divides the median error by the model's median.

| | | | | $O - O_\star$ | | ratio: mean error over | | | | ratio |
|---|---|---|---|---|---|---|---|---|---|---|
| $n_{\text{ref}}$ | $k$ | $\delta$ | $\tau^2$ | median | mean | $\delta$ only | 1st order | exact | model (sd) | med./model |
| 4000 | 40 | 0.05010 | 0.01252 | −0.039236 | −0.040127 | 1.133 | 0.963 | 1.009 | 1.012 (0.025) | 1.032 |
| 8000 | 40 | 0.02503 | 0.00626 | −0.020023 | −0.020568 | 1.162 | 0.988 | 1.011 | 1.009 (0.011) | 1.027 |
| 16000 | 40 | 0.01266 | 0.00317 | −0.010216 | −0.010509 | 1.174 | 0.997 | 1.009 | 1.009 (0.013) | 1.024 |
| 16000 | 20 | 0.00648 | 0.00324 | −0.005793 | −0.006201 | 1.353 | 1.000 | 1.008 | 1.007 (0.017) | 1.016 |

Compared mean to mean, the first-order form with its tangential term converges to the measured error as $\delta$ shrinks: 0.963, 0.988, 0.997, 1.000 down the table. Without the tangential term the ratio stays at 1.133–1.174 at $k = 40$ and reaches 1.353 at $k = 20$. The $n_{\text{ref}}$ rows cannot by themselves show that the omitted term is the tangential one, because $\tau^2/\delta = 0.25$ in all three — both scale as $1/n_{\text{ref}}$ at fixed $k$ — so the evidence is the $k = 20$ row, where $\tau^2/\delta$ doubles to 0.50 and so does the shortfall. The exact form and the anchor model account for the mean error to about 1% in every cell (1.008–1.011 and 1.007–1.012): Propositions 2 and 3 are sufficient. Medians compared with the model's medians agree less well (1.016–1.032), because the model's per-probe jitter distribution only approximates the real one.

### S11.2 The full recovery sweep

Table S17 is the full sweep underlying the gate 6 discussion: recovered $\widehat{O}$ against imposed $O_\star$, swept over neighborhood size, gain sign and imposed magnitude, with a final block on a finer $O_\star$ grid in the one ill-conditioned condition. The oracle arm supplies the gate 2 estimator bound quoted in the main text.

$O_\star = 0$ occurs throughout, so no ratio column is given — a ratio against zero is not a number. Read the columns as comparisons instead, each isolating a different source of error.

- $O_\star$ is the imposed, ground-truth off-manifold fraction.
- $\widehat{O}$ is what the full estimator reports, with anchor, frame and gain axis estimated from a finite, noisy neighborhood — the number an analysis of real data would produce.
- $O_{\text{orac}}$ replaces the estimated frame with the generating manifold's exact geometry at the same anchor. $\widehat{O}$ against $O_{\text{orac}}$ isolates frame-estimation error, gate 2's contribution.

- $O_{\rm pred} = (b^2 + (F-3)\sigma^2)/[(g+\delta)^2 + b^2 + (F-3)\sigma^2 + \ell^2/k + \sigma^2]$, with $g = \pm\nu\sqrt{1-O_\star}$, $b = \nu\sqrt{O_\star}$ and $\delta = (\ell^2/2R)(1+\ell^2/3R^2)$, is the closed form evaluated at the *mean* jitter. $O_{\rm pred}$ against $O_\star$ is anchor bias with a perfect estimator.

- $O_{\rm model}$ is the median of the per-probe anchor model of Table S16, with anchor noise $\sigma/\sqrt{k}$ added. Unlike $O_{\rm pred}$ it has the full per-probe distribution, so it is the like-for-like comparison for a median.

- $C_6 = \text{median}\,(k\|r\|^2/\ell^2)$ is the observable conditioning index of gate 6: $\|r\|^2 \approx (g+\delta)^2 + b^2 + \ell^2/k$ plus noise, so $C_6 - 1 \gg 1$ is Eq. (25) of the main text.

Table S17: Recovered $\widehat{O}$ against imposed $O_\star$. Sphere, $F = 30$, $R = 5$, $d = 2$, $n_{\rm ref} = 4000$, $\sigma = 0.01$ in all $F$ dimensions, 300 probes per cell; medians. Swept over $k \in \{40, 160\}$, gain sign, and imposed displacement magnitude $\nu \in \{0.30, 1.00\}$. $\ell$ is the median tangential extent, $\delta = (\ell^2/2R)(1+\ell^2/3R^2)$. $G_{\rm rec} + T_{\rm rec} + \widehat{O} = 1$ per probe; medians need not sum to one. The final block repeats one condition on a finer $O_\star$ grid with independent draws.

| $O_\star$ | $\widehat{O}$ | $O_{\rm orac}$ | $O_{\rm pred}$ | $O_{\rm model}$ | $G_{\rm rec}$ | $T_{\rm rec}$ | $\|r\|$ | $\ell$ | $\delta$ | $C_6$ |
|---|---|---|---|---|---|---|---|---|---|---|
| *gain amplifying, $k = 40$, $\nu = 0.30$* | | | | | | | | | | |
| 0.00 | 0.020 | 0.020 | 0.020 | 0.020 | 0.905 | 0.076 | 0.371 | 0.706 | 0.050 | 11.0 |
| 0.25 | 0.190 | 0.190 | 0.188 | 0.191 | 0.738 | 0.068 | 0.363 | 0.710 | 0.051 | 10.6 |
| 0.50 | 0.374 | 0.374 | 0.371 | 0.372 | 0.541 | 0.071 | 0.357 | 0.701 | 0.049 | 10.5 |
| 0.75 | 0.580 | 0.580 | 0.573 | 0.580 | 0.329 | 0.076 | 0.350 | 0.703 | 0.050 | 9.9 |
| 1.00 | 0.871 | 0.872 | 0.860 | 0.891 | 0.023 | 0.104 | 0.327 | 0.706 | 0.050 | 8.8 |
| *gain amplifying, $k = 40$, $\nu = 1.00$* | | | | | | | | | | |
| 0.00 | 0.003 | 0.002 | 0.002 | 0.003 | 0.988 | 0.010 | 1.058 | 0.709 | 0.051 | 89.1 |
| 0.25 | 0.226 | 0.227 | 0.229 | 0.227 | 0.762 | 0.009 | 1.049 | 0.709 | 0.051 | 87.7 |
| 0.50 | 0.461 | 0.461 | 0.463 | 0.463 | 0.525 | 0.011 | 1.045 | 0.699 | 0.049 | 89.0 |
| 0.75 | 0.705 | 0.705 | 0.705 | 0.706 | 0.282 | 0.010 | 1.034 | 0.706 | 0.050 | 85.9 |
| 1.00 | 0.987 | 0.987 | 0.985 | 0.989 | 0.003 | 0.010 | 1.011 | 0.703 | 0.050 | 82.4 |
| *gain amplifying, $k = 160$, $\nu = 0.30$* | | | | | | | | | | |
| 0.00 | 0.010 | 0.010 | 0.010 | 0.010 | 0.951 | 0.038 | 0.517 | 1.405 | 0.203 | 21.8 |
| 0.25 | 0.099 | 0.099 | 0.101 | 0.101 | 0.855 | 0.042 | 0.503 | 1.401 | 0.201 | 20.5 |
| 0.50 | 0.205 | 0.205 | 0.207 | 0.209 | 0.742 | 0.049 | 0.484 | 1.398 | 0.200 | 19.0 |
| 0.75 | 0.335 | 0.335 | 0.339 | 0.341 | 0.598 | 0.056 | 0.457 | 1.405 | 0.203 | 16.9 |
| 1.00 | 0.642 | 0.642 | 0.639 | 0.651 | 0.270 | 0.068 | 0.381 | 1.397 | 0.200 | 11.9 |
| *gain amplifying, $k = 160$, $\nu = 1.00$* | | | | | | | | | | |
| 0.00 | 0.002 | 0.002 | 0.002 | 0.002 | 0.990 | 0.008 | 1.209 | 1.394 | 0.199 | 120.1 |
| 0.25 | 0.179 | 0.179 | 0.181 | 0.180 | 0.811 | 0.008 | 1.185 | 1.393 | 0.199 | 116.0 |
| 0.50 | 0.376 | 0.376 | 0.376 | 0.377 | 0.613 | 0.008 | 1.156 | 1.392 | 0.199 | 110.5 |
| 0.75 | 0.599 | 0.599 | 0.599 | 0.599 | 0.389 | 0.008 | 1.122 | 1.400 | 0.201 | 102.4 |
| 1.00 | 0.951 | 0.951 | 0.950 | 0.953 | 0.038 | 0.009 | 1.029 | 1.399 | 0.201 | 86.4 |
| *gain suppressive, $k = 40$, $\nu = 0.30$* | | | | | | | | | | |
| 0.00 | 0.034 | 0.034 | 0.035 | 0.034 | 0.839 | 0.122 | 0.274 | 0.713 | 0.051 | 6.1 |
| 0.25 | 0.312 | 0.312 | 0.307 | 0.319 | 0.559 | 0.114 | 0.282 | 0.694 | 0.048 | 6.9 |
| 0.50 | 0.565 | 0.565 | 0.551 | 0.576 | 0.309 | 0.111 | 0.289 | 0.703 | 0.050 | 7.0 |
| 0.75 | 0.770 | 0.770 | 0.757 | 0.792 | 0.108 | 0.123 | 0.304 | 0.703 | 0.050 | 7.7 |
| 1.00 | 0.874 | 0.875 | 0.861 | 0.899 | 0.021 | 0.101 | 0.326 | 0.705 | 0.050 | 8.6 |
| *gain suppressive, $k = 40$, $\nu = 1.00$* | | | | | | | | | | |
| 0.00 | 0.003 | 0.003 | 0.003 | 0.003 | 0.988 | 0.010 | 0.958 | 0.704 | 0.050 | 73.9 |
| 0.25 | 0.272 | 0.272 | 0.271 | 0.271 | 0.715 | 0.011 | 0.965 | 0.701 | 0.050 | 76.5 |
| 0.50 | 0.530 | 0.530 | 0.531 | 0.531 | 0.456 | 0.010 | 0.973 | 0.703 | 0.050 | 77.3 |

*continued on next page*

*Table S17 continued*

| $O_\star$ | $\widehat{O}$ | $O_{\mathrm{orac}}$ | $O_{\mathrm{pred}}$ | $O_{\mathrm{model}}$ | $G_{\mathrm{rec}}$ | $T_{\mathrm{rec}}$ | $\lVert r\rVert$ | $\ell$ | $\delta$ | $C_6$ |
|---|---|---|---|---|---|---|---|---|---|---|
| 0.75 | 0.779 | 0.779 | 0.778 | 0.781 | 0.207 | 0.010 | 0.982 | 0.706 | 0.050 | 77.5 |
| 1.00 | 0.990 | 0.990 | 0.986 | 0.989 | 0.002 | 0.007 | 1.009 | 0.696 | 0.049 | 83.4 |
| *gain suppressive, $k = 160$, $\nu = 0.30$* | | | | | | | | | | |
| 0.00 | 0.110 | 0.109 | 0.108 | 0.119 | 0.413 | 0.445 | 0.153 | 1.394 | 0.199 | 1.9 |
| 0.25 | 0.645 | 0.645 | 0.614 | 0.666 | 0.088 | 0.245 | 0.199 | 1.396 | 0.200 | 3.3 |
| 0.50 | 0.826 | 0.825 | 0.794 | 0.843 | 0.007 | 0.166 | 0.240 | 1.388 | 0.198 | 4.9 |
| 0.75 | 0.843 | 0.843 | 0.826 | 0.862 | 0.029 | 0.115 | 0.289 | 1.397 | 0.200 | 6.9 |
| 1.00 | 0.648 | 0.648 | 0.638 | 0.656 | 0.273 | 0.063 | 0.381 | 1.398 | 0.200 | 11.9 |
| *gain suppressive, $k = 160$, $\nu = 1.00$* | | | | | | | | | | |
| 0.00 | 0.004 | 0.004 | 0.004 | 0.004 | 0.984 | 0.012 | 0.812 | 1.402 | 0.202 | 53.4 |
| 0.25 | 0.356 | 0.356 | 0.357 | 0.358 | 0.625 | 0.013 | 0.841 | 1.399 | 0.201 | 58.1 |
| 0.50 | 0.650 | 0.650 | 0.651 | 0.651 | 0.334 | 0.011 | 0.881 | 1.396 | 0.200 | 64.2 |
| 0.75 | 0.880 | 0.881 | 0.880 | 0.881 | 0.106 | 0.010 | 0.924 | 1.392 | 0.199 | 70.4 |
| 1.00 | 0.951 | 0.951 | 0.950 | 0.953 | 0.038 | 0.008 | 1.029 | 1.399 | 0.201 | 86.5 |
| *gain suppressive, $k = 160$, $\nu = 0.30$, fine grid* | | | | | | | | | | |
| 0.00 | 0.117 | 0.116 | 0.107 | 0.120 | 0.467 | 0.392 | 0.150 | 1.391 | 0.199 | 1.9 |
| 0.05 | 0.260 | 0.261 | 0.254 | 0.270 | 0.343 | 0.371 | 0.164 | 1.387 | 0.197 | 2.2 |
| 0.10 | 0.391 | 0.391 | 0.373 | 0.410 | 0.254 | 0.323 | 0.174 | 1.389 | 0.198 | 2.6 |
| 0.15 | 0.504 | 0.505 | 0.469 | 0.510 | 0.186 | 0.283 | 0.180 | 1.389 | 0.198 | 2.7 |
| 0.20 | 0.597 | 0.597 | 0.548 | 0.604 | 0.133 | 0.245 | 0.191 | 1.392 | 0.199 | 3.0 |
| 0.25 | 0.646 | 0.645 | 0.614 | 0.660 | 0.086 | 0.237 | 0.197 | 1.397 | 0.200 | 3.2 |
| 0.30 | 0.712 | 0.713 | 0.668 | 0.727 | 0.058 | 0.217 | 0.202 | 1.404 | 0.202 | 3.4 |
| 0.35 | 0.734 | 0.735 | 0.709 | 0.747 | 0.037 | 0.207 | 0.219 | 1.395 | 0.200 | 4.0 |
| 0.40 | 0.767 | 0.767 | 0.744 | 0.799 | 0.022 | 0.194 | 0.224 | 1.391 | 0.199 | 4.2 |
| 0.45 | 0.795 | 0.796 | 0.772 | 0.823 | 0.010 | 0.183 | 0.234 | 1.393 | 0.199 | 4.4 |
| 0.50 | 0.830 | 0.831 | 0.792 | 0.846 | 0.005 | 0.161 | 0.242 | 1.405 | 0.203 | 4.8 |
| 0.55 | 0.834 | 0.834 | 0.809 | 0.850 | 0.004 | 0.153 | 0.252 | 1.401 | 0.201 | 5.2 |
| 0.60 | 0.830 | 0.831 | 0.820 | 0.880 | 0.004 | 0.156 | 0.262 | 1.398 | 0.200 | 5.7 |
| 0.65 | 0.844 | 0.843 | 0.824 | 0.874 | 0.009 | 0.144 | 0.273 | 1.405 | 0.203 | 6.1 |
| 0.70 | 0.848 | 0.847 | 0.828 | 0.860 | 0.015 | 0.124 | 0.280 | 1.397 | 0.200 | 6.5 |
| 0.75 | 0.837 | 0.837 | 0.827 | 0.865 | 0.028 | 0.130 | 0.290 | 1.394 | 0.199 | 6.9 |
| 0.80 | 0.837 | 0.838 | 0.820 | 0.856 | 0.047 | 0.103 | 0.300 | 1.393 | 0.199 | 7.3 |
| 0.85 | 0.826 | 0.826 | 0.806 | 0.841 | 0.066 | 0.105 | 0.312 | 1.392 | 0.199 | 8.1 |
| 0.90 | 0.792 | 0.793 | 0.780 | 0.806 | 0.099 | 0.095 | 0.326 | 1.400 | 0.201 | 8.8 |
| 0.95 | 0.753 | 0.752 | 0.745 | 0.770 | 0.148 | 0.085 | 0.344 | 1.399 | 0.201 | 9.7 |
| 1.00 | 0.628 | 0.628 | 0.633 | 0.647 | 0.278 | 0.073 | 0.384 | 1.407 | 0.203 | 11.9 |

Main grid (40 cells): $\max|\widehat{O} - O_{\mathrm{orac}}| = 0.0009$; $\max|\widehat{O} - O_{\mathrm{pred}}| = 0.0316$; $\max|\widehat{O} - O_{\mathrm{model}}| = 0.0251$ (medians), 0.0228 (means). For $O_\star < 1$, mean signed $(\widehat{O} - O_\star)$ is $-0.102$ under amplifying gain and $+0.099$ under suppressive gain, and mean $|\widehat{O} - O_\star|$ is 0.107 and 0.099. Fine grid (21 cells): $\max|\widehat{O} - O_{\mathrm{orac}}| = 0.0010$; $\max|\widehat{O} - O_{\mathrm{pred}}| = 0.049$; $\max|\widehat{O} - O_{\mathrm{model}}| = 0.050$ (medians), 0.043 (means).

| $C_6$ (main grid) | cells | mean $\lvert\widehat{O} - O_\star\rvert$ | mean $\lvert\widehat{O} - O_{\mathrm{pred}}\rvert$ | mean $\lvert\widehat{O} - O_{\mathrm{model}}\rvert$ |
|---|---|---|---|---|
| $< 2$ | 1 | 0.110 | 0.0018 | 0.0096 |
| 2–5 | 2 | 0.361 | 0.0316 | 0.0190 |
| 5–20 | 15 | 0.155 | 0.0071 | 0.0089 |
| $\geq 20$ | 22 | 0.055 | 0.0010 | 0.0010 |

The oracle comparison settles the estimator question on its own: across all forty conditions, recovery with the estimated frame is indistinguishable from recovery with the generating manifold's geometry at the same anchor. Whatever error remains is not gate 2's.

That error is anchor bias, and it is signed by the gain direction: $\widehat{O}$ underestimates $O_\star$ under amplifying gain and overestimates it under suppressive gain, by nearly equal amounts once the $O_\star = 1$ rows are set aside. Those rows have no gain leg, so the sign of the gain is undefined there and the amplifying and suppressive rows coincide.

The conditioning index decides how well the closed form predicts. With $C_6 \geq 20$ the recovered fraction matches both $O_{\text{pred}}$ and the model to 0.001 on average; between 5 and 20 to about 0.01; below 5 the errors reach several hundredths.

**The non-monotone condition.** Under suppressive gain at $k = 160$ and $\nu = 0.30$ the recovered fraction does not rise monotonically with $O_\star$. On the fine grid it climbs from 0.117 at $O_\star = 0$ to a plateau of 0.83–0.85 over $O_\star = 0.50$–$0.80$, then falls to 0.628 at $O_\star = 1$. Every cell of that block has $C_6$ between 1.9 and 11.9. The shape follows from the gain leg and the sagitta having opposite signs: $g + \delta$ crosses zero at $O_\star = 1 - (\delta/\nu)^2 \approx 0.56$ ($\delta \approx 0.20$), where the gain energy in the denominator vanishes; beyond it the uncancelled sagitta re-enters the denominator, fully so at $O_\star = 1$, where $\delta^2 = 0.04$ stands against $b^2 = 0.09$. The closed form and the anchor model both reproduce the rise, the plateau and the fall.

## S12 Cascade summary, with numbers

Table S18: The cascade of the main text, with the quantitative outcome of each validated gate. Derived from the tables above.

| gate | quantity | measured outcome |
|---|---|---|
| 0 | chart existence | artefact peaks at $h \approx 0.8\ell$ and is booked by stacking direction: radial stacking reads as gain (2.4× control), ambient as novelty (4.0× the floor) (Table S2) |
| 1 | $q = d - d_{\text{true}}$, leakage | noise-dominated: survival within 0.007 of the floor-corrected Haar law; the normal captures the first excess direction below $\sigma \approx \frac{1}{2}\ell^2/2R$, where $a$ collapses ($a^2 \approx 1-$capture) (Table S3) |
| 2 | $\|\hat{V}_d - V_d\|$; rotation | $\max\|\widehat{O} - O_{\text{orac}}\| \le 0.0010$ (Table S17); rotation $\approx \ell^2\kappa^2/3k$, i.e. $2/(3n_{\text{ref}})$ on a sphere, times a finite-$k$ factor: 1.04–1.19 on the patch (Table S5), 1.07–1.37 on the sphere with the areal $\ell^2$ (Table S4), largest at $k = 20$ |
| 3 | $(\ell^2/2)\vec{H}$, jitter $\ell/\sqrt{k}$ | ratio2 0.998–1.007 (Table S9), $1.000 \pm 0.005$ (Table S10); torus slopes 0.97–1.02 (Table S8); jitter 0.98–1.02 (Table S7) |
| 4 | $a = \|P_N \hat{\rho}\|$ | $a = 1.00$ on the sphere; gain-aligned anisotropy moves $G$ from 0.499 to 0.188 and $a$ to 0.48 at $\sigma_w/\ell = 1.56$ (Table S11) |
| 5 | $c = \hat{n}_g^\top \hat{H}$ | booking $\propto c^2 : 1 - c^2$ confirmed over $\|c\| = 1 \to 0.385$, ratios 0.98–1.02 (Tables S14, S15) |
| 6 | $C_6 = k\|r\|^2/\ell^2$ | mean $\|\widehat{O} - O_{\text{pred}}\|$: 0.001 at $C_6 \ge 20$, 0.007 at 5–20, 0.03 below 5 (Table S17) |
